# Microbial succession during wheat bran fermentation and colonisation by human faecal microbiota as a result of niche diversification

**DOI:** 10.1038/s41396-019-0550-5

**Published:** 2019-11-11

**Authors:** Kim De Paepe, Joran Verspreet, Christophe M. Courtin, Tom Van de Wiele

**Affiliations:** 1 Department of Biotechnology, Faculty of Bioscience Engineering, Center for Microbial Ecology and Technology (CMET), Ghent University, Ghent, Belgium; 2 Laboratory of Food Chemistry and Biochemistry, Leuven Food Science and Nutrition Research Centre (LFoRCe), Faculty of Bioscience Engineering, KU Leuven, Heverlee, Belgium

**Keywords:** Microbial ecology, Microbiome, Biofilms, Next-generation sequencing

## Abstract

The human gut can be viewed as a flow-through system with a short residence time, a high turnover rate and a spatial gradient of physiological conditions. As a consequence, the gut microbiota is exposed to highly fluctuating environmental determinants presented by the host and diet. Here, we assessed the fermentation and colonisation of insoluble wheat bran by faecal microbiota of three individuals at an unprecedented sampling intensity. Time-resolved 16S rRNA gene amplicon sequencing, revealed a dynamic microbial community, characterised by abrupt shifts in composition, delimiting states with a more constant community, giving rise to a succession of bacterial taxa alternately dominating the community over a 72 h timespan. Early stages were dominated by *Enterobacteriaceae* and *Fusobacterium* species, growing on the carbohydrate-low, protein rich medium to which wheat bran was supplemented. The onset of wheat bran fermentation, marked by a spike in short chain fatty acid production with an increasing butyrate proportion and an increased endo-1,4-β-xylanase activity, corresponded to donor-dependent proportional increases of *Bacteroides ovatus*/*stercoris*, *Prevotella copri* and *Firmicutes* species, which were strongly enriched in the bran-attached community. Literature and database searches provided novel insights into the metabolic and growth characteristics underlying the observed succession and colonisation, illustrating the potency of a time-resolved analysis to increase our understanding of gut microbiota dynamics upon dietary modulations.

## Introduction

The human gut harbours an indigenous microbial community characterised by a tremendous inter-individual variability, comprising around 100–160 different species within a single individual [1–6]. Most of the research initiatives directed towards the characterisation of this community provide snapshots of the faecal microbiota composition at a single point in time and the knowledge gap on intra-individual variability in microbiome composition is immense [7–9]. Longitudinal studies on the dynamics of the faecal microbiome from human individuals are sparse and often only include a limited set of time points [10].

The temporal variation in the gut microbiota composition is often studied early in life. The largest shifts occur during the first 3–4 years, when the gut microbial community establishes [11–13]. In terms of species absence/presence, the adult microbial community within one individual is relatively stable over time [2, 14–16]. The proportions of microbial taxa, however, are more subjected to temporal changes, reflecting a high responsiveness to perturbations, such as diarrhoeal episodes, distant travelling, antibiotic therapy and changing host conditions upon ageing [9, 14, 16–19]. One of the most influential and extensively studied forces shaping the human gut microbiota is the diet [14, 20–23]. With the exception of a few studies, the dietary impact on gut microbiota composition in human intervention trials is only assessed on the long-term and restricted to baseline and endpoint measurements [23–30].

Nevertheless, there are intriguing reports on the short-term responsiveness of the microbiome towards dietary transitions. Analysing faecal samples from an intervention study twice a week, Walker et al. [31] revealed a rapid response within 3–4 days. This timeframe was further narrowed in a study by David et al. [14] showing that a high fibre intake correlated with next-day enrichment of *Bifidobacteria*, *Roseburia* and *Eubacterium rectale*. These in vivo findings were confirmed in vitro by Aguirre et al. [32]. Obviously, substrate fermentation in the gut is a short-term process, confined by intestinal transit time, which can vary between individuals, but is in the order of 18 up to 60 h [33]. During gastrointestinal transit of a dietary substituent an ecological succession of primary colonisers, primary degraders, and cross-feeding microorganisms takes place, shaping the gut microbiome with every meal. This may have a long-term impact on microbiome composition and activity and eventually also the human host. Capturing the short-term dynamics of microbial succession can thus provide us with a better understanding of a fundamental process that underlies human gut microbiome composition. However, monitoring microbial dynamics requires highly frequent sampling. Despite the physiological relevance of human in vivo trials, frequent sampling of faecal microbiota or inaccessible gut regions is impossible. In vitro models allow frequent and regular sampling without ethical constraints [34], while the reduced complexity permits the study of specific dietary compounds at a high level of control, abstracting possible confounding effects due to the background diet or participant non-adherence [35].

In this study, we examined the time course of wheat bran colonisation and fermentation. The choice for wheat bran was made for its high dietary fibre content and for its recalcitrance to digestion in the upper gastrointestinal tract, making it available as a fermentation substrate for gut bacteria [31, 36–39]. Due to its insoluble nature and chemical complexity, wheat bran degradation requires the concerted action of a multitude of enzymes and involves a range of gut bacteria, which might act simultaneously or sequentially [36, 40–43]. We monitored the succession of gut microbiota members during wheat bran fermentation with time-resolved next-generation 16S rRNA gene amplicon sequencing. Both the luminal and the wheat bran-attached microbial populations were characterised and inter-individual differences were considered with separate incubations from three donors, selected from a preliminary screening experiment [40].

## Materials and methods

### Experimental set-up

Commercial wheat bran from the harvest of 2014 was supplied by Dossche Mills (Deinze, Belgium) and chemically characterised as described by De Paepe et al. [44] (Table S1). Upper digestive tract passage of wheat bran was mimicked through an in vitro batch digestion, adapted from Minekus et al. [45], as described by De Paepe et al. [40].

Subsequently, wheat bran was separately incubated with the human faecal microbiota derived from three healthy individuals, without a history of antibiotic use 6 months prior to the study. All three donors consumed a mixed Western diet. Donors were selected based on a divergent response to wheat bran in a previous experiment [40]. The donors differed in *Bacteroides* (donor 1), *Lachnospiraceae* (donor 2) and *Prevotella* (donor 3) signature and the nature of the produced SCFA. Donors 2 and 3 were characterised by a higher butyrate ratio in response to wheat bran, whereas in donor 1 propionate prevailed. The stool samples from donors 1 and 2 were sausage shaped (corresponding to Bristol Stool Score type3), while donor 3 produced a looser stool (corresponding to Bristol Stool Score type5). It was previously shown that in vitro incubation of the faecal sample for 48 h in a carbohydrate-low control medium induced shifts in the community but preserved richness and diversity [40]. Incubation research with faecal microbiota from human origin was approved by the ethical committee of the Ghent University hospital under registration number B670201214538. Faecal slurries were prepared as described by De Paepe et al. [40] and inoculated to obtain a final concentration of 2% faecal material (w/v) in penicillin flasks containing a carbohydrate-low medium supplemented with 1% wheat bran (w/v). The carbohydrate-low medium contained 3 g L^−1^ yeast extract (Oxoid Ltd, Basingstoke, Hampshire, UK), 1 g L^−1^ peptone (Oxoid Ltd, Basingstoke, Hampshire, UK) and 1 g L^−1^ mucin from porcine stomach Type II (Sigma Aldrich, St. Louis, MO, US), dissolved in 0.1 M phosphate buffer at pH 6.8 [40]. A control without wheat bran was taken along for donors 2 and 3. The penicillin flasks were flushed with N_2_/CO_2_ during 30 cycles to generate anaerobic conditions and set at atmospheric pressure before incubation at 37 °C and 90 rpm on an orbital shaker (KS 4000 i control, IKA, Staufen, Germany).

The course of fermentation and wheat bran colonisation was followed up over time. Samples were taken 1 h after inoculation, in 2-h time intervals the first 24 h after inoculation and after 48 and 72 h. For each time point and each donor, a separate tube was incubated in duplicate (further referred to as a biological replicate), as shown in Figure S1. At the foreseen sampling time point, the gas pressure and pH were measured. The unfermented wheat bran residue was collected by centrifugation for 5 min at 700 × *g* and stored in 0.1 g aliquots at −20 °C for DNA extraction, after washing with phosphate buffer to remove nonattached and loosely attached microorganisms, as described by De Paepe et al. [40]. Liquid samples (‘luminal samples’) were aliquoted for DNA extraction (pellet obtained after centrifuging 250 µL at 5000 × *g* for 10 min), enzyme activity and short chain fatty acid (SCFA) analysis after storage at −20 °C (Fig. S1). For the enzyme activity measurement, 2 mL sample was centrifuged for 10 min at 8000 × *g*. The supernatant, representing the extracellular enzyme fraction, was stored at −20 °C. The pellet was washed twice with 2 mL wash buffer (phosphate buffer pH 7, 50 mM), which was removed again by centrifugation at 8000 × *g* for 10 min. The pellet was stored at −20 °C. Prior to analysis 1.5 mL phosphate buffer (pH 7, 50 mM) and 200 mg glass beads (0.11 mm Sartorius, Göttingen, Germany) were added to the pellet. Cell extracts, containing the membrane-associated and intracellular enzyme fraction, were obtained through mechanical lysis by multidirectional beating for two times 40 s at 1600 rpm in a FastPrep-96 instrument (MP Biomedicals, Santa Ana, CA). α-l-arabinofuranosidase and β-xylosidase activity were determined spectrophotometrically, using 4-nitrophenyl-β-d-xylopyranoside and 4-nitrophenyl-α-l-arabinofuranoside as artificial substrates (Sigma Aldrich, St. Louis, MO, US). 2.5 mM stock solutions of these substrates were freshly prepared in phosphate buffer (pH 7, 50 mM). One hundred µL aliquots of these stock solutions were incubated in a 96-well plate with 100 µL sample (1:10 diluted in phosphate buffer pH 7, 50 mM). The enzymatic release of 4-nitrophenol was measured every 30 min for a period of 24 h at 405 nm, 37 °C with an Infinite 200Pro plate reader (Tecan, Männedorf, Switzerland). Enzyme activities were expressed as the concentration 4-nitrophenol (µM), inferred from the OD by reference to a 4-nitrophenol standard curve, released per minute in the linear range (up to 4 h) at pH 7 and 37 °C. The endo-1,4-β-xylanase enzyme activity was determined using the Xylazyme-AX assay (Megazyme, Bray, Ireland). Two hundred µL sample was diluted 2.5 times in McIlvaine buffer pH 6.8 and incubated for 4 h at 37 °C with a Xylazyme-AX tablet. Enzyme activities were expressed as the change in extinction values h^−1^ mL^−1^ sample measured at 590 nm (E590) compared to a blank without tablet, corrected by subtracting the E590 values of the buffer without sample. SCFA levels were measured using capillary gas chromatography coupled to a flame ionisation detector (Shimadzu, Hertogenbosch, the Netherlands) after diethyl ether extraction, as described by De Paepe et al. [40]. Samples were twofold diluted with milli-Q water.

### Microbial community analysis

The microbial community was analysed using Illumina next-generation 16S rRNA gene amplicon sequencing. Both the luminal suspension and the washed bran residue were analysed for the time points 1, 2, 4, 6, 8, 10, 12, 16, 20, 24, 48 and 72 h (Fig. S1). Biological replicates were only analysed from time points 2, 10 and 48 h.

DNA was extracted and DNA quality verified as described by De Paepe et al. [40]. Samples were sent out to LGC Genomics (Teddington, Middlesex, UK) for library preparation and sequencing on an Illumina Miseq platform. To assess sequencing quality, a mock community sample, with a composition as listed by De Paepe et al. [40] was included in triplicate.

The V3–V4 region of the 16S rRNA gene was amplified by PCR using primers derived from Klindworth et al. [46], with a slight modification to the reverse primer, 785R (5′-GACTACHVGGGTATCTAAKCC-3′), by introducing another degenerated position (K) to make it more universal. The library preparation was performed, as described by De Paepe et al. [40]. The sequence data have been submitted to the NCBI (National Center for Biotechnology Information) database under accession number SRP127353.

### Bio-informatics and statistical analysis

The mothur software package (v.1.39.5) and guidelines were used to process the amplicon data, as outlined in the Supplementary information [47]. The OTU table with taxonomy assignment according to RDP version 16 was loaded into R, version 3.4.1 (2017-06-30) [48]. Singletons were removed [49]. For the most abundant OTUs the sequences retrieved from the 3% dissimilarity level fasta file, obtained in mothur, were classified using the RDP SeqMatch tool, restricting the database search to type strains with only near-full-length, good quality sequences and blasted in NCBI against the 16S rRNA gene sequences, selecting only type material, with optimisation of the BLAST algorithm for highly similar sequences (accession date: September 2017) [50–52]. Although identification to the species level based on short 300 bp reads can be equivocal, the most likely species classification of some interesting OTUs is reported in the results and discussion section. In the event of inconsistencies in the results of the RDP SeqMatch tool and NCBI BLAST, no species level classification is provided. The results for the most abundant OTUs are shown in Table S2.

Statistical analysis was performed in R, version 3.4.1 (R [48]). Details regarding the applied ordination and clustering methods, network inference tools and formal hypothesis tests are provided in Supplementary Information.

## Results

Wheat bran colonisation and fermentation by the faecal microbiota from three healthy individuals, selected based on their diverging response to wheat bran, were followed up through time by means of SCFA and enzyme activity analysis and 16S rRNA gene amplicon sequencing of the luminal and bran-attached microbial community (Fig. S1).

**Fig. 1:**
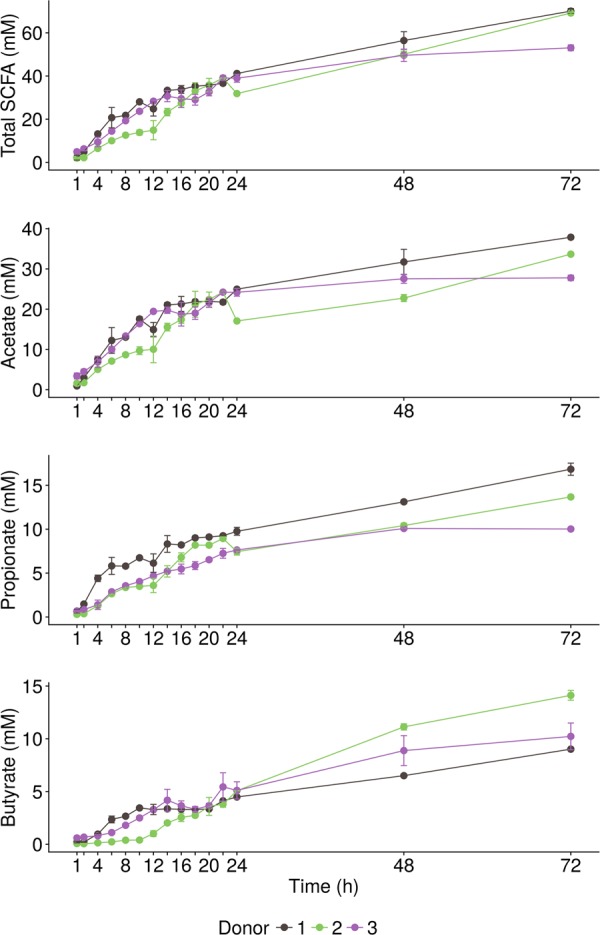
SCFA production (*n* = 2 biological replicates) resulting from the incubation of wheat bran in the presence of a carbohydrate-low medium with the faecal material of three different donors for up to 72 h

The three donors fermented wheat bran, present in a carbohydrate-low medium, at a different rate and to a different extent (Fig. 1). In donor 2, fermentation proceeded in two phases, reaching a plateau after the first 12 h, marking the depletion of components from the carbohydrate-low medium and the onset of wheat bran fermentation (Fig. 1, S2). In donors 1 and 3, the SCFA production increased more rapidly but levelled off 12–20 h after inoculation (Fig. 1). A comparison with the carbohydrate-low medium (Control) in donor 3 showed that wheat bran fermentation already kicked-off after 6 h but became more efficient when the carbohydrate-low medium was depleted after 20 h (Fig. S2). While donor 2 fermented wheat bran initially at a slower rate, after 72 h, the total SCFA levels produced by donor 2 (69.2 ± 0.6 mM) were similar to donor 1 (70.1 ± 1.2 mM) and higher compared to donor 3 (53.1 ± 1.3 mM) (Fig. 1). Acetate production most rapidly increased after inoculation, followed by propionate production. Butyrate production was delayed with a lag phase of 4 up to 10 h depending on the donor, resulting in an increasing butyrate to propionate ratio over time (Fig. S3). In donors 2 and 3, butyrate production approached or exceeded the propionate production after 48 h. In donor 1, on the other hand, propionate production was higher than butyrate at all time points. Branched SCFA production indicated that donor 3 displayed the lowest proteolytic activity (Fig. S4).

**Fig. 2:**
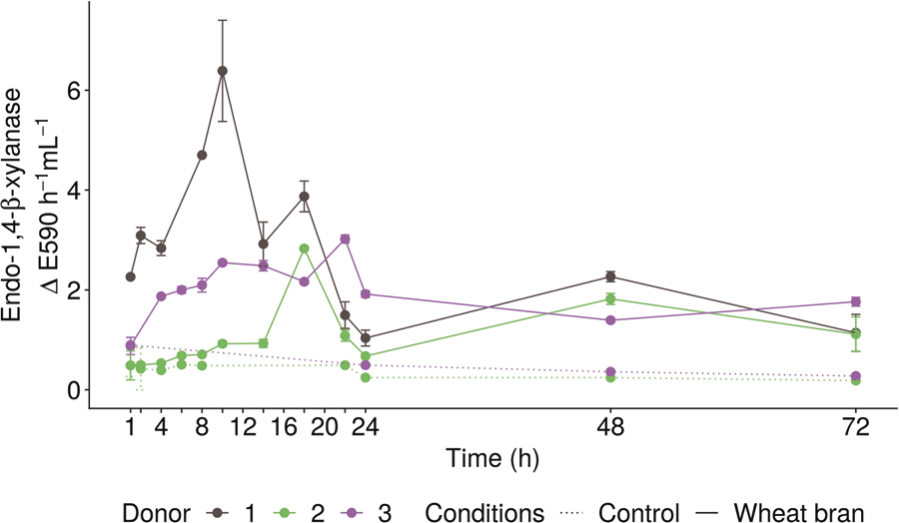
Endo-1,4-β-xylanase enzyme activity (*n* = 2 biological replicates) measured in the membrane-associated and intracellular fraction of liquid samples, consisting of a carbohydrate-low medium incubated with the faecal material of three different donors for up to 72 h in the absence (dotted line, Control) or presence (solid line, wheat bran) of wheat bran. Enzyme activity was expressed as the increase in extinction values measured at 590 nm (E590) per hour of incubation and per mililitre of sample

The slow donor-dependent fermentation of wheat bran was reflected in a donor-dependent increase of the endo-1,4-β-xylanase activity, required for the primary degradation of wheat bran arabinoxylans, in the combined intracellular and membrane-associated enzyme fraction over time (Fig. 2). The slow onset of wheat bran fermentation in donor 2 corresponded to an initially lower enzyme activity compared to donors 1 and 3. After 14 h, however, a sudden peak in endo-1,4-β-xylanase activity was observed, paralleling the increment in SCFA production (Figs. 1, 2 and S2). A similar peak was observed in donor 1 after 8–10 h, whereas in donor 3 a more gradual increase occurred with a smaller peak after 20 h, coinciding with a further rise in SCFA production compared to the control (Fig. 2 and S2). The constant and low endo-1,4-β-xylanase activity levels (E590 < 0.2 after 72 h) in this control (Fig. 2) indicate that endo-1,4-β-xylanase activity depended on the presence of wheat bran and the relation with the SCFA production illustrates that endo-1,4-β-xylanase is essential for the fermentation of wheat bran. Besides xylanase, other enzymes including β-xylosidase and α-l-arabinofuranosidases are involved in the further depolymerisation of the substrate to fermentable xylose and arabinose monomers. These enzymes displayed a more variable activity, which was also present in the control (Fig. S5), indicating a less tight regulation of their activity by the wheat bran substrate. The extracellular enzyme fraction was not influenced by wheat bran supplementation. The extracellular enzyme activity was negligible after 72 h (E590 < 0.2 and a p-nitrophenol production < 0.06 µM min^−1^) compared to the intracellular and membrane-associated fractions for all enzymes and followed a similar decreasing pattern over time in the control and wheat bran incubations, indicating that this activity originated from enzymes released through cell disintegration in the faecal samples, exhibiting the highest extracellular activity (E590 > 3 and p-nitrophenol production > 4 µM). Considering this low extracellular enzyme activity and given the required extracellular action of endo-1,4-β-xylanase activity, this enzyme was likely membrane-associated, whereas β-xylosidase and α-l-arabinofuranosidase enzymes can act intracellularly.

**Fig. 3:**
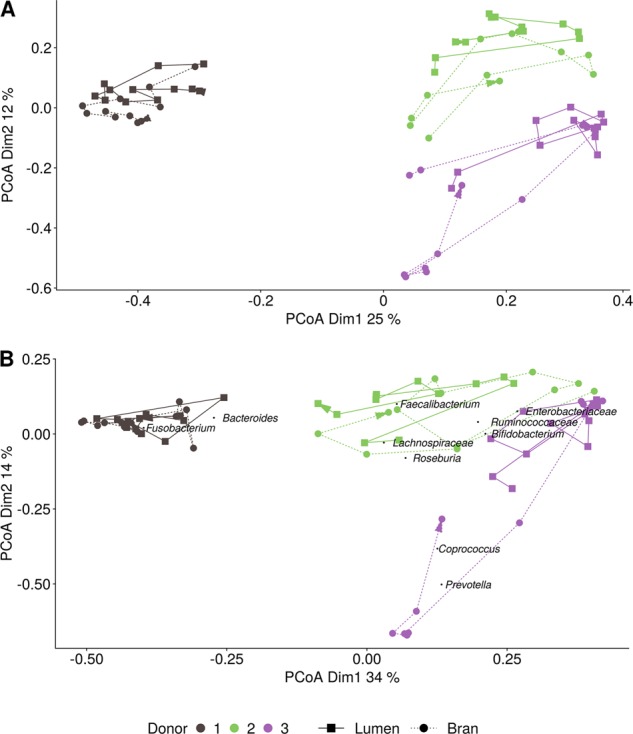
Principle coordinate analysis (*n* = 1) of the species (**a**) and genus (**b**) level luminal (squares) and wheat bran-attached (circles) microbial community composition resulting from the incubation of wheat bran with the faecal material of three different donors for up to 72 h. The abundance-based jaccard dissimilarity matrix was used and weighted average scores of the most abundant genera were *a posteriori* projected, by means of the wascores function from the package vegan

The evolution of the microbial community composition over time was assessed for each of the three donors individually, as the PCoA showed that the gut microbiota composition of the three donors differed considerably at species and genus level (Fig. 3). Furthermore, network analysis revealed that only 3 of the 238, respectively, 19 of the 2503 co-occurrence interactions (edges) were common between the three donors at genus, respectively, species level (Fig. S6). For each donor, a further distinction was observed between the communities from the luminal and bran niche, which differed from one another in terms of global community composition and co-occurrence interactions (Figs. 3, S6). For each donor and niche, the variation in the community structure over time was analysed. Both the luminal and bran environment for all three donors displayed a very dynamic microbial community.

**Fig. 4:**
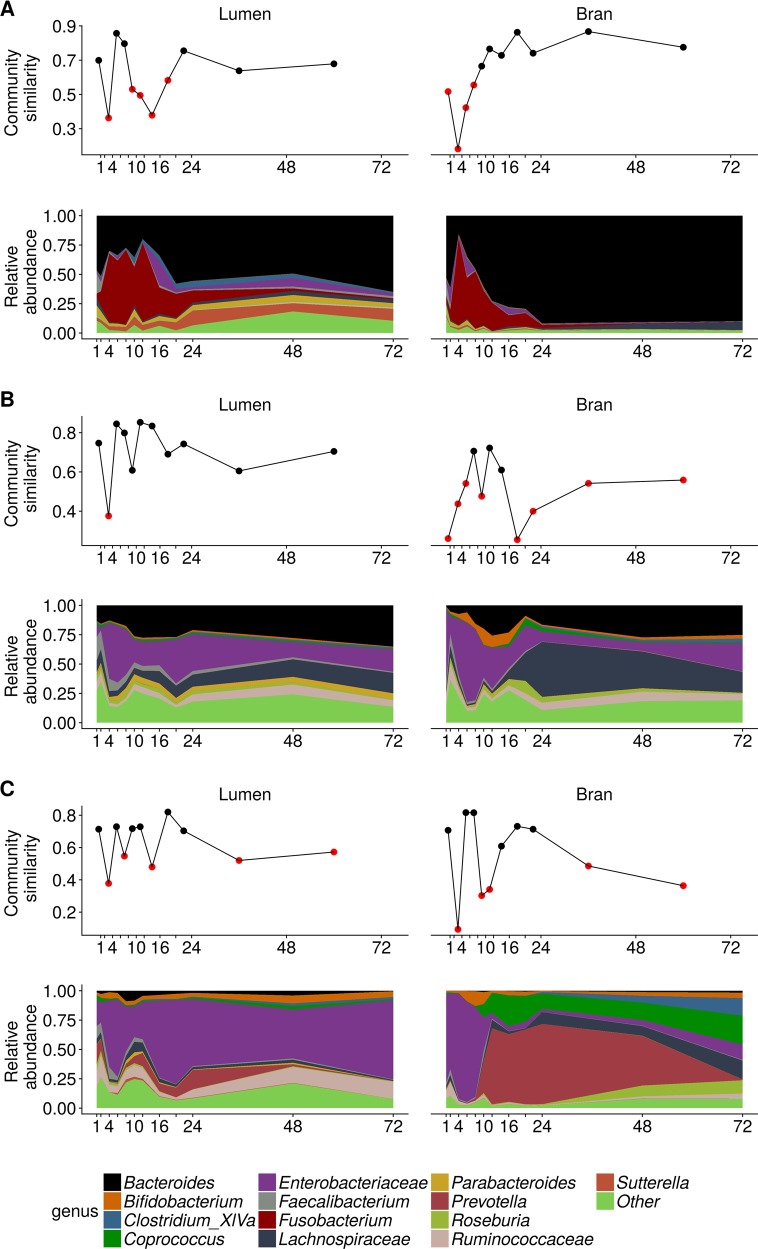
Genus level luminal and bran-attached community composition and community similarity between consecutive time points of donor 1 (**a**), donor 2 (**b**) and donor 3 (**c**) (*n* = 1). The similarity value was calculated by subtracting the abundance based jaccard dissimilarity metric from 1. Similarity values below 0.6 are indicated in red

**Fig. 5:**
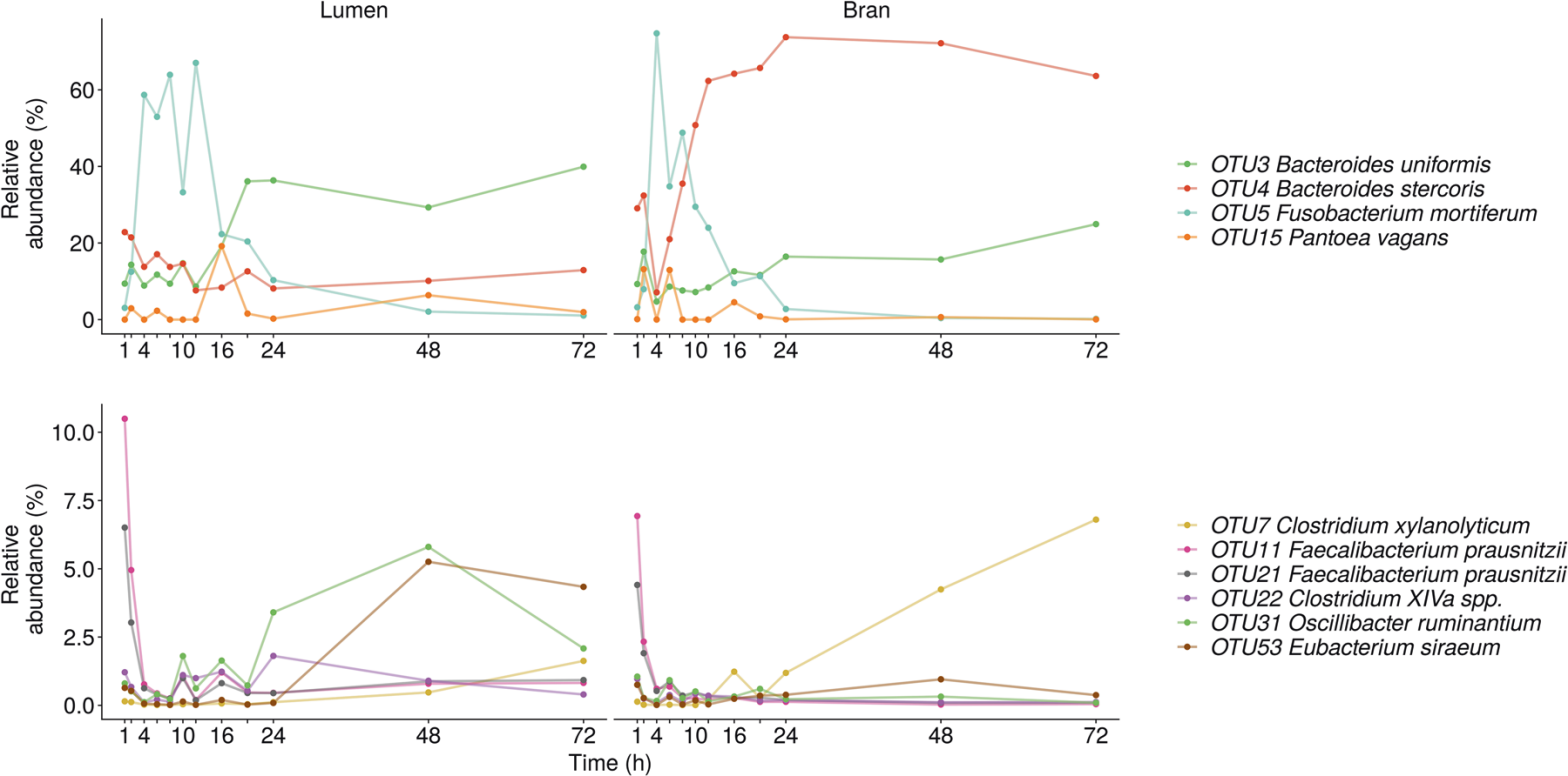
Species level luminal and bran-attached community composition of donor 1 (*n* = 1)

In donor 1, the luminal microbial community radically changed between 2 and 4 h after inoculation and after a relatively stable period up till 8 h, large fluctuations occurred between 8 and 20 h after inoculation (Figs. 4a, S7a and S8a). The bran-attached community in donor 1 was less volatile and only displayed a major shift between 2 and 4 h after inoculation (Figs. 4a, S7a and S8a). In the luminal community, after 2 h, *Fusobacterium mortiferum* (OTU5) sharply increased in relative abundance at the expense of *Bacteroides uniformis* (OTU3), *Bacteroides stercoris* (OTU4) and *Faecalibacterium prausnitzii* (OTU11, 21) relative abundances (Fig. 5). Between 12 and 16 h, this trend was reversed and the microbial community evolved towards a stable, non-variable proportional composition, dominated by *B. uniformis* (OTU3) and *Firmicutes* species belonging to *Clostridium* cluster XIVa (OTU22, 46), *Oscillibacter* (OTU31) and after 24 h also *Clostridium* cluster IV (OTU53) (Fig. 5). In the bran-attached community, *Fusobacterium* was gradually replaced by *B. uniformis* (OTU3), *B*. *stercoris* (OTU4) and *Clostridium xylanolyticum* (OTU7). The latter two species showed a clear preference for the bran environment (Fig. 5).

**Fig. 6:**
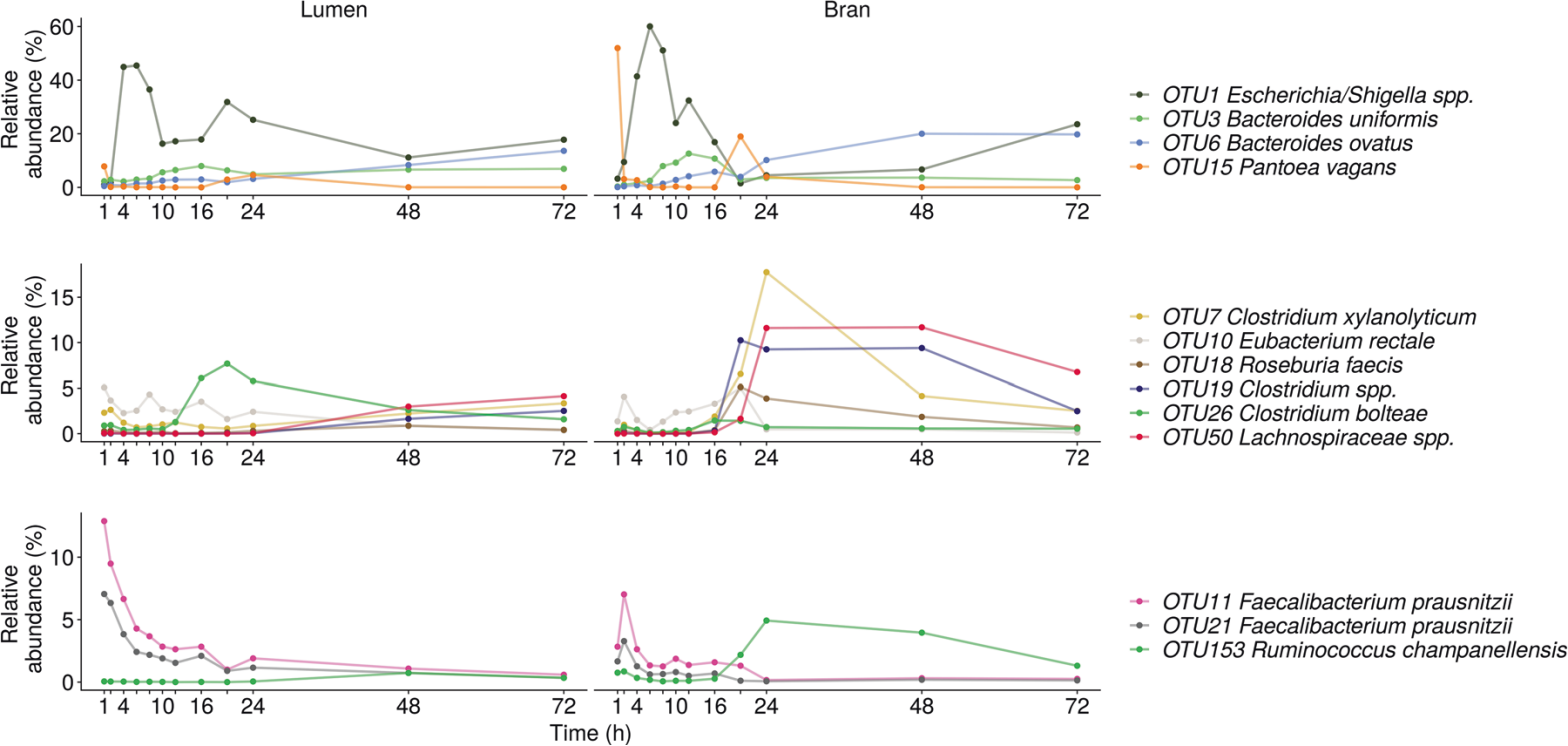
Species level luminal and bran-attached community composition of donor 2 (*n* = 1)

In donor 2, an abrupt change presented itself in the luminal community between 2 and 4 h after inoculation under the form of a largely increased proportional abundance of *Escherichia/Shigella* spp. (OTU1) and a drop in *Firmicutes* species within the genera *Faecalibacterium* (OTU11,21), *Lachnospiraceae* (OTU10,50), *Ruminococcaceae* (OTU153), *Clostridiales* (OTU7,19,26) and *Roseburia* (OTU18) (Figs. 4b, 6, S7b and S8b). Shifts in *Enterobacteriaceae*, comprising *Pantoea vagans* (OTU15) next to E*scherichia-Shigella* spp. (OTU1), were also responsible for the large community variation observed in the timespan between 8 and 10, 16 and 20 and 24 and 48 h (Figs. 4b, 6, S7b and S8b). During these periods *Enterobacteriaceae* reductions corresponded to elevated levels of *B. uniformis* (OTU3), *Bacteroides ovatus* (OTU6), *Lachnospiraceae* (OTU10,50), *Clostridiales* (OTU7,26), *Ruminococcaceae* (OTU153) and *Roseburia* (OTU18) (Figs. 4b, 6, S7b, and S8b). The transitions in the luminal community coincided with a big turnover in the bran-attached community (Figs. 4b, 6, S7b, and S8b). The first 16 h, the community composition was dictated by a trade-off between *Firmicutes* (OTU7,11, 18, 21, 50) and *Enterobacteriaceae* (OTU1,15) with a slightly increasing *Bacteroides* share, mainly attributed to *B. uniformis* (OTU3) (Figs. 4b, 6, S7b and S8b). After 20 h, *Lachnospiraceae* (OTU7,18,19,26,50) and *Ruminococcaceae* (OTU153) suddenly appeared at high abundance in the bran-attached community and *B. ovatus* (OTU6) displaced *B. uniformis* (OTU3) as the dominant bran colonising *Bacteroides* species (Fig. 6).

**Fig. 7:**
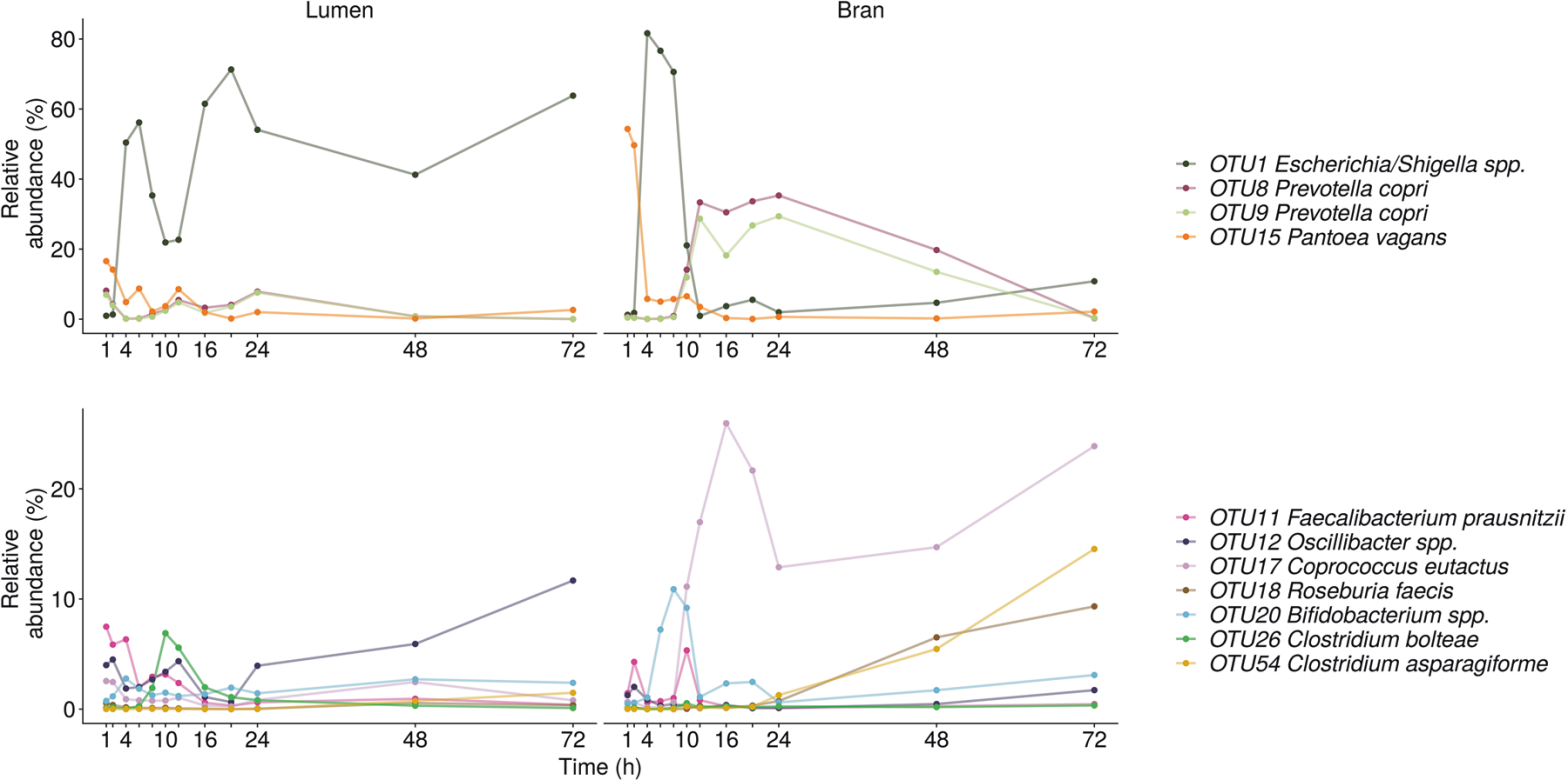
Species level luminal and bran-attached community composition of donor 3 (*n* = 1)

In donor 3, similar as in donor 2, *Proteobacteria* and *Firmicutes* levels alternately increased in the luminal community, causing the peaks in global variability in the timeframes from 2–4, 6–8, and 12–16 h (Fig. S7c). *Proteobacteria* were represented by *Pantoea vagans* (OTU15) during the first two hours followed by *Escherichia-Shigella* spp. (OTU1) (Fig. 7). In the time interval between 8 and 14 h and after 20 h, *Oscillibacter* spp. (OTU12) reached a high proportional abundance (Fig. 7). *Bacteroidetes* abundances remained low in the luminal community throughout time (Fig. S7c). In the bran environment, however, *Prevotella copri* (OTU8,9) substituted the initially dominating *Proteobacteria*, after 10 h (Figs. 4c and 7). Starting at 48 h, diverse *Firmicutes* members within the *Coprococcus* (OTU17), *Lachnospiraceae* (OTU26), *Clostridium* cluster XIVa (OTU54) and *Roseburia* (OTU18) genus efficiently colonised the bran (Figs. 4c and 7).

The observed transitions were very abrupt and delimited periods with a more gradually changing community composition (Figs. 4, S7 and S8), which is reflected in the distinctive clustering of time points characterised by a large contrast between the high within-cluster similarity and low between-cluster similarity (Fig. S9). The magnitude of community variation over time, i.e. an abrupt transition versus a more gradual change, could not be explained by the sampling frequency, as indicated by time decay analysis (Fig. S10). The community transitions also largely affected the co-occurrence of individual community members, as can be seen from the networks for all donors and niches (Figs. S11–S16).

**Fig. 8:**
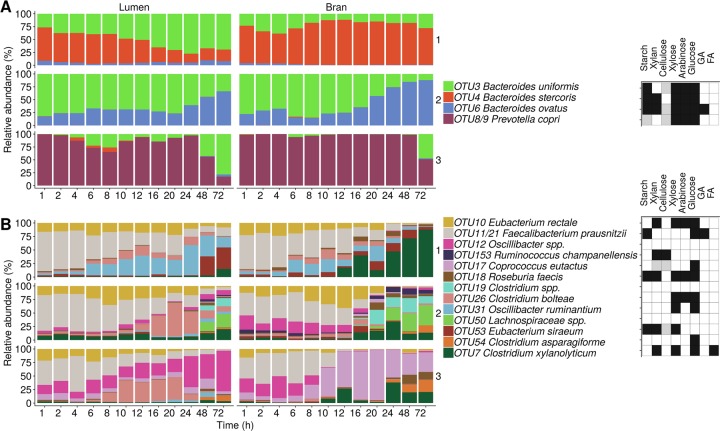
Luminal and bran-attached community composition of the most abundant species, characteristic for the three donors. Relative abundances of the displayed taxa were normalised and rescaled to 100% within the *Bacteroidetes* (**a**) and *Firmicutes* (**b**) phylum (*n* = 1). The experimentally verified (black fill colour) and predicted putative (grey fill colour) capacity of species to degrade wheat bran derived carbohydrates is indicated in a heat map. No fill colour means that there is no information available that the concerned species is capable of degrading the compounds. Taxa, indicated by spp., which could not be identified to species level, were not considered for the functional annotation. GA glucuronic acid and FA ferulic acid. An extensive functional/physiological characterisation can be found in Supplementary Tables S3–S7

Network inference confirmed the dominance of *Bacteroides* and *Fusobacterium* in donor 1 and *Enterobacteriaceae* in donors 2 and 3, which translated itself in significant negative co-occurrence interactions with other taxa at genus level both in the bran and luminal community (Figs. S11–S16). In contrast, interactions between genera belonging to the *Firmicutes* phylum were mostly positive. The higher degree of co-existence in the *Firmicutes* phylum compared to the *Bacteroidetes* phylum, was also apparent at species level (Fig. 8). The *Bacteroidetes* community is characterised by the dominance of a single species at all time points.

Finally, to investigate the importance of stochastic effects in the wheat bran colonisation and fermentation process, biological duplicates were analysed. The fermentation products were characterised by a low coefficient of variation of 10% for acetate, 7% for propionate, 9% for butyrate and 21% for branched SCFA, averaged over all time points (*n* = 2) (Fig. S17a). The amplicon next-generation sequencing results from biological replicates at three time points (2, 10 and 48 h) displayed a higher dispersion, around 50% at species level (Fig. S17c). This observed dispersion is, however, attributable to both sources of technical and biological variation. Technical variation was estimated by comparing mock community sequencing data (*n* = 3), ruling out biological variation, as the same biological sample was included in triplicate in the sequencing run. The dispersion of the mock community samples was in the same range as those of most biological replicates, suggesting that technical variation accounts to a large extent for the observed dispersion (Fig. S17b, c). Despite the dispersion, replicates clustered together indicating that the wheat bran effects on the microbial community are reproducible (Fig. S17d, e).

## Discussion

Succession is a widely used ecological concept describing a course of changes in species composition over time in response to environmental perturbations [53]. The concept emerged from the observation of plant communities, but has since been explored in many other domains, including microbiology [54, 55]. Well-studied examples of temporal succession in the host-microbe interphase include the colonisation of the infant gut and dental plaque formation [11, 13, 56, 57]. In this study, the supplementation of wheat bran as a growth and colonisation substrate resulted in a dynamic microbial succession, characterised by an initial proportional reduction in *Faecalibacterium*, *Lachnospiraceae*, *Ruminococcaceae*, *Roseburia* and *Clostridium* cluster XIVa in all three donors, corresponding to a donor-dependent increase of *Fusobacterium* and *Enterobacteriaceae* proportions. These high proportions were in most cases not persistent and gave way to a donor-dependent rise in *Bacteroides*, *Prevotella* and *Firmicutes* over time.

The observed microbial succession was not random, as the microbial community, sampled from separate tubes for each time point, converged to a constant community composition in between shifts. Furthermore, analysis of biological duplicates (for time points 2, 10, and 48 h) and a mock community in triplicate indicated that the dispersion, as measured by the coefficient of variation, was mostly imposed by technical variation. Despite the technical noise, biological replicates clustered together indicating that the microbial succession was determined by underlying biological mechanisms. We have explored these possible underlying mechanisms, by reviewing the available literature on the metabolic potency and preferred growth conditions of the most important taxa observed in this study (Supplementary Information; Tables S3–S7). Minimum generation time and oxygen sensitivity were considered as factors that may affect the onset of growth (Supplementary Information; Table S3). pH optimum and tolerance were examined, as we have demonstrated a pH drop (from 6.8 to 6.2 after 72 h) as a consequence of the increased SCFA production over time (Supplementary Information; Table S3). The most essential requirement for the expansion of a population, however, is the metabolic capacity to breakdown a substrate and efficiently assimilate the released monomers and the obtained energy into new biomass. From a comparison of the SCFA profiles in the wheat bran supplemented condition with the control, containing only the carbohydrate-low medium without wheat bran, we have established that nutritional conditions changed as the primary colonisers preferentially consumed the carbohydrate-low medium. Depletion of this medium, consisting of 3 g L^−1^ yeast extract, 1 g L^−1^ peptone and 1 g L^−1^ mucin, prompted the wheat bran colonisation and fermentation by a different set of species. This was reflected by an increase of the endo-1,4-β-xylanase activity over time, which was not observed in the control, suggesting that this enzyme activity is inextricably linked to the wheat bran fermentation. This is underscored by the known function of endo-1,4-β-xylanase in the primary degradation of arabinoxylans, which are a major constituent of wheat bran. Finally, the role of wheat bran as a driver for the observed succession, was further supported by our finding of a different succession pattern on the insoluble wheat bran residue compared to the luminal environment. The driving force behind wheat bran attachment is unclear, but we previously suggested that bacteria deploying the enzymatic capacity to degrade wheat bran could be expected to be located in close proximity of the insoluble substrate [40]. Indeed, Macfarlane et al. [58] reported that the xylanase, β-xylosidase, α-arabinofuranosidase enzyme activity of bacteria desorbed from food particles is significantly higher compared to non-particulate bacteria [58]. To evaluate if the wheat bran-attached species in this study have the metabolic capacity to degrade wheat bran carbohydrates, mainly consisting of arabinoxylans and cellulose, a combined literature and database search was performed (described in detail in the Supplementary information; Tables S5–S7). The mechanism of attachment to the insoluble wheat bran substrate are currently unresolved, but could encompass a cellulosome enzyme system in case of *Ruminococcus champanellensis* and common adhesive structures such as pili, fimbriae and extracellular polymeric substances, which were all reviewed in Table S4 [59–62].

In donor 1 *F. mortiferum* (OTU5) was predominant in the early stages of incubation in the luminal and bran-attached microbial community. *F. mortiferum* is a poorly characterised frequently encountered human gut resident, with established adhesive properties and capable of proteolytic and saccharolytic fermentation (Tables S3 and S7). There is, however, no substantial evidence for the breakdown of complex polysaccharides, such as those present in wheat bran (Tables S5 and S7). In line with this, *Fusobacterium* proportions started to decline in the bran-attached community after 4 h and sharply dropped in the luminal community after 16 h, after which the community was dominated by *Bacteroides* species, which are reported to ferment xylans (Table S7). *B. uniformis* was most abundant in the luminal community, which is in agreement with the four-fold proportional increase obtained by Duncan et al. [36] after 24 h of incubation of a faecal sample in the presence of wheat bran. In line with our previous results, *B. stercoris* was more successful in colonising the bran [40].

In donors 2 and 3, *Escherichia*/*Shigella* spp. OTU1 was an important primary coloniser. This species reached its maximum relative abundance 4–8 h after inoculation and sharply decreased in abundance afterwards, except in the luminal community of donor 3. *Enterobacteriaceae* are fast growing facultative anaerobic opportunistic pathogens, which are phylogenetically closely related (Table S3) [63, 64]. Therefore it comes as no surprise that OTU1 could not be unambiguously classified at species level and showed a similar identity to *Escherichia fergusonii*, *Shigella sonnei*, *Shigella flexneri* and *Escherichia coli* (Table S2). These species are capable of proteolytic fermentation and produce an extensive CAZyme complement, but are not able to degrade wheat bran arabinoxylans or cellulose (Tables S5–S7).

In donor 2, the decreasing *Enterobacteriaceae* abundance corresponded to a rise in *B. uniformis* like in donor 1, and *B. ovatus*, which was most efficiently colonising the bran surface. *B. ovatus* is a well-characterised xylanolytic bacteria, capable of growing on wheat bran arabinoxylans and possessing membrane-associated xylan utilisation systems (Xus) directed to the efficient utilisation of soluble xylan through the coordinated cellular attachment, depolymerisation, transport and further intracellular degradation of oligosaccharides [41, 65–67]. This efficient integrated enzyme system also confers an important competitive advantage by avoiding the loss of monosaccharides to other bacteria. Xus have not been extensively experimentally characterised. However, the genes encoding these multi-enzyme complexes are arranged in polysaccharide utilisation loci (PUL) with a conserved organisation, which can be predicted from genome sequence data [68]. Interestingly, donor 3 was characterised by an enrichment of *P. copri* (OTU8/9) on the bran. This species has also been predicted to carry 6 PULs, containing genes encoding enzymes involved in xylan and cellulose degradation [68] (Table S5 and Fig. 8).

*Firmicutes* species occurred at low relative abundances during the first 16–20 h, which is reflected in a lagged butyrate production. After this period, possibly favoured by the low pH, the luminal community was characterised by increasing proportions of species related to *Oscillibacter ruminantium* (OTU31) and *Eubacterium siraeum* (OTU53) in donor 1, *Clostridium bolteae* (OTU26) in donor 2 and *Oscillibacter* spp. (OTU12) in donor 3. The bran was preferentially colonised by *C. xylanolyticum* (OTU7) in donors 1 and 2, *Lachnospiraceae* spp. (OTU50), *Clostridium* spp. (OTU19) and *R. champanellensis* (OTU153) in donor 2 and *Coprococcus eutactus* (OTU17), *Roseburia faecis* (OTU18) and *Clostridium asparagiforme* (OTU54) in donor 3. Most of the identified *Firmicutes* species were not represented in the CAZy database, reflecting their understudied carbohydrate utilisation capacity and contributing to the misconception that *Firmicutes* do not play a significant role in carbohydrate utilisation [69]. Recently, it has been suggested that the lower density of genes encoding CAZymes in *Firmicutes* species is a consequence of a high substrate specificity, determined at species and even strain level [69]. Indeed, in this study, we found species with the reported ability to grow on xylan or xylose comprising *C. xylanolyticum*, *R. faecis*, *C. bolteae*, *O. ruminantium*, *E. siraeum* co-existing with species capable of using cellulose including *R. champanellensis* and *C. eutactus* (Table S7, Fig. 8). Interestingly, the latter two species are limited in their ability to ferment other carbohydrates and even simple sugars, which was more common among the other species (Table S7). Additionally *C. asparigiforme* displays α-glucuronidase activity, required for the removal of 4-O-methyl-D-glucuronic acid moieties linked to xylose and *C. xylanolyticum* metabolises ferulic acid substituents of arabinose residues in wheat bran arabinoxylans (Table S7 and Fig. 8). *R. faecis*, moreover, is able to cross-feed on acetate to produce butyrate (Tables S5 and S7) [70, 71]. Butyrate production was related to the proportion of butyrate producing species (Table S7), which was low in donor 1 and reached higher proportions in donors 2 and 3 (*R. faecis* and *C. eutactus*).

In summary, during the first stages of incubation *Enterobacteriaceae* and *Fusobacterium* species dominated the microbial community. These primary colonisers share many characteristics such as a fast onset of growth at a neutral pH optimum, oxygen tolerance, adhesive properties, bacteriocin production and a proteolytic and saccharolytic catabolism, mostly directed towards oligosaccharide degradation (Tables S3–S7). We therefore hypothesise that these primary colonisers grow well on the supplemented protein rich, carbohydrate-low medium. This is also reflected by their decreased abundance over time, probably due to depletion of the carbohydrate-low medium in combination with acidification. During a second stage of fermentation, characterised by a spike in SCFA production and endo-1,4-β-xylanase enzyme activity, these pioneer species were succeeded by *Bacteroidetes* and *Firmicutes* species, which were primarily thriving on the insoluble wheat bran residue. The enrichment of these taxa, possessing the necessary enzymatic capacity to degrade wheat bran, strongly suggests that the succession, at least on the insoluble wheat bran particles, is driven by the nutritional role of wheat bran, acting as a distinct microbial niche. Both *Bacteroides* and *Prevotella* species are tailored to breakdown wheat bran by virtue of their extensive glycolytic genomes enriched in xylanolytic genes, whereas the *Firmicutes* species are less versatile compared to the *Bacteroides* species and the capacity to degrade xylan, cellulose, starch, ferulic acid or to cross-feed is more divided across species. This high level of specialisation may explain the co-existence of *Firmicutes* species with a distinct substrate specificity, in contrast to the manifestation of a sole dominant *Bacteroidetes* species, resulting from their overlapping glycan degrading capacity, which is in line with the inverse relation between *Bacteroides* and *Prevotella* observed by others and the positioning of *Bacteroides* as glycan degrading generalists [1, 72–75].

We realise that the presented in vitro experiment and hypotheses are an oversimplification in the face of the continuous dietary influx and changing host conditions challenging the gut bacteria in vivo. The proposed mechanisms for the microbial succession are speculative given the uncertainty of species level classification based on short-length amplicon reads, the scarce literature data for some of the discussed species and consequent extrapolation of growth characteristics and conditions from close relatives, the automated prediction of growth and metabolic features from genomic information and the underrepresentation of some species in databases. Further research is undoubtedly needed. However, this is to our knowledge the first in-depth study of the short-term dynamics of wheat bran fermentation. It offers a very interesting window into the microbial succession and plausible underlying mechanisms, which are indicative of a niche diversification of species targeting different wheat bran constituents.

## Supplementary information

Supplementary information revised version
