# Supplementary material for: Microbial succession during wheat bran fermentation and colonisation by human faecal microbiota as a result of niche diversification
Source: ISME J. 2019 Nov 11;14(2):584–96. doi: 10.1038/s41396-019-0550-5 (PMC6976558; doi:10.1038/s41396-019-0550-5)
Supplement: Supplementary file 1 — Supplementary information revised version [file 41396_2019_550_MOESM1_ESM.docx]

**Supplementary information:**

**Microbial succession during wheat bran fermentation and colonisation by human faecal microbiota as a result of niche diversification**

Kim De Paepe ^a^, Joran Verspreet ^b^, Christophe M. Courtin ^b^ and Tom Van de Wiele ^a#^

Center for Microbial Ecology and Technology (CMET), Department of Biotechnology, Faculty of Bioscience Engineering, Ghent University, Ghent, Belgium ^a^

Laboratory of Food Chemistry and Biochemistry, Leuven Food Science and Nutrition Research Centre (LFoRCe), Faculty of Bioscience Engineering, KU Leuven, Heverlee, Belgium ^b^

#corresponding author: [Tom.VandeWiele@UGent.be](mailto:Tom.VandeWiele@UGent.be)

**Supplementary materials and methods**

**Table S1: Wheat bran characterisation**

|  | **µm** |
| --- | --- |
| Average particle size | 1532 ± 149 |
|  | **(% DM)** |
| Total dietary fibre | 49.50 |
| Total arabinoxylan | 28.83 ± 0.98 |
| Arabinose/Xylose (A/X) ratio | 0.59 ± 0.09 |
| β-glucan | 2.47 ± 0.03 |
| Fructan | 3.10 ± 0.20 |
| Starch | 18.63 ± 1.00 |
| Protein | 16.86 ± 0.11 |
| Ash | 6.62 ± 0.02 |
| Lipids | 2.66 ± 1.08 |
|  | **g g^-1^ dry bran** |
| Total Water Retention Capacity | 4.22 ± 0.23 |
| Strongly bound water | 0.57 ± 0.077 |

**Bioinformatics analysis of Illumina next generation 16S rRNA gene amplicon sequencing data using mothur**

Forward and reverse reads were assembled into contigs by means of a heuristic approach based on the Phred quality scores and contigs with any ambiguous base calls or unsatisfying overlap were removed. The remaining contigs, with a length between 402 and 427 bases, were aligned to the mothur formatted silva_seed release 123 alignment database trimmed between positions 6428 and 23440, compatible with the 341F-785R primers (Quast et al 2013). Any sequences not aligning within this region or containing homopolymer stretches of more than 12 bases were removed. The remaining sequences were preclustered allowing up to 4 differences between sequences and UCHIME was applied to perform a chimera check (Edgar et al 2011). Subsequently, sequences were classified by means of a naive Bayesian classifier against the RDP 16S rRNA gene training set, version 16 with an 80% cut-off for the pseudobootstrap confidence score. Sequences annotated as *Chloroplast, Mitochondria, unknown, Archaea* or *Eukaryota* at the kingdom level were excluded. Additionally, the sequences from the mock community samples were omitted and separately analysed, resulting in a number of 16-10-16 spurious OTUs (Figure S18). Sequences were binned into Operational Taxonomic Units (OTUs) within each order, identified by the preceding classification step. The OptiClust clustering algorithm was used with a 0.15 cut-off (Westcott and Schloss 2017). Finally, sequences were binned into OTUs at a 3% dissimilarity level to generate the shared file, containing the raw sequence count data. Accordingly, an OTU is defined in this manuscript as a collection of sequences with a length between 402 and 427 nucleotides that are found to be more than 97% similar to one another in the V3-V4 region of their 16S rRNA gene after applying OptiClust clustering (Chen et al 2013, Schloss et al 2009, Schloss and Westcott 2011, Wang et al 2012). Taxonomy was assigned using the RDP version 16 and silva.nr_v123 database (Cole et al 2014, Quast et al 2013, Wang et al 2007).

**Table S2: RDP Seqmatch and NCBI BLAST results for the most abundant species retrieved after wheat bran fermentation and colonisation by the faecal material of three individuals as determined by amplicon sequencing.** The similarity score (Sab), as calculated by RDP, and the NCBI BLAST output for the best hit and next best hit(s) is shown. The NCBI maximal score (not shown) equalled the total score for all displayed hits.

|  |  | **RDP** | **NCBI BLAST** | | | |
| --- | --- | --- | --- | --- | --- | --- |
|  |  | Sab | Total score | Query coverage (%) | E-value | Identity (%) |
| OTU1 | *Escherichia fergusonii* | 1 | 787 | 100 | 0.0 | 100 |
|  | *Shigella sonei* | 1 | 787 | 100 | 0.0 | 100 |
|  | *Shigella flexneri* | 1 | 787 | 100 | 0.0 | 100 |
|  | *Escherichia coli* | 0.983 | 787 | 100 | 0.0 | 100 |
| OTU2 | *Bacteroides dorei* | 1 | 778 | 100 | 0.0 | 100 |
|  | *Bacteroides vulgatus* | 0.953 | 756 | 100 | 0.0 | 99 |
| OTU3 | *Bacteroides uniformis* | 1 | 778 | 100 | 0.0 | 100 |
|  | *Bacteroides rodentium* | 0.906 | 717 | 100 | 0.0 | 97 |
| OTU4 | *Bacteroides stercoris* | 0.981 | 778 | 100 | 0.0 | 100 |
|  | *Bacteroides intestinalis* | 0.893 | 717 | 100 | 0.0 | 97 |
| OTU5 | *Fusobacterium mortiferum* | 0.995 | 747 | 100 | 0.0 | 100 |
|  | *Fusobacterium necrogenes* | 0.964 |  |  |  |  |
| OTU6 | *Bacteroides ovatus* | 0.978 | 773 | 100 | 0.0 | 99 |
|  | *Bacteroides xylanisolvens* | 0.891 | 730 | 100 | 0.0 | 98 |
| OTU7 | *Clostridium xylanolyticum* | 0.857 | 686 | 100 | 0.0 | 98 |
|  | *Clostridium aerotolerans* | 0.844 | 678 | 100 | 0.0 | 97 |
| OTU8 | *Prevotella copri* | 0.93 | 752 | 100 | 0.0 | 99 |
|  | *Prevotella histicola* | 0.69 | 634 | 100 | 0.0 | 94 |
| OTU9 | *Prevotella copri* | 0.969 | 773 | 100 | 0.0 | 99 |
|  | *Prevotella histicola* | 0.707 | 628 | 100 | 5.0e-180 | 94 |
| OTU10 | *Eubacterium rectale* | 0.949 | 741 | 100 | 0.0 | 100 |
|  | *Roseburia faecis* | 0.941 | 713 | 100 | 0.0 | 99 |
| OTU11 | *Faecalibacterium prausnitzii* | 0.983 | 736 | 100 | 0.0 | 99 |
|  | *Gemmiger formicilis* | 0.716 | 569 | 100 | 3.0e-162 | 92 |
| OTU12 | *Oscillibacter ruminantium* | 0.732 | 612 | 100 | 5.0e-175 | 94 |
|  | *Oscillibacter valericigenes* | 0.742 | 601 | 100 | 1.0e-171 | 94 |
| OTU13 | *Sutterella wadsworthensis* | 1 | 787 | 100 | 0.0 | 100 |
|  | *Sutterella stercoricanis* | 0.793 | 654 | 100 | 0.0 | 94 |
| OTU14 | *Bacteroides eggerthii* | 0.983 | 773 | 100 | 0.0 | 99 |
|  | *Bacteroides rodentium* | 0.843 | 706 | 100 | 0.0 | 97 |
| OTU15 | *Pantoea vagans* | 1 | 787 | 100 | 0.0 | 100 |
|  | *Pantoea brenneri* | 0.952 | 784 | 100 | 0.0 | 99 |
| OTU16 | *Bacteroides caccae* | 0.966 | 767 | 100 | 0.0 | 99 |
|  | *Bacteroides faecis* | 0.865 | 723 | 100 | 0.0 | 98 |
| OTU17 | *Coprococcus eutactus* | 0.982 | 736 | 100 | 0.0 | 99 |
|  | *Eubacterium ruminantium* | 0.798 | 641 | 100 | 0.0 | 96 |
| OTU18 | *Roseburia faecis* | 1 | 743 | 100 | 0.0 | 100 |
|  | *Roseburia intestinalis* | 0.949 | 721 | 100 | 0.0 | 99 |
| OTU19 | *Clostridium xylanolyticum* | 0.863 | 654 | 100 | 0.0 | 96 |
|  | *Clostridium celerecrescens* | 0.863 | 654 | 100 | 0.0 | 96 |
|  | *Clostridium asparagiforme* | 0.86 | 654 | 100 | 0.0 | 96 |
|  | *Hungatella hathewayi* | 0.85 | 665 | 100 | 0.0 | 97 |
| OTU20 | *Bifidobacterium faecale* |  | 758 | 100 | 0.0 | 100 |
|  | *Bifidobacterium adolescentis* | 1 | 758 | 100 | 0.0 | 100 |
|  | *Bifidobacterium ruminantium* | 0.982 | 752 | 100 | 0.0 | 99 |
| OTU21 | *Faecalibacterium prausnitzii* | 0.946 | 725 | 100 | 0.0 | 99 |
|  | *Gemmiger formicilis* | 0.732 | 580 | 1 | 1.0e-165 | 93 |
|  | *Acetivibrio ethanolgignens* | 0.862 | 658 | 100 | 0.0 | 96 |
|  | *Clostridium xylanolyticum* | 0.849 | 669 | 100 | 0.0 | 97 |
| OTU22 | *Ruminococcus lactaris* |  | 671 | 100 | 0.0 | 97 |
|  | *Lachnoclostridium pacaense* |  | 669 | 100 | 0.0 | 97 |
|  | *Bacteroides xylanolyticus* |  | 669 | 100 | 0.0 | 97 |
|  | *Hungatella effluvii* |  | 669 | 100 | 0.0 | 97 |
|  | *Clostridium xylanolyticum* | 0.849 | 669 | 100 | 0.0 | 97 |
|  | *Clostridium hathewayi* | 0.823 | 669 | 100 | 0.0 | 97 |
|  | *Clostridium celerecrescens* | 0.849 | 669 | 100 | 0.0 | 97 |
|  | *Desulfotomaculum guttoideum* |  | 669 | 100 | 0.0 | 97 |
|  | *Clostridium celerecrescens* | 0.849 | 665 | 100 | 0.0 | 97 |
|  | *Clostridium sphenoides* | 0.828 | 665 | 100 | 0.0 | 97 |
|  | *Clostridium saccharolyticum* | 0.849 | 664 | 100 | 0.0 | 97 |
| OTU23 | *Bacteroides cellulosilyticus* | 0.966 | 778 | 100 | 0.0 | 100 |
|  | *Bacteroides intestinalis* | 0.934 | 756 | 100 | 0.0 | 99 |
|  | *Prevotella copri* | 0.883 | 736 | 100 | 0.0 | 98 |
|  | *Prevotella micans* | 0.704 | 590 | 100 | 2.0e-168 | 92 |
| OTU24 | *Bacteroides xylanisolvens* | 1 | 778 | 100 | 0.0 | 100 |
|  | *Bacteroides acidifaciens* | 0.959 | 756 | 100 | 0.0 | 99 |
| OTU25 | *Parabacteroides merdae* | 1 | 778 | 100 | 0.0 | 100 |
|  | *Parabacteroides johnsonii* | 0.895 | 728 | 100 | 0.0 | 98 |
| OTU26 | *Clostridium bolteae* | 0.814 | 647 | 100 | 0.0 | 96 |
|  | *Clostridium asparagiforme* | 0.804 | 636 | 100 | 0.0 | 95 |
| OTU27 | *Parabacteroides distasonis* | 0.956 | 773 | 100 | 0.0 | 99 |
|  | *Parabacteroides gordonii* | 0.674 | 610 | 100 | 2.0e-174 | 93 |
| OTU28 | *Bacteroides thetaiotaomicron* | 1 | 778 | 100 | 0.0 | 100 |
|  | *Bacteroides faecichinchillae* | 0.947 | 756 | 100 | 0.0 | 99 |
| OTU29 | *Veillonella dispar* | 0.968 | 776 | 100 | 0.0 | 99 |
|  | *Veillonella tobetsuensis* | 0.949 | 760 | 100 | 0.0 | 99 |
| OTU30 | *Flavonifractor plautii* | 0.683 | 534 | 100 | 1.0e-151 | 91 |
|  | *Pseudoflavonifractor capillosus* | 0.652 | 551 | 100 | 1.0e-156 | 91 |
|  | *Oscillibacter ruminantium* |  | 573 | 100 | 2.0e-163 | 92 |
| OTU31 | *Oscillibacter ruminantium* | 0.798 | 651 | 100 | 0.0 | 96 |
|  | *Oscillibacter valericigenes* | 0.79 | 617 | 100 | 1.0e-176 | 94 |
|  | *Oscillibacter valericigenes* | 0.79 | 617 | 100 | 1.0e-176 | 94 |
|  | *Flavonifractor plautii strain* | 0.645 | 556 | 100 | 2.0e-158 | 92 |
|  | *Intestinimonas butyriciproducens* | 0.655 | 484 | 100 | 1.0e-136 | 88 |
| OTU33 | *Eubacterium eligens* | 0.912 | 736 | 100 | 0.0 | 99 |
|  | *Lactobacillus rogosae* |  | 675 | 100 | 0.0 | 97 |
|  | *Lachnospira multipara* | 0.82 | 658 | 100 | 0.0 | 96 |
|  | *Lachnospira pectinoschiza* |  | 643 | 100 | 0.0 | 95 |
| OTU34 | *Akkermansia muciniphila* | 1 | 752 | 100 | 0.0 | 100 |
| OTU40 | *Phascolarctobacterium succinatutens* | 0.859 | 715 | 100 | 0.0 | 97 |
|  | *Phascolarctobacterium faecium* | 0.732 | 632 | 100 | 0.0 | 93 |
| OTU45 | *Phascolarctobacterium succinatutens* | 1 | 787 | 100 | 0.0 | 100 |
|  | *Phascolarctobacterium faecium* | 0.72 | 632 | 100 | 0.0 | 93 |
| OTU46 | *Clostridium bolteae* | 1 | 741 | 100 | 0.0 | 100 |
|  | *Clostridium clostridioforme* | 0.977 | 741 | 100 | 0.0 | 100 |
| OTU48 | *Bifidobacterium longum strain* | 0.985 | 750 | 100 | 0.0 | 100 |
|  | *Bifidobacterium ramosum* |  | 706 | 100 | 0.0 | 98 |
|  | *Bifidobacterium pullorum* | 0.851 | 701 | 100 | 0.0 | 98 |
|  | *Bifidobacterium saguini* | 0.877 | 697 | 100 | 0.0 | 98 |
|  | *Bifidobacterium myosotis* |  | 695 | 100 | 0.0 | 98 |
|  | *Bifidobacterium breve* | 0.918 | 671 | 100 | 0 | 96 |
| OTU50 | *Lachnoclostridium pacaense* |  | 675 | 100 | 0.0 | 97 |
|  | *Clostridium xylanolyticum* | 0.869 | 669 | 100 | 0.0 | 97 |
|  | *Clostridium hathewayi* | 0.851 | 669 | 100 | 0.0 | 97 |
|  | *Hungatella effluvii* |  | 664 | 100 | 0.0 | 97 |
|  | *Clostridium celerecrescens* | 0.866 | 664 | 100 | 0.0 | 97 |
|  | *Clostridium amygdalinum* | 0.838 | 664 | 100 | 0.0 | 97 |
|  | *Clostridium asparagiforme* | 0.879 | 664 | 100 | 0.0 | 97 |
|  | *Desulfotomaculum guttoideum* |  | 664 | 100 | 0.0 | 97 |
|  | *Clostridium aerotolerans* | 0.866 | 662 | 100 | 0.0 | 97 |
| OTU53 | *Eubacterium siraeum* | 0.954 | 726 | 100 | 0.0 | 99 |
|  | *Acetanaerobacterium elongatum* |  | 496 | 100 | 5.0e-140 | 98 |
| OTU54 | *Clostridium asparagiforme* | 0.902 | 680 | 100 | 0.0 | 97 |
|  | *Clostridium lavalense* | 0.887 | 675 | 100 | 0.0 | 97 |
|  | *Robinsoniella peoriensis* | 0.876 | 675 | 100 | 0.0 | 97 |
| OTU63 | *Prevotella copri* | 0.881 | 725 | 100 | 0.0 | 98 |
|  | *Prevotella micans* | 0.703 | 584 | 100 | 1.0e-166 | 92 |
|  | *Prevotella oulorum* | 0.701 | 623 | 100 | 2.0e-178 | 93 |
|  | *Prevotella albensis* | 0.701 | 606 | 100 | 2.0e-173 | 93 |
| OTU69 | *Citrobacter braakii* | 0.974 | 787 | 100 | 0.0 | 100 |
|  | *Citrobacter werkmanii* | 0.983 | 782 | 100 | 0.0 | 99 |
|  | *Citrobacter freundii* | 0.978 | 776 | 100 | 0.0 | 99 |
| OTU71 | *Enterococcus hirae* | 1 | 787 | 100 | 0.0 | 100 |
|  | *Enterococcus villorum* | 1 | 787 | 100 | 0.0 | 100 |
|  | *Enterococcus ratti* | 1 | 787 | 100 | 0.0 | 100 |
|  | *Enterococcus durans* | 1 | 787 | 100 | 0.0 | 100 |
|  | *Enterococcus faecium* | 0.988 | 787 | 100 | 0.0 | 100 |
|  | *Enterococcus lactis* | 0.986 | 782 | 100 | 0.0 | 99 |
|  | *Enterococcus rivorum* | 0.983 | 784 | 100 | 0.0 | 99 |
|  | *Enterococcus thailandicus* | 0.983 | 782 | 100 | 0.0 | 99 |
| OTU83 | *Butyrivibrio crossotus* | 1 | 741 | 100 | 0.0 | 100 |
|  | *Clostridium hathewayi* | 0.826 | 647 | 100 | 0.0 | 96 |
| OTU123 | *Clostridium symbiosum* | 0.933 | 719 | 100 | 0.0 | 99 |
|  | *Clostridium clostridioforme* | 0.874 | 675 | 100 | 0.0 | 97 |
|  | *Clostridium bolteae* | 0.892 | 675 | 100 | 0.0 | 97 |
|  | *Clostridium citroniae* | 0.861 | 664 | 100 | 0.0 | 97 |
| OTU148 | *Veillonella atypica* | 0.956 | 767 | 100 | 0.0 | 99 |
|  | *Veillonella dispar* | 0.932 | 737 | 100 | 0.0 | 98 |
| OTU153 | *Ruminococcus champanellensis* | 0.966 | 725 | 100 | 0.0 | 99 |
|  | *Ruminococcus flavefaciens* | 0.815 | 658 | 100 | 0.0 | 96 |
|  | *Ruminococcus callidus* | 0.807 | 647 | 100 | 0.0 | 96 |

**Statistical analysis**

All statistical analyses were performed in R, version 3.4.1 (R Core Team 2016) unless otherwise stated. All formal hypothesis tests were conducted on the 5% significance level (α = 0.05).

Differences in microbial community composition between the donors, time points and niches (luminal *versus* bran-attached) were explored using Principle Coordinate Analysis (PCoA; package stats 3.4.0) and visualised with ggplot 2.2.1 (Becker et al 1988, Cailliez 1983, Cox 2001, Gower 1966, Ramette 2007, Wickham 2009). For this purpose, the shared file (including the duplicate samples) was filtered based on the arbitrary cut-offs described by McMurdie and Holmes (2014), whereby OTUs observed in less than 5% of the samples and with read counts below 0.5 times the number of samples were removed. The data, excluding the duplicate samples (see below), was rescaled to proportions and the abundance based jaccard dissimilarity matrix was calculated (package vegan 2.4-3) (Anderson et al 2006, Borcard et al 2011, McMurdie and Holmes 2014, Oksanen et al 2016). On the genus level, weighed averages of genera abundances were *a posteriori* added to the ordination plot, using the wascores function in vegan (Oksanen et al 2016).

The time series data at phylum, genus and OTU level were further separately analysed for each donor and niche (luminal *versus* bran-attached). The analysis were performed starting from the proportional and filtered (based on the entire dataset as described above) shared file, unless stated otherwise. For each time series, the community similarity for each pair of time points was determined by calculating the abundance based jaccard dissimilarity matrix (package vegan 2.4-3) and subtracting the values from 1. The resulting similarity matrix was visualised using the heatmap.2 function (package gplots_3.0.1) (Warnes et al 2016). Columns and rows were reordered according to an UPGMA clustering dendrogram (package cluster_2.0.6) (Maechler et al 2017). To verify the quality of the clustering approach, the cluster tightness and separation were evaluated by constructing silhouette plots (cluster_2.0.6) (Rousseeuw 1987).

In order to evaluate the time decay in community similarity, the similarity values were plotted in function of the time interval between the samples and a log-linear model was fitted to the data, as described by Shade et al. (2013).

Additionally, in order to evaluate the overall community dynamics, similarity values for consecutive pairs of time points were plotted as a function of time. Similarity values below 0.6 are indicated in red. The dynamics for individual taxa was inferred by calculating the cumulative shift in proportional abundance between consecutive time points, summed over all time points, according to the following formula with A(i) t, the proportional abundance of a taxon i at time point t.

$$\sum_{time} {[A(i)}_{t}-{A\left( i \right)}_{t-1}]$$

For each donor, graphs (Figure 5-7) were constructed displaying the proportional abundance of the 10% most variable taxa over time at genus and species level. Additionally, the dynamics of the most abundant taxa is displayed in stacked area plots of the proportional abundances (Figure 4).

Co-occurrence interactions between different taxa and metadata variables were assessed using the CoNet network construction tool (version 1.1.1.beta), implemented in cytoscape version 3.5.1 (Faust and Raes 2016, Shannon et al 2003). Networks were created on the complete 16S read count dataset, as wells as on subsets by donor and by donor and niche, considering the whole time series. Acetate, propionate, butyrate and total branched SCFA (short chain fatty acids) levels, pH and time were included as metadata. For each data subset the filtered shared file was further processed to retain only entries exceeding a minimum cumulative read count of 20, at both genus and species level. After importing in CoNet, the count table was converted to relative abundances by dividing read counts by the total sample read count (“standardization col_norm” option). A suite of methods for network inference were combined, consisting of Pearson, Spearman and Kendall correlation based measures next to Bray-Curtis and Kullback-Leibler dissimilarity metrics (Faust et al 2012, Faust et al 2015). A threshold was specified to retain only the 100 most positive and negative interactions (edges) at genus level (“top and bottom” option). At species level the desired number of edges was increased to 1000. To assess the significance of the retrieved edges, method- and edge-specific p-values were obtained by applying permutation tests with row wise resampling (“EdgeScores with shuffle rows option and 1000 iterations”). The resulting permutation distribution was saved to a file. To deal with spurious correlation-based interactions due to compositionality, CoNet provides an option (ReBoot method) to perform combined renormalisation permutation and bootstrapping (Faust et al 2012, Faust and Raes 2016). In a second CoNet run edgescores, computed from the bootstrap procedure (1000 iterations) were compared to the compositionality-aware null distribution of renormalised permuted edgescores, loaded from the saved file. To allow rebuilding of the network in a later stage, the bootstrap distribution was written to a file. Finally, the resulting networks were merged preserving only significant interactions with a merged p-value below 0.05 and supported by the majority of the methods (Faust et al 2012). The merged p-value was computed by merging the method-specific p-values using the brown method adjusted for multiple testing using the Benjamini-Hochberg False Discovery Rate (FDR) correction (Faust and Raes 2016). For the purpose of visualisation the number of edges was further reduced by re-computing the network from the bootstrap and permutation files with a more stringent filtering, restricting the number of edges to 75 respectively 100 at genus respectively species level (“top and bottom” option). Networks were formatted in cytoscape to obtain an organic layout with positive and negative interactions depicted in green and red respectively. Vertices were coloured according to phylum level membership. Union networks were built in cytoscape by i) merging the networks of the three donors or ii) merging the luminal and bran-attached networks for each donor (Faust et al 2012). Prior to merging, in all these cases a column was added to the network data table containing a network identifier, to allow tracing back the interactions in the union network to specific i) donors or ii) niches. Edge line types were formatted according to the number of i) donors or ii) niches supporting a given interaction and hence are a measure of the stability of interactions over i) donors and ii) niches. Edge widths were formatted based on a continuous mapping of the merged p-values obtained by the brown method (as outlined above). The edge width decreases with increasing p-values (less significant interactions). In order to visualise the complex community structure of the networks, clustering was applied based on the eigenvector approach proposed by Newman (2006) by running the GLay community cluster algorithm from the ClusterMaker2 app (version 1.1.0) in cytoscape (Morris et al 2011, Su et al 2010). Clustering identifies so-called “communities”, which are clusters of nodes characterised by a high within-cluster node connectivity and a low between-cluster connectivity. The union networks for donor and niche were summarised in a venn diagram representing the number of shared and unique edges retrieved with a threshold of 100 and 1000 at genus and species level respectively (package VennDiagram 1.6.17) (Chen 2016).

The reproducibility of the wheat bran fermentation was assessed by analysing biological replicates. The SCFA production was analysed in duplicate for all conditions (donors and time points), whereas the 16S rRNA gene amplicon sequencing was only replicated for three time points for all donors in the luminal and bran niche. The coefficient of variation (CV) was calculated on both types of data and visualised in a box plot. Box plots of the SCFA levels display the distribution of the CV for the duplicate samples, for each donor and time point. Box plots of the 16S rRNA gene amplicon sequencing data display the distribution of the CV for all OTUs, subsetted by donor and time point. In addition for the 16S rRNA gene amplicon sequencing data, principle coordinate analysis (PCoA) and UPGMA clustering were performed, using the abundance based jaccard dissimilarity matrix, according to the methods outlined above.

**Supplementary results**

**
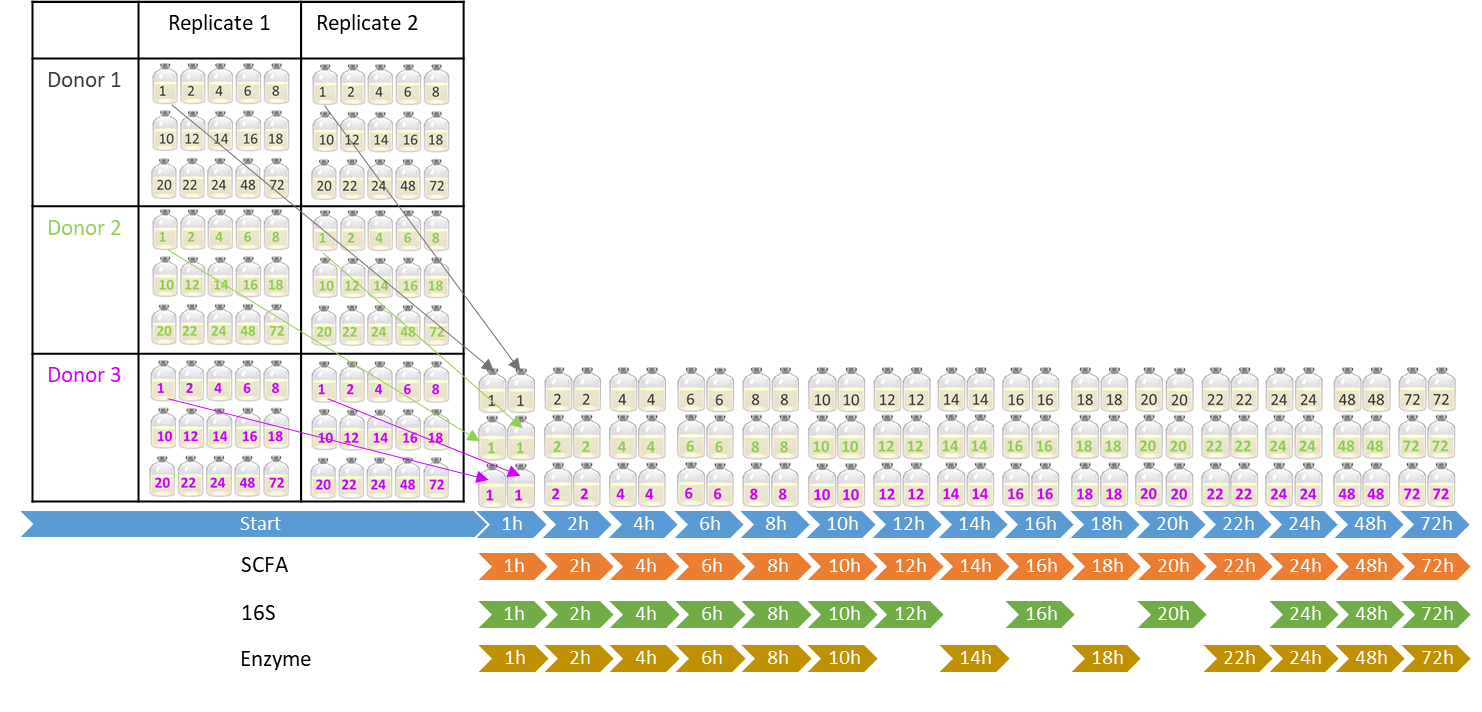
**

**Figure S1: Overview of the experimental set-up.** For each time point (1 up to 72 h) and each donor (indicated in different colours), a separate tube (further referred to as a biological replicate), containing wheat bran supplemented to a carbohydrate-low medium, was incubated in duplicate at the start of the experiment. In case of donor 2 and 3, in addition, a control consisting of the carbohydrate-low medium without wheat bran was taken along (not shown in the figure). SCFA concentrations were analysed every time point for both biological replicates (n=2). Enzyme activity measurements were performed less frequently for both biological replicates (n=2). One biological replicate was sequenced with 16S rRNA gene amplicon sequencing for most time points (n=1). From time points 2,10 and 48, two biological replicates were assessed.


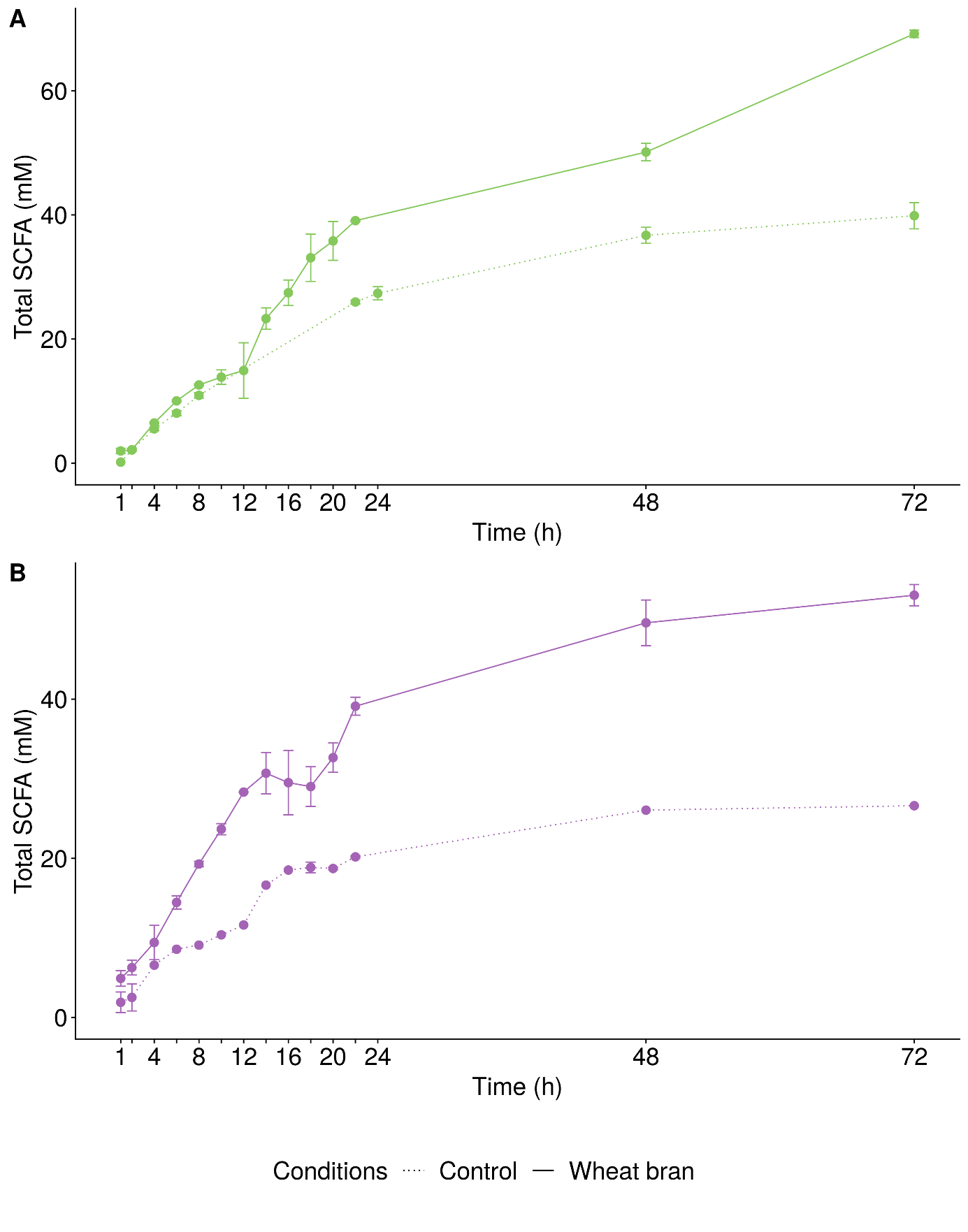


**Figure S2: SCFA production (n=2 biological replicates) in the carbohydrate-low medium (Control, dotted line) compared to the wheat bran supplemented carbohydrate-low medium (Wheat bran, solid line) incubated with the faecal material of donor 2 (panel A) and 3 (panel B) for up to 72 h.**


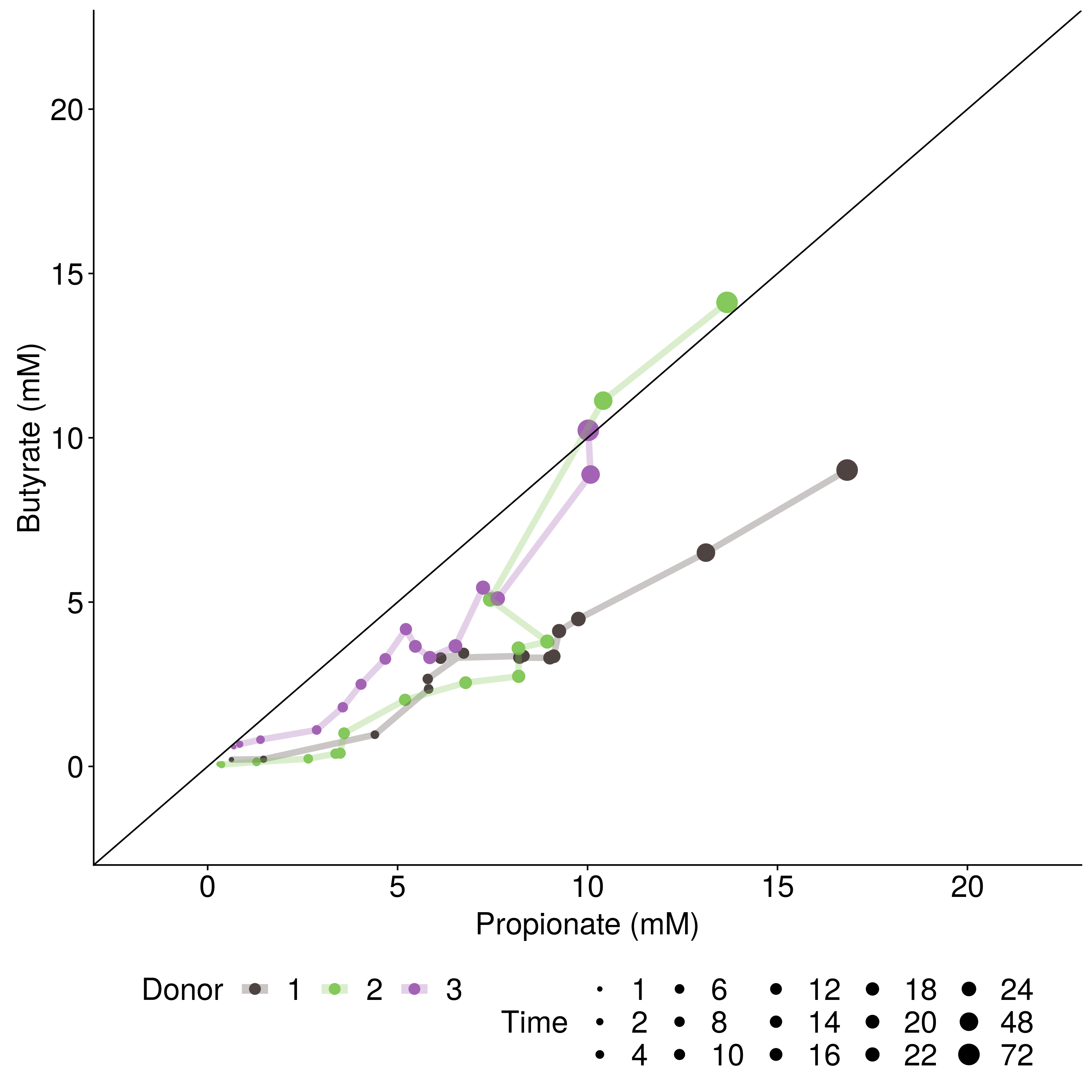


**Figure S3: Propionate and butyrate production (n=2 biological replicates) resulting from the incubation of wheat bran in the presence of a carbohydrate-low medium with the faecal material of three different donors for up to 72 h. Lines connect the consecutive time points.**


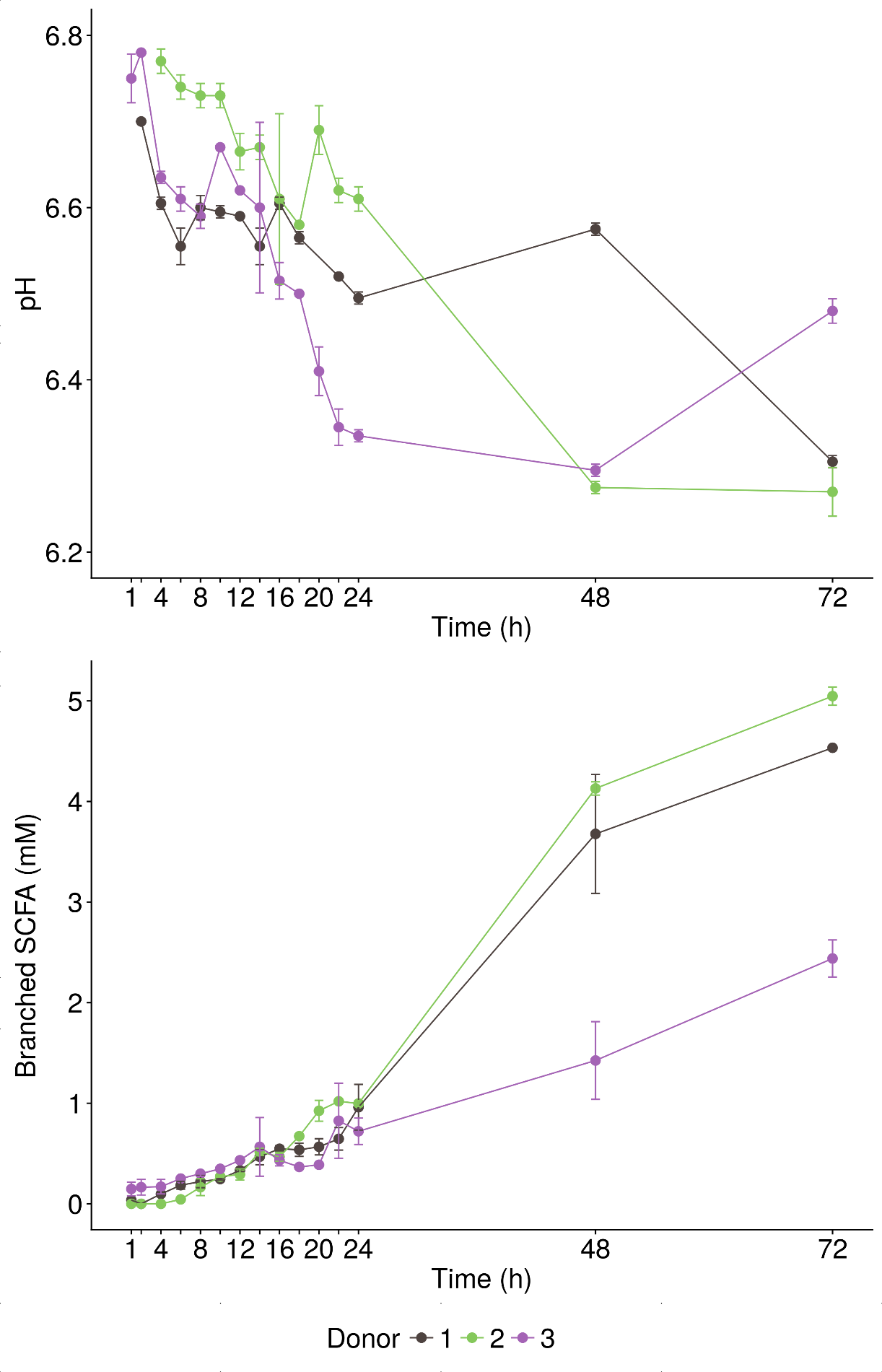


**Figure S4: Branched SCFA production and pH drop (n=2 biological replicates) resulting from the incubation of wheat bran** **in the presence of a carbohydrate-low medium with the faecal material of three different donors for up to 72 h.**


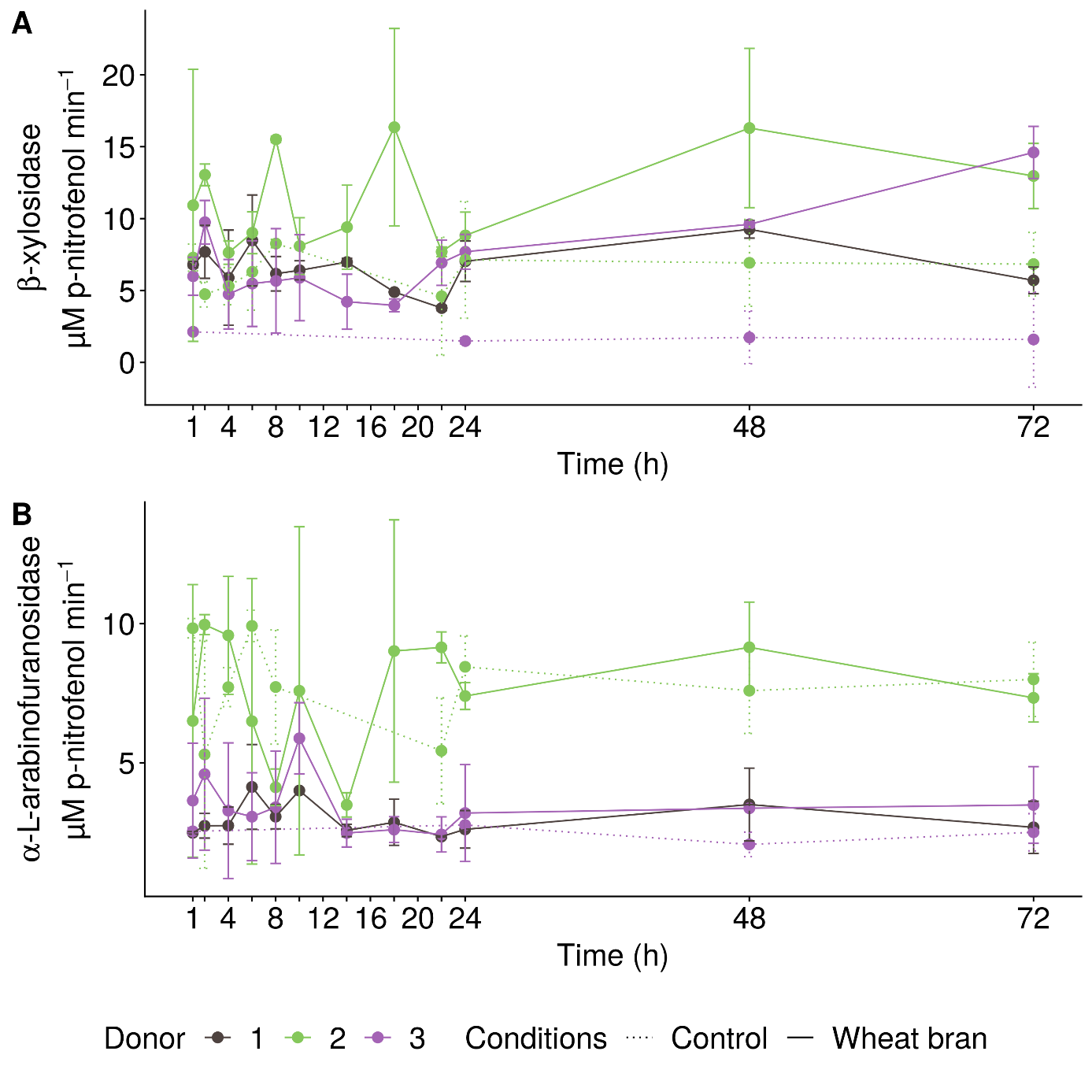


**Figure S5: β-xylosidase (A) and α-L-arabinofuranosidase (B) enzyme activity (n=2 biological replicates) measured in the membrane-associated and intracellular fraction of liquid samples, consisting of a carbohydrate-low medium incubated with the faecal material of three different donors for up to 72 h in the absence (dotted line, Control) or presence (solid line, Wheat bran) of wheat bran.** Enzyme activity was expressed as the concentration of 4-nitrophenol (µM) released from the 4-nitrophenyl-β-D-xylopyranoside (A) and 4-nitrophenyl-α-L-arabinofuranoside (B) artificial substrates per minute at pH 7, 37°C.


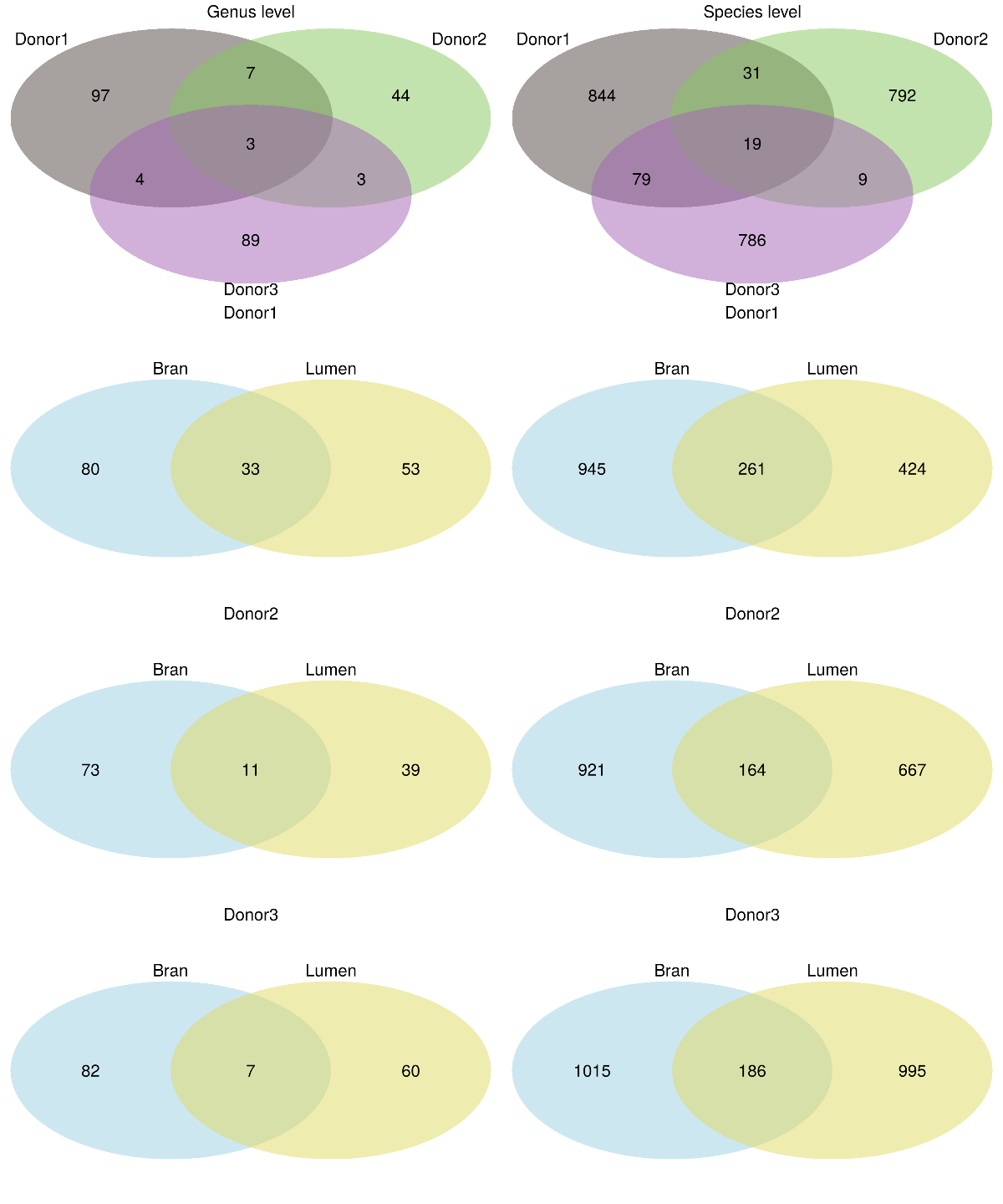


**Figure S6: Venn diagram of the stable co-occurrence interactions (edges) at genus and species level, shared between the three donors and between the luminal and bran-attached microbial community for each donor (n=1).**


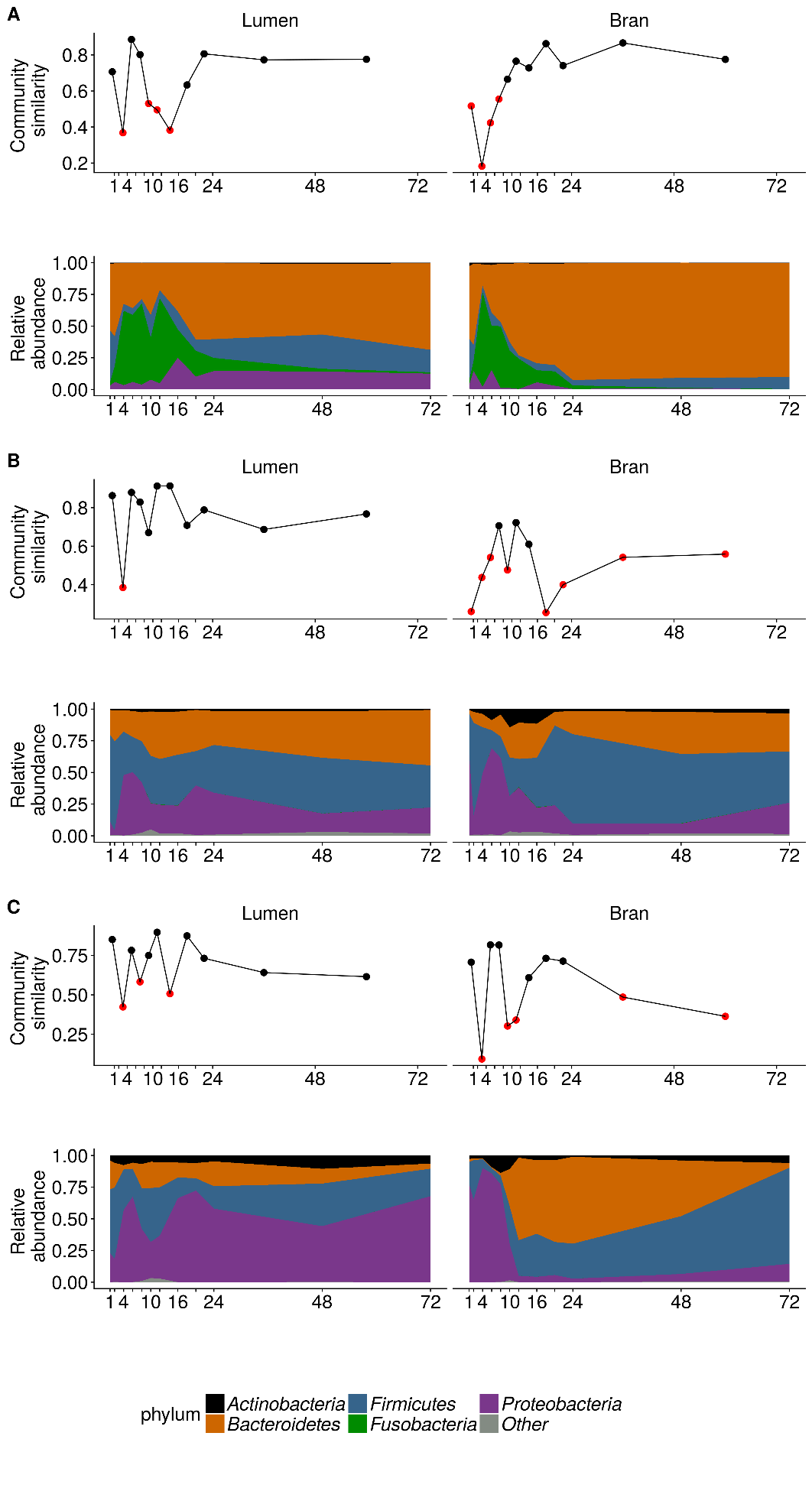


**Figure S7: Phylum level luminal and bran-attached community composition and community similarity between consecutive time points of donor 1 (A), donor 2 (B) and donor 3 (C) (n=1). The similarity value was calculated by subtracting the abundance based jaccard dissimilarity metric from 1. Similarity values below 0.6 are indicated in red.**


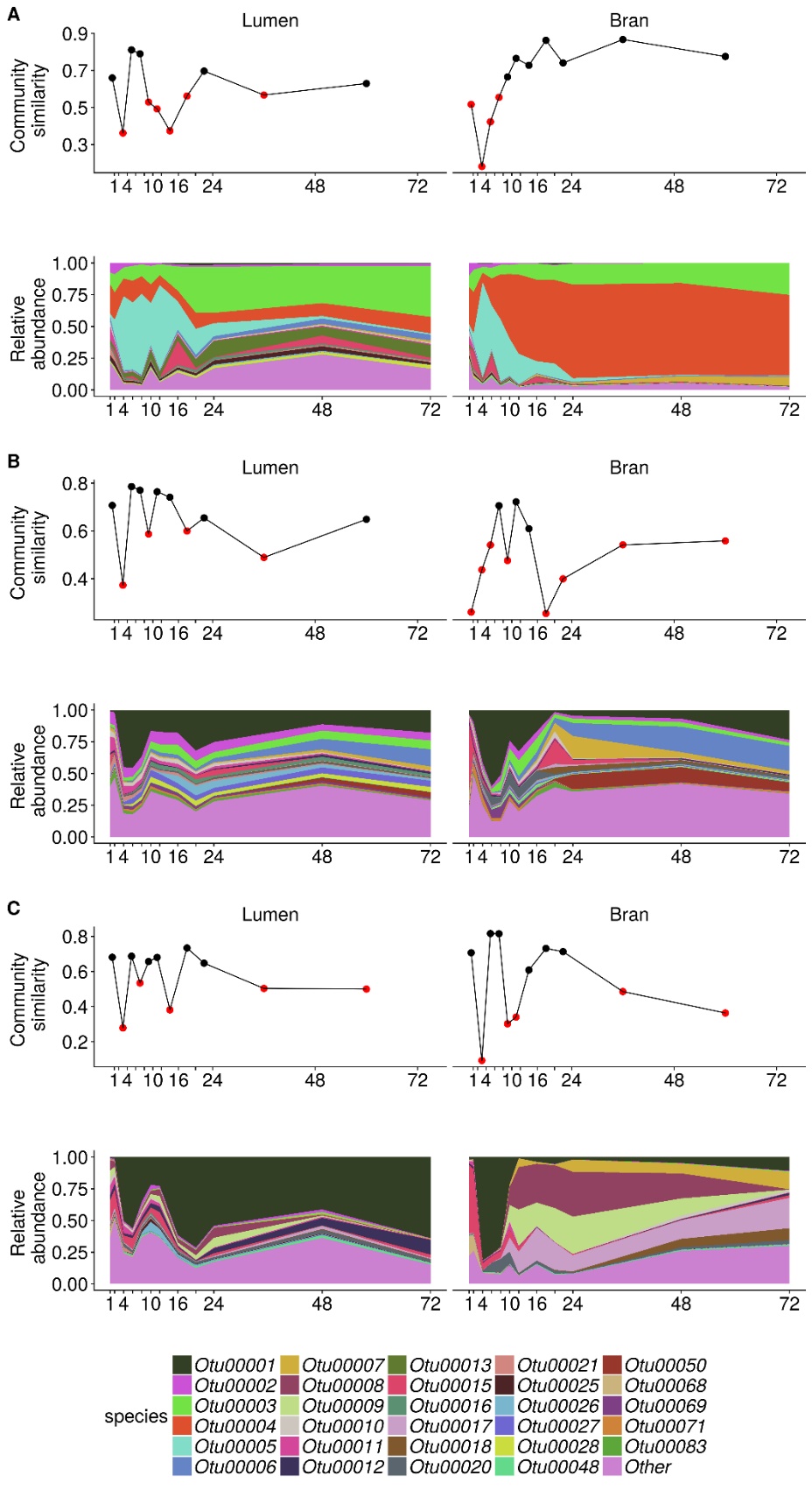


**Figure S8: Species level luminal and bran-attached community composition and community similarity between consecutive time points of donor 1 (A), donor 2 (B) and donor 3 (C) (n=1). The similarity value was calculated by subtracting the abundance based jaccard dissimilarity metric from 1. Similarity values below 0.6 are indicated in red.**


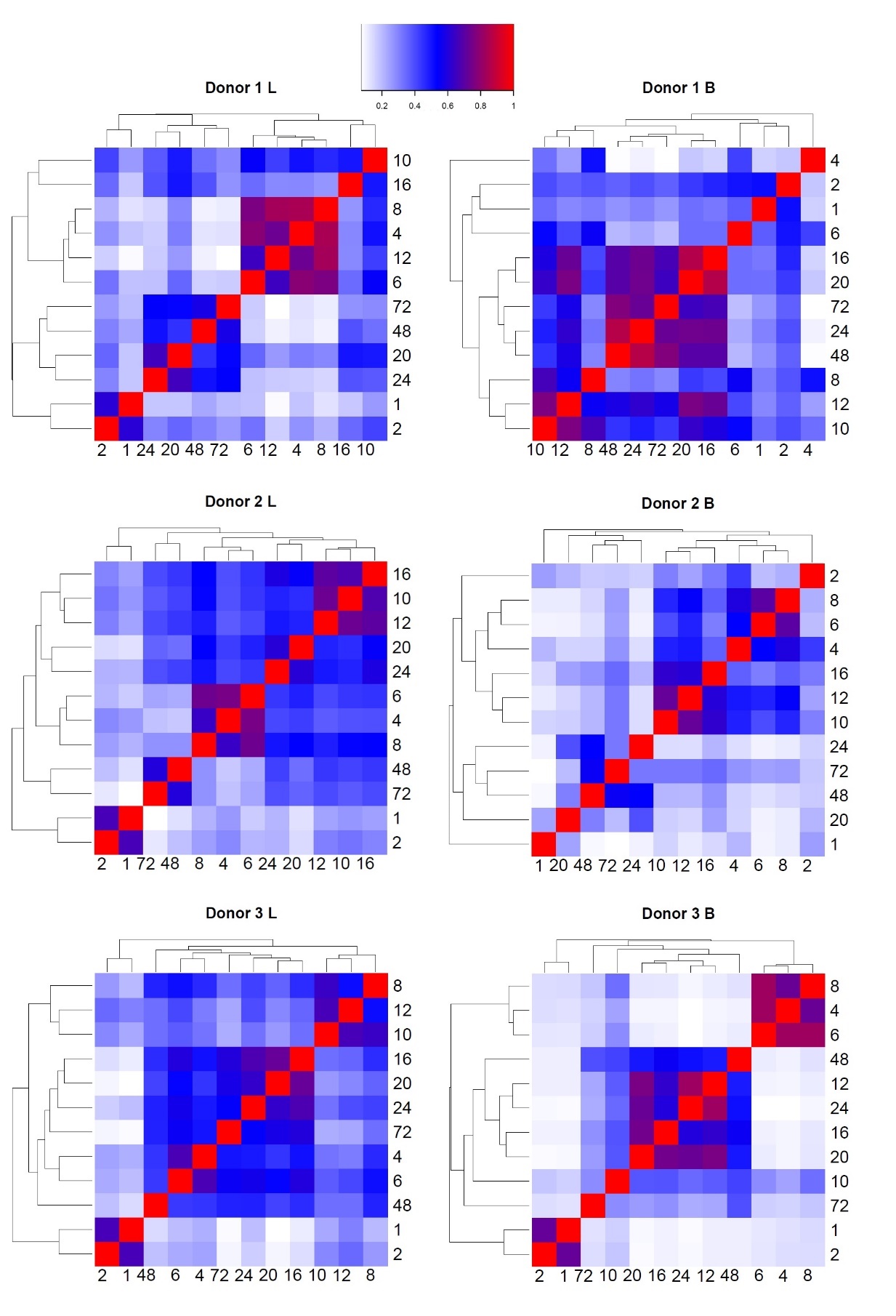


**Figure S9: Heatmap displaying the similarity between the luminal (L) and bran-attached (B) microbial community composition at different time points (1,2,4,6,8,10,12,16,20,24,48 and 72 h) (n=1).**


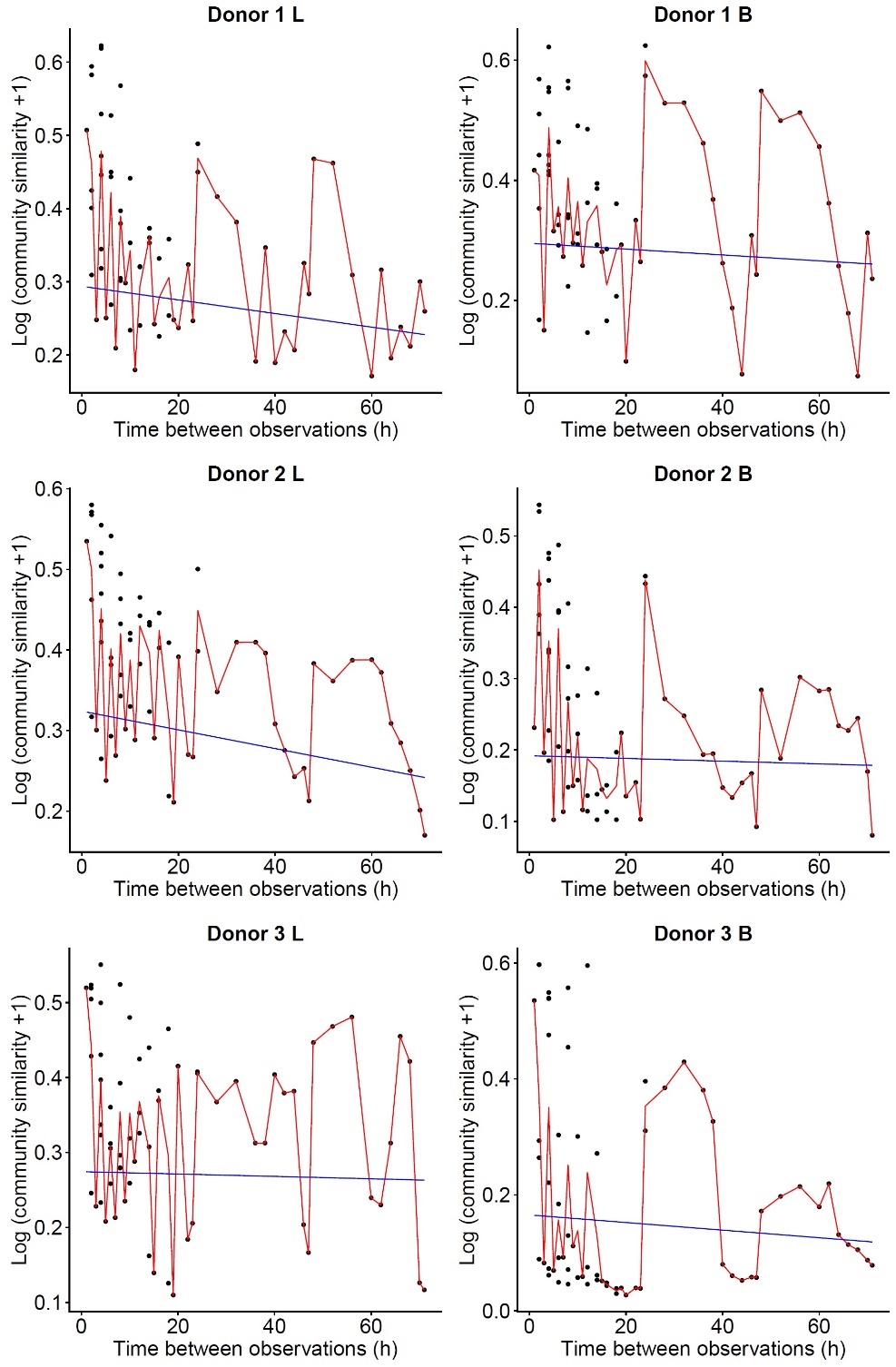


**Figure S10: Time decay analysis of the luminal (L) and bran-attached (B) microbial community composition (n=1).** The red line connects the average community similarity in each time interval and the blue line indicates the log-linear time decay model fit. Community similarity is not a function of the length of the time interval and for a certain time span, e.g. for a difference of 2 hours between sampling time points, a large variation in community similarity (large spread of values) occurred, indicating that the community did not change at the same rate in all two hour intervals and that the observed dynamics are not consequence of uneven sampling.


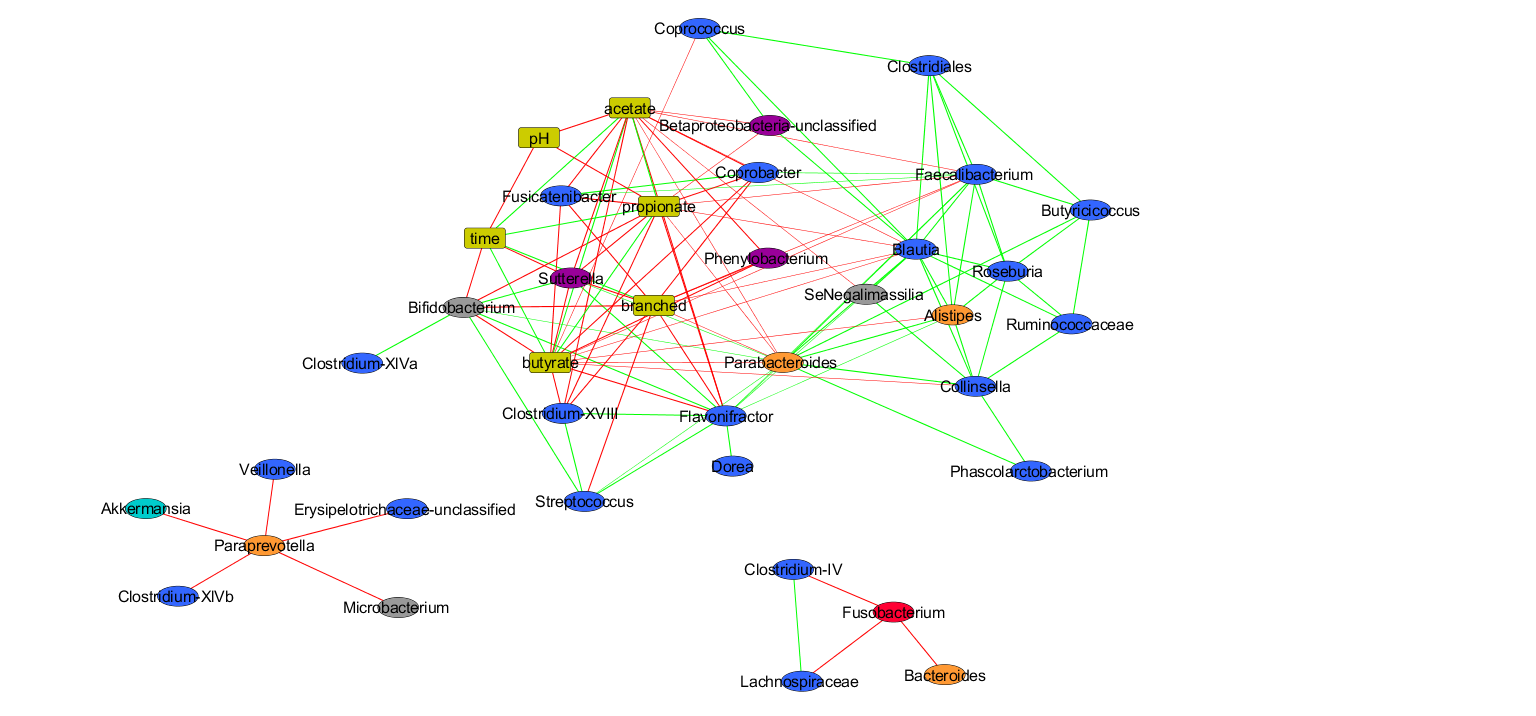


**Figure S11: Co-occurrence network of the luminal microbial community composition of donor 1 (n=1)**. Metadata variables are indicated by a yellow fill colour and taxa are coloured by phylum level classification. Edge line widths are determined by a continuous mapping of the merged p-value obtained by the brown method. A decreasing line width corresponds to an increasing p-value (less significant interaction).


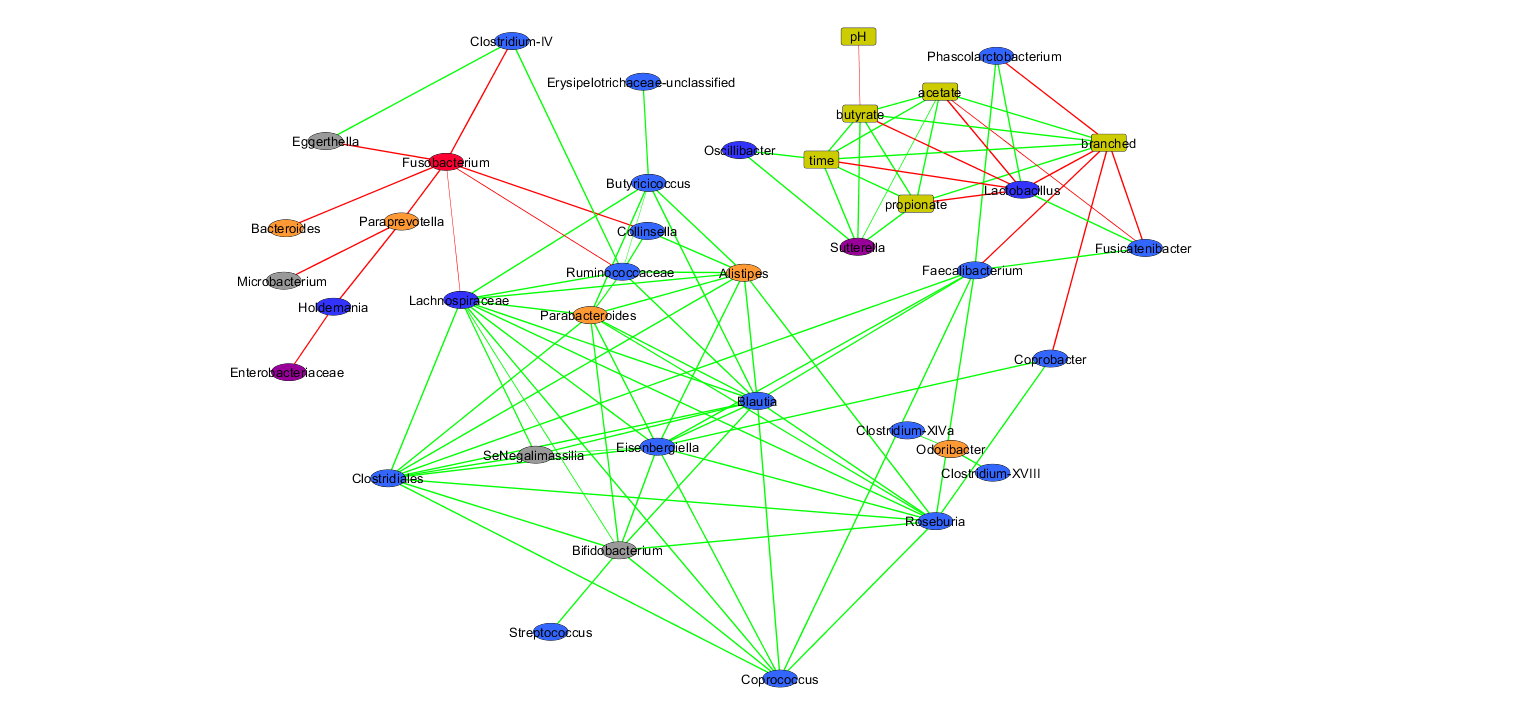


**Figure S12:**  **Co-occurrence network of the bran-attached microbial community composition of donor 1 (n=1)**. Metadata variables are indicated by a yellow fill colour and taxa are coloured by phylum level classification. Edge line widths are determined by a continuous mapping of the merged p-value obtained by the brown method. A decreasing line width corresponds to an increasing p-value (less significant interaction).


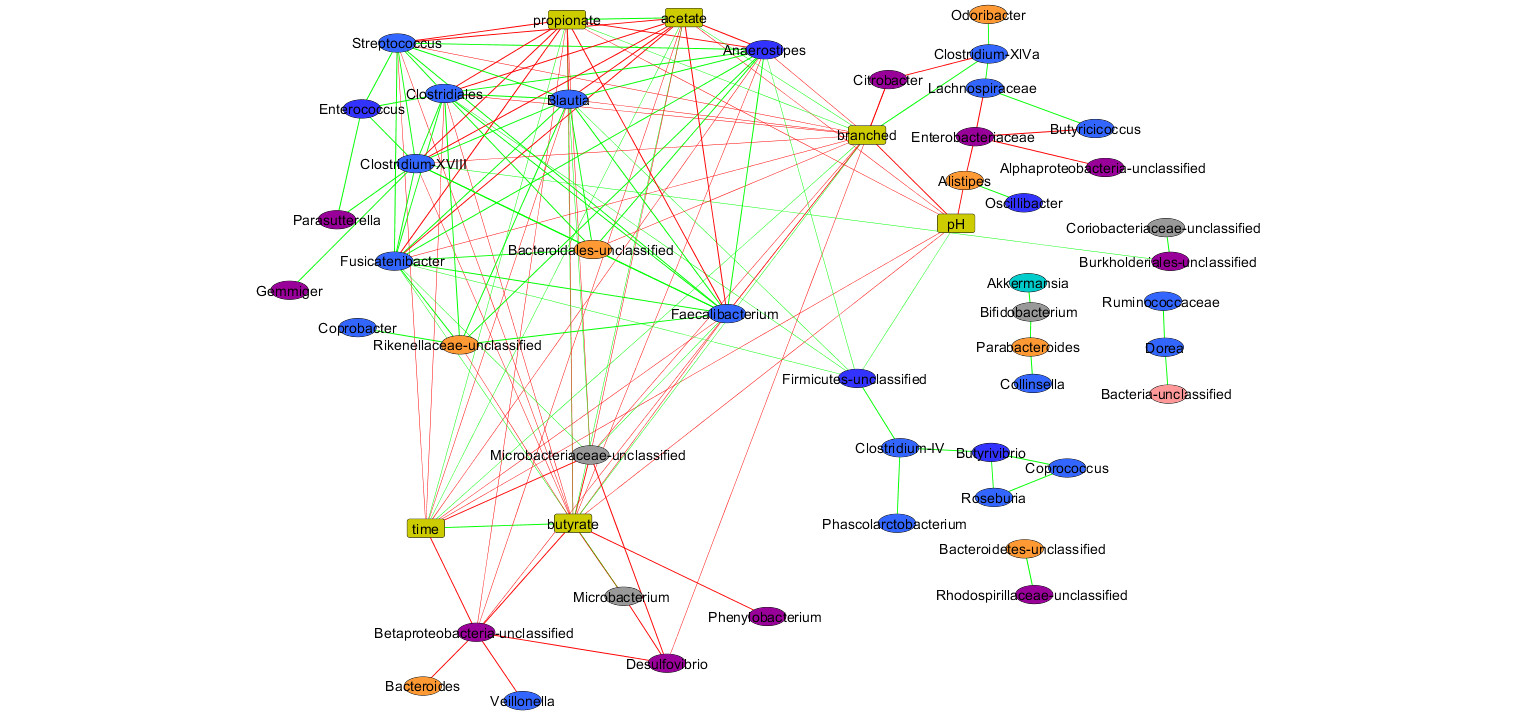


**Figure S13: Co-occurrence network of the luminal microbial community composition of donor 2 (n=1)**. Metadata variables are indicated by a yellow fill colour and taxa are coloured by phylum level classification. Edge line widths are determined by a continuous mapping of the merged p-value obtained by the brown method. A decreasing line width corresponds to an increasing p-value (less significant interaction).


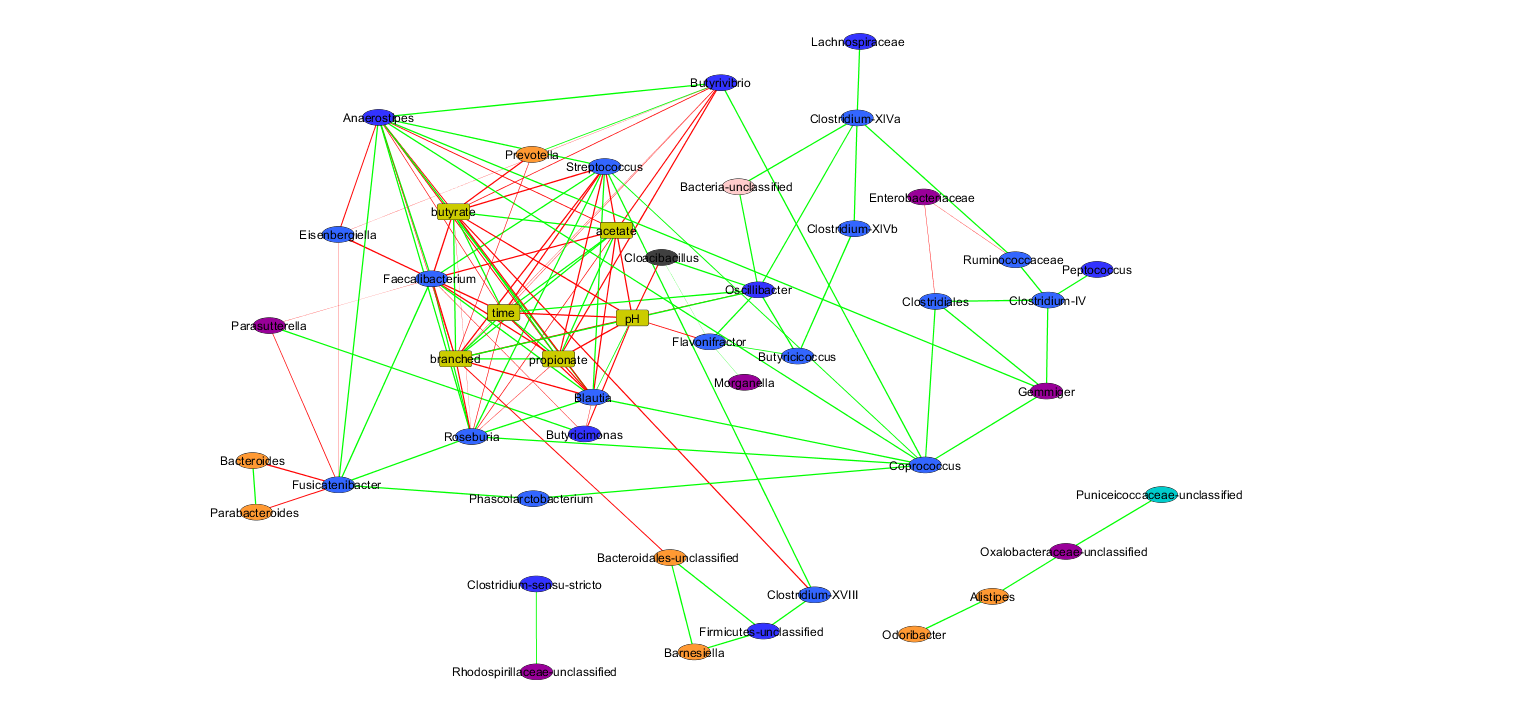


**Figure S14: Co-occurrence network of the bran-attached microbial community composition of donor 2 (n=1)**. Metadata variables are indicated by a yellow fill colour and taxa are coloured by phylum level classification. Edge line widths are determined by a continuous mapping of the merged p-value obtained by the brown method. A decreasing line width corresponds to an increasing p-value (less significant interaction).


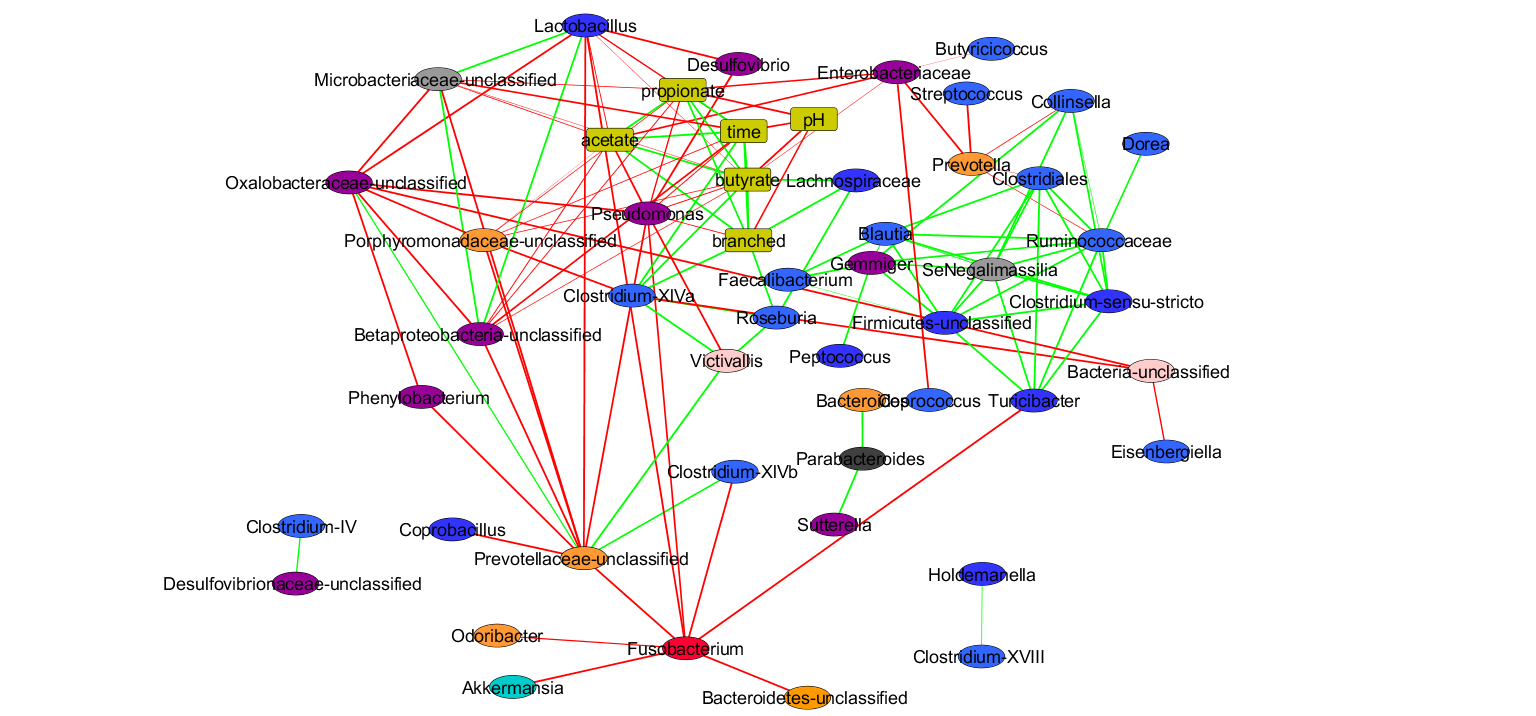


**Figure S15: Co-occurrence network of the luminal microbial community composition of donor 3 (n=1)**. Metadata variables are indicated by a yellow fill colour and taxa are coloured by phylum level classification. Edge line widths are determined by a continuous mapping of the merged p-value obtained by the brown method. A decreasing line width corresponds to an increasing p-value (less significant interaction).


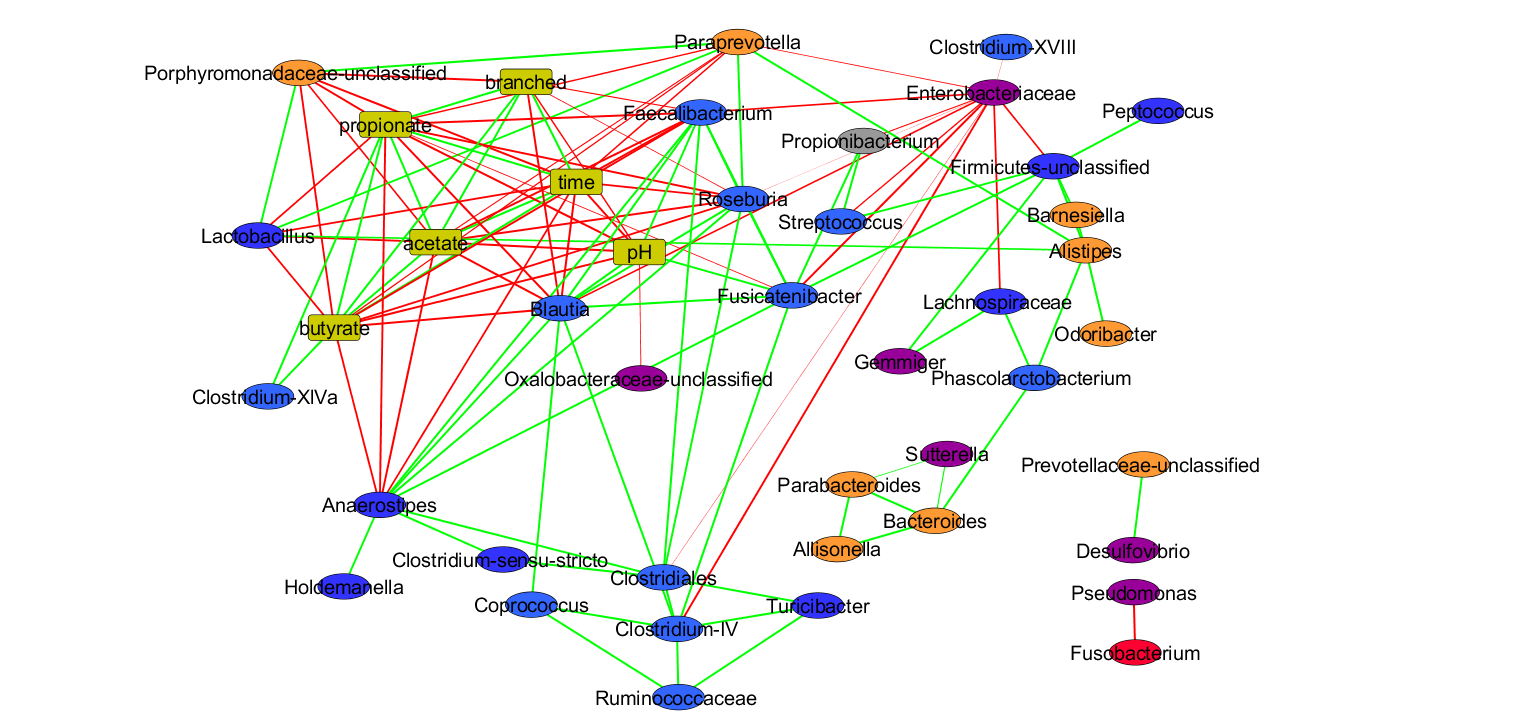


**Figure S16: Co-occurrence network of the bran-attached microbial community composition of donor 3 (n=1)**. Metadata variables are indicated by a yellow fill colour and taxa are coloured by phylum level classification. Edge line widths are determined by a continuous mapping of the merged p-value obtained by the brown method. A decreasing line width corresponds to an increasing p-value (less significant interaction).


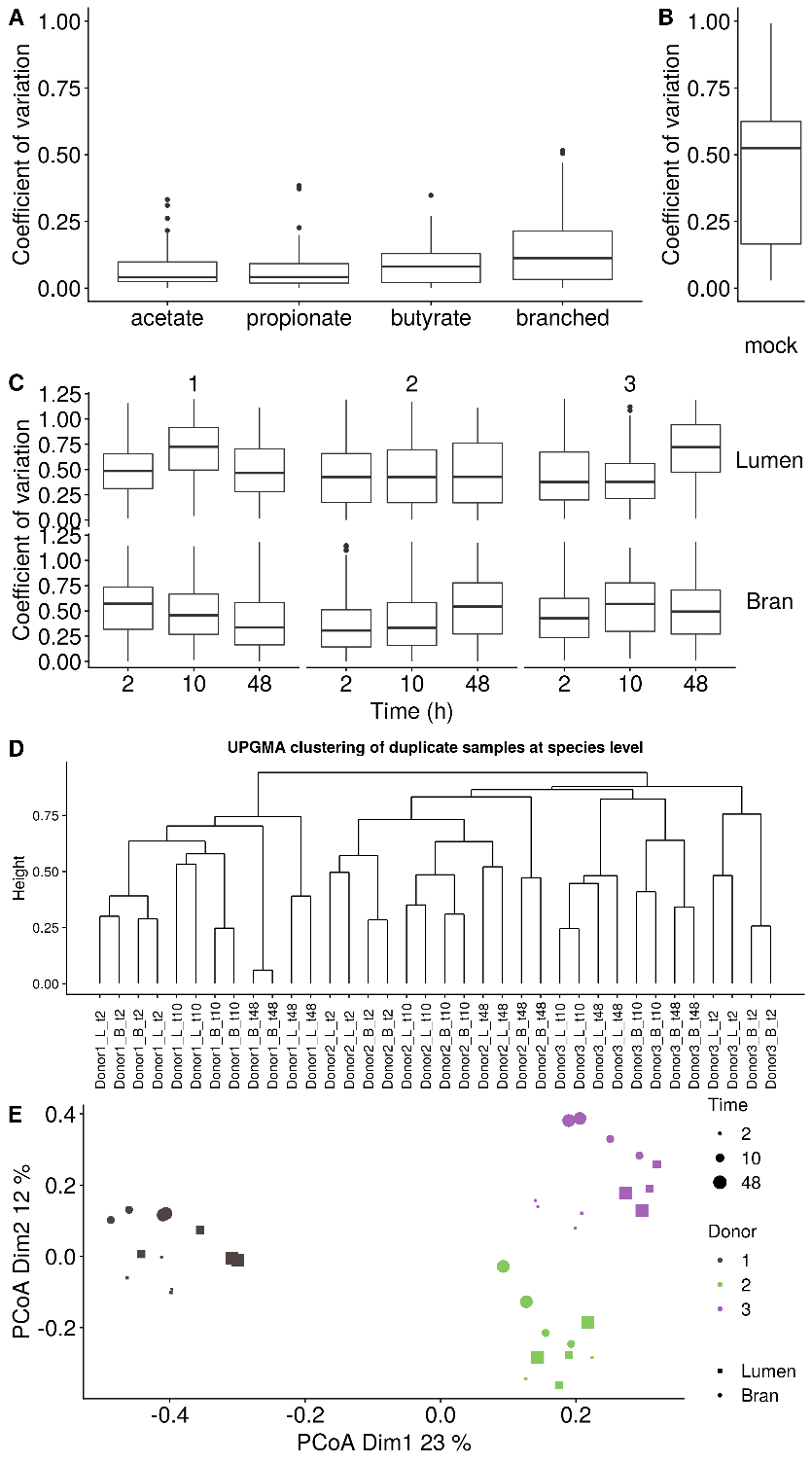


**Figure S17: Biological reproducibility of the SCFA concentrations (n=2 for all time points) (A) and 16S rRNA gene amplicon sequencing analysis (n = 2 for time points t2, 10 and 48) (C,D,E) of all donors. Technical reproducibility of the 16S rRNA gene amplicon sequencing analysis is assessed using a mock community (n=3) (B).** Box plots show the distribution of the coefficient of variation for all OTUs, calculated on biological replicates (n=2) (A,C) and technical replicates (n=3) of the mock community (B). Biological replicates cluster together, as assessed by UPGMA clustering of the abundance based jaccard dissimilarity matrix (D) and a Principle Coordinate Analysis (n=1) of the genus microbial community composition (E).


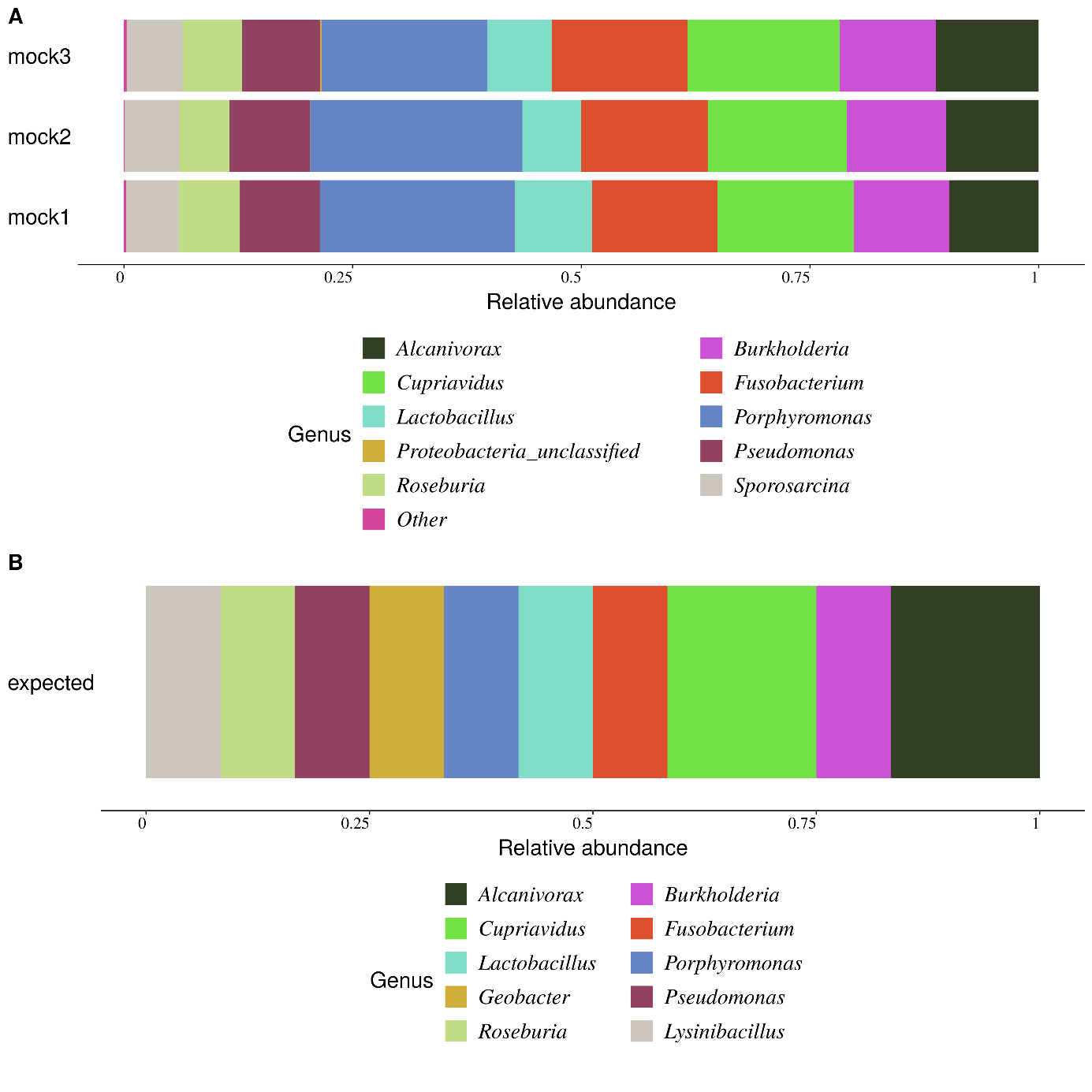


**Figure S18: Sequenced (A) and expected (B) composition of the mock community (n=3 replicates included in the same sequencing run).** The mock community was composed of mainly non-gut related taxa and was intended to verify the sequencing reproducibility (A) and number of spurious OTUs (comparing A and B). We found 16-10-16 spurious OTUs and a Coefficient of variation of around 50%.

**Supplementary tables in discussion:**

We have explored the possible underlying mechanisms of the observed microbial succession during wheat bran fermentation, by reviewing the available literature on the metabolic potency and growth conditions of the most important taxa observed in this study (Supplementary Information; Table S3-S7). If available, data concerning minimum generation time, pH range and optimum and oxygen sensitivity is reported (Table S3). Although incubations were performed in an anaerobic atmosphere, the latter might be important, as the damage induced by the short-term exposure to oxygen during the processing of the faecal sample, requires cell repair mechanisms to be activated prior to growth, thereby extending the lag phase (Rolfe et al 2012). In that sense, oxygen tolerance could result in a rapid onset of growth.

The metabolic potency to degrade wheat bran carbohydrates was evaluated combining a literature and a database search of the Carbohydrate-Active enzyme (CAZy) database (Table S5,6) (Bateman et al 2017, Cantarel et al 2009, Caspi et al 2016, Lombard et al 2014). Wheat bran consists of easily fermentable components (starch, proteins and part of the arabinoxylan fraction) entangled with more recalcitrant fibres (lignin, cellulose) (Flint et al 2008, Mirande et al 2010). Little is known regarding the first step of wheat bran degradation, involving the release and solubilisation of fibres from the complex insoluble wheat bran substrate. The subsequent enzymatic decomposition of the constituent wheat bran fibres is extensively studied. The degradation of cellulose requires the concerted action of cellulases (= endo-β-1,4-glucanase, EC 3.2.1.4) for the internal cleavage of cellulose microfibrils in amorphous regions and exo-β-1,4-glucanase, releasing cellobiose from the non-reducing (EC 3.2.1.91) and reducing end (EC 3.2.1.176) (Flint et al 2012, Quiroz-Castañeda and Folch-Mallol 2013). The final hydrolysis of cellobiose and cello-oligosaccharides to glucose is carried out by β-glucosidases (EC 3.2.1.21) (Adesogan et al 2014, Flint et al 2012, Quiroz-Castañeda and Folch-Mallol 2013). The cellulolytic activity releases the arabinoxylans from the cell wall (solubilisation), which can be further extracellularly degraded by endo-1,4-β-xylanase (EC 3.2.1.8) (Butt et al 2008, Dodd and Cann 2009). The generated unsubstituted or branched fragments are hydrolysed by i) β-xylosidase (EC 3.2.1.37), removing single xylose residues from the non-reducing end of xylo-oligosaccharides, in combination with ii) reducing end xylose-releasing exo-oligoxylanases (EC [3.2.1.156](http://enzyme.expasy.org/EC/3.2.1.156)) and iii) α -L-arabinofuranosidases (EC 3.2.1.55), removing the arabinose substituents (Dodd and Cann 2009, Hong et al 2014, Lagaert et al 2011). The feruloyl esterases (EC 3.1.1.73 ) attack ester bonds between the arabinose residues and phenolic acids (mainly monomeric or dimeric ferulic acid and p-coumaric acid) (Anson et al 2012). Analogously, the acetylxylan esterase (EC 3.1.1.72) removes acetyl residues from acetylated xylose (Dodd and Cann 2009). Finally, α-glucuronidase (EC 3.2.1.139) removes 4-O-methyl-D-glucuronic acid residues linked to xylose (Biely, 1985). Wheat bran contains residual starch as a results of the crude milling process (Hemdane et al 2016, United States Department of Agriculture (USDA) 2016). Although starch is usually efficiently digested during upper gastrointestinal tract passage by α-amylase activity (EC.3.2.1.1), some remaining starch can be present in the wheat bran matrix (Walter and Ley 2011). Next to endo- and exoamylases (β-amylase EC 3.2.1.2, glucan-1,4-α-glucosidase EC 3.2.1.3 and alpha-glucosidase EC 3.2.1.20), debranching enzymes (pullulanase EC 3.2.1.41 and isoamylase EC 3.2.1.68) and transferases (cyclomaltodextrin D-glucotransferase EC 2.4.1.19, 4-α-glucanotransferase EC 2.4.1.25 and 1,4-α-glucan branching enzyme EC 2.4.1.18) aid in starch degradation (Guimarães 2012, Kennedy et al 1988, van der Maarel et al 2002).

The enzymatic activity is not experimentally characterised for all the taxa of interest in this study. Therefore, to obtain an indication of the wheat bran degrading enzymatic capacity, the CAZy database was used to identify putative carbohydrate-active enzymes (CAZymes) and carbohydrate-binding modules (CBM), responsible for the binding of insoluble substrates (Table S5,S6). The CAZy database provides a GH family classification because the tremendous diversity in enzyme activity and substrate specificity, and underlying amino acid and genomic coding sequences impedes the accurate automatic assignment of enzyme activity based on genomic information (Henrissat 1991, Henrissat and Davies 1997). A glycosyl hydrolase (GH) family is characterised by a conserved enzyme structure, which is determined by and as a consequence can be predicted from the amino acid sequence (Henrissat 1991). GH families can contain enzymes representing a broad range of activities and substrates (Aspeborg et al 2012, El Kaoutari et al 2013). GH targeting insoluble substrates are appended with CBM, which are non-catalytic domains that recognise plant cell wall (including cellulose, xylan) and storage (including starch) polysaccharides and derived oligosaccharides (Boraston et al 2004). As outlined above the insoluble nature of wheat bran, reduces the accessibility of the substrate to enzymes and the presence of CBM concentrates the enzyme in close proximity of the substrate, thereby enhancing the enzymatic degradation of the substrate. The CBM families containing any of the above described enzyme activities, occurring in any of the taxa of interest, were explored in CAZy.

We also took into account the possibility that certain bacterial populations efficiently grew on the carbohydrate-low medium, which consisted of 3 g L^-1^ yeast-extract, 1 g L^-1^ peptone and 1 g L^-1^ mucin. In order to elucidate the negative or positive co-occurrence interactions between the predominant taxa, antimicrobial activity and cross-feeding were examined (Table S4,S7). Finally, to account for the attachment of bacteria to the insoluble wheat bran substrate, adhesive properties were reviewed (Table S4).

**Table S3: Growth conditions of the most important species level taxa observed in the microbial succession pattern in this study.** RDP SeqMatch and NCBI BLAST scores for the database entries showing the highest sequence similarity to the OTUs retrieved in this study are displayed. In case of tying scores, i.e. for OTU 1 and 20, all entries with the same score are incorporated. *F. nucleatum* characteristics are included as an addition to the poorly characterised species *F. mortiferum* (OTU5). If no experimentally determined values were available in literature, the DSMZ database was consulted for the optimum growth pH and the minimum generation time was deduced from the AGORA based predictions according to Magnúsdóttir et al (2017).

|  | **pH range** | **pH optimum** | **Minimum generation time** | **Oxygen sensitivity** |
| --- | --- | --- | --- | --- |
| ***Fusobacterium*** |  |  |  |  |
| *F. mortiferum*  (OTU5. Sab=0.995. Identity=100%) |  | 7 | 2.6 h (Magnúsdóttir et al 2017) |  |
| *F. nucleatum* | 5.5-7.8 (Zilm et al 2007) | 7.4 (Zilm et al 2007) | 40 min  (Mangels et al 1978) | moderately tolerant (Diaz et al 2000, Diaz et al 2002, Farias et al 1999) |
| ***Enterobacteriaceae*** |  | | |  |
| *Escherichia fergusonii* (OTU1.Sab=1.Identity=100%) |  | 7 | 32 min (Magnúsdóttir et al 2017) | facultative anaerobic (Gaastra et al 2014) |
| *Shigella sonnei* (OTU1.Sab=1.Identity=100%) | 4.5 (Bagamboula et al 2002) | 7 |  | facultative anaerobic (Hale and Keusch 1996) |
| *Shigella flexneri*  (OTU1.Sab=1.Identity=100%) | 4.75-9 (Bagamboula et al 2002, Zaika et al 1989) | 7 (Zaika et al 1989) | 18 min (Zaika et al 1989) | facultative anaerobic (Hale and Keusch 1996) |
| *Escherichia coli* (OTU1.Sab=0.983.Identity=100%) | 4.0-8.0 (Presser et al 1997) | 7 (Presser et al 1997) | 20 min (Rubin 1986) | facultative anaerobic (Iuchi and Weiner 1996, Morris and Schmidt 2013) |
| *Pantoea vagans* (OTU15.Sab=1.Identity=100%) |  | 7 |  | facultative anaerobic (Brady et al 2009) |
| ***Bacteroidetes*** |  |  |  |  |
| *Bacteroides uniformis* (OTU3.Sab=1.Identity=100%) | impaired growth <6 (Duncan et al 2009, Lawley and Walker 2013) | 7.2 | 2 h (Sonnenburg et al 2010) | strictly anaerobic (Wexler 2007) |
| *Bacteroides stercoris* (OTU4.Sab=0.981.Identity=100%) | impaired growth <6 (Duncan et al 2009, Lawley and Walker 2013) | 7 | 1.5 h(Magnúsdóttir et al 2017) | strictly anaerobic (Johnson et al 1986, Wexler 2007) |
| *Bacteroides ovatus* (OTU6.Sab=0.978.Identity=99%) | impaired growth <6 (Duncan et al 2009, Lawley and Walker 2013) | 7 | 1.4 h (Sonnenburg et al 2010) | strictly anaerobic (Wexler 2007) |
| *Prevotella copri* (OTU8/9.Sab=0.93/0.969.Identity=99/99%) | impaired growth <6 (Duncan et al 2009, Lawley and Walker 2013) | 7.3 | 3.8 h (Magnúsdóttir et al 2017) | strictly anaerobic (Hayashi et al 2007) |
| ***Firmicutes*** | **More acid tolerant (Chung et al 2016, Duncan et al 2009)** |  |  |  |
| *Clostridium xylanolyticum* (OTU7.Sab=0.857.Identity=98%) |  | 7.2 |  | strictly anaerobic (Chamkha et al 2001, Rogers and Baecker 1991) |
| *Coprococcus eutactus* (OTU17.Sab=0.982.Identity=99%) |  |  | 3.9 h (Magnúsdóttir et al 2017) | strictly anaerobic (Holdeman and Moore 1974) |
| *Roseburia faecis* (OTU18.Sab=1.Identity=100%) |  | 6.8 |  | strictly anaerobic (Duncan et al 2006) |
| *Clostridium bolteae* (OTU26.Sab=0.814.Identity=96%) | 6.8-7 (Song et al 2003) |  | 2 h (Magnúsdóttir et al 2017) | strictly anaerobic (Song et al 2003) |
| *Oscillibacter ruminantium* (OTU31.Sab=0.798.Identity=96%) | 5.5-6.5 (Lee et al 2013) | 6 (Lee et al 2013) |  | strictly anaerobic (Lee et al 2013) |
| *Eubacterium siraeum* (OTU53.Sab=0.954.Identity=99%) |  | 7 | 6 h (Magnúsdóttir et al 2017) | strictly anaerobic (Moore et al 1976) |
| *Clostridium asparigiforme* (OTU54.Sab=0.902.Identity=97%) |  |  | 1.9 h (Magnúsdóttir et al 2017) | strictly anaerobic (Mohan et al 2006) |
| *Ruminococcus champanellensis* (OTU153.Sab=0.966.Identity=99%) | 6.5-6.8 (Chassard et al 2012) | 6.8 (Chassard et al 2012) | 1.9 h (Chassard et al 2012) | strictly anaerobic (Chassard et al 2012) |
| ***Bifidobacterium*** | **Prefer lower pH (Chung et al 2016)** |  |  |  |
| *Bifidobacterium faecale* (OTU20.Identity=100%) | 5-7 (Choi et al 2014) | 7 |  | strictly anaerobic (Choi et al 2014) |
| *Bifidobacterium adolescentis* (OTU20.Sab=1.Identity=100%) | 5.5 (Duncan et al 2009) | 5.5 (Amaretti et al 2007) | 1.3 h (Rios-Covian et al 2015) | strictly anaerobic (Simpson et al 2005) |

**Table S4: Antimicrobial activity and adhesive properties of the most important species level taxa observed in the microbial succession pattern in this study.** RDP SeqMatch and NCBI BLAST scores for the database entries showing the highest sequence similarity to the OTUs retrieved in this study are displayed. In case of tying scores, i.e. for OTU1 and 20, all entries with the same score are incorporated. *F. nucleatum* characteristics are included as an addition to the poorly characterised species *F. mortiferum* (OTU5). Antimicrobial activity can be exerted by the production of bacteriocin like inhibitory substances (BLIS), targeting species within the same genus (iso-antagonistic) or unrelated species belonging to other genera (hetero-antagonism). If no experimental data was available in literature, we reported the inferred functionality based on genomic information, retrieved by querying the Uniprot gene ontology field for the terms ‘bacteriocin activity’, ‘antimicrobial peptide activity’, ‘cell adhesion’, ‘fimbria, ‘glycocalyx’ and ‘capsule polysaccharide anabolism’ (Bateman et al 2017, Zheng et al 2015).

|  | **Antimicrobial substances** | **Adhesive properties** |
| --- | --- | --- |
| ***Fusobacterium*** |  |  |
| *F. mortiferum* (OTU5, Sab=0,995, Identity=100%) | Hetero-antagonistic BLIS (Portrait et al 2000) | Adhesins (Han 2015, McGuire et al 2014) |
| *F. nucleatum* |  | Expresses numerous adhesins involved in host-cells adhesion (associated with potential to cause disease) and inter-species co-aggregation in the context of oral biofilm formation (Bradshaw et al 1998, Han 2015, McGuire et al 2014, Park et al 2016) |
| ***Enterobacteriaceae*** |  |  |
| *Escherichia fergusonii* (OTU1,Sab=1,Identity=100%) | Iso-antagonistic BLIS: colicin (Gaastra et al 2014) | Fimbrial adhesins in some isolates (Gaastra et al 2014) |
|  |  | Putative genes for flagellar biosynthesis (Gaastra et al 2014) |
| *Shigella sonnei* (OTU1,Sab=1,Identity=100%) | Iso-antagonistic BLIS: colicin (Cascales et al 2007, Sousa et al 2010) | Adhesins (The et al 2016) |
| *Shigella flexneri* (OTU1,Sab=1,Identity=100%) | Iso-antagonistic BLIS: colicin (Cascales et al 2007, Sousa et al 2010) | Adhesins (The et al 2016) |
| *Escherichia coli* (OTU1,Sab=0,983,Identity=100%) | Iso-antagonistic BLIS: colicin (Cascales et al 2007, Sousa et al 2010, van Heel et al 2013) | Adhesins (Le Bouguenec 2005) |
| *Pantoea vagans* (OTU15,Sab=1,Identity=100%) | Lysozyme inferred from homology (Bateman et al 2017) | Flagellar protein inferred from homology and predicted fimbrial proteins (Bateman et al 2017) |
| ***Bacteroidetes*** |  |  |
| *Bacteroides uniformis* (OTU3,Sab=1,Identity=100%) | Iso-antagonistic BLIS (Nakano et al 2013) | Putative fimbrium subunit (experimental evidence at protein level) (Bateman et al 2017, Madej et al 2014) |
|  | Predicted BLIS (Zheng et al 2015) | Capsule identified (Nakano et al 2008) |
| *Bacteroides stercoris* (OTU4,Sab=0,981,Identity=100%) | Predicted BLIS (Zheng et al 2015) |  |
| *Bacteroides ovatus* (OTU6,Sab=0,978,Identity=99%) | Hetero-antagonistic BLIS (Nakano et al 2013) | Putative fimbrium tip subunit Fim1C (experimental evidence at protein level) (Bateman et al 2017) |
| *Prevotella copri* (OTU8/9,Sab=0,93/0,969,Identity=99/99%) | Predicted BLIS (Zheng et al 2015) | Capsule identified (Babb and Cummins 1978, Coyne and Comstock 2008) |
|  |  |  |
| ***Firmicutes*** |  |  |
| *Clostridium xylanolyticum* (OTU7,Sab=0,857,Identity=98%) |  | Peritrichous flagella (Rogers and Baecker 1991) |
| *Coprococcus eutactus* (OTU17,Sab=0,982,Identity=99%) |  | Flagellar proteins inferred from homology (Bateman et al 2017) |
| *Roseburia faecis* (OTU18,Sab=1,Identity=100%) | BLIS active against B. subtilis (Hatziioanou et al 2013) | Flagella (Louis and Flint 2009) |
| *Clostridium bolteae* (OTU26,Sab=0,814,Identity=96%) | Lysozyme inferred from homology (Bateman et al 2017) | Flagellar protein inferred from homology (Bateman et al 2017, Dehoux et al 2016) |
| *Oscillibacter ruminantium* (OTU31,Sab=0,798,Identity=96%) |  | Peritrichous flagella (Lee et al 2013) |
| *Eubacterium siraeum* (OTU53,Sab=0,954,Identity=99%) |  | Flagellar proteins inferred from homology (Bateman et al 2017) |
| *Clostridium asparigiforme* (OTU54,Sab=0,902,Identity=97%) |  |  |
| *Ruminococcus champanellensis* (OTU153,Sab=0,966,Identity=99%) |  | Cellulosome (Morais et al 2016) |
| ***Bifidobacterium*** |  |  |
| *Bifidobacterium faecale* (OTU20,Identity=100%) |  |  |
| *Bifidobacterium adolescentis* (OTU20,Sab=1,Identity=100%) |  | Predicted collagen adhesins (Bateman et al 2017) |

**Table S5: Distribution of glycosyl hydrolase (GH) families involved in wheat bran degradation among the species level taxa observed in the microbial succession pattern in this study.** Within each GH family, the experimentally validated enzyme activities and the corresponding taxa are displayed in bold face. In all other cases results should be interpreted with caution (Cantarel et al 2009), since the heterogeneity of enzyme activities and ligand specificity in GH and CBM families limits functional prediction (Aspeborg et al 2012, Boraston et al 2004).

|  | Aarabinoxylan degradation | | | | | | | Cellulose degradation | | | | Starch degradation | | | | | Note: experimentally validated monospecific enzyme activities within each GH family are inserted in a separate row below the GH and the taxa are displayed in bold Note: * experimentally determined PUL Note: *Prevotella copri*, *Clostridium xylanolyticum*, *Roseburia faecis*, *Clostridium asparagiform*e, *Oscillibacter ruminantium*, *Bifidobacterium faecale* are not present in the CAZy database |
| --- | --- | --- | --- | --- | --- | --- | --- | --- | --- | --- | --- | --- | --- | --- | --- | --- | --- |
|  | endo-1,4-β-xylanase | acetylxylanesterase | α-glucuronidase | feruloyl esterase | α-L-arabinofuranosidase | reducing end xylose-releasing exo-oligoxylanases | β-xylosidase | endo-β-1,4-glucanase | exo-glucanase | | β-glucosidase | α-amylase | β-amylase | glucan-1,4-α-glucosidase | alpha-glucosidase | pullulanase |  |
|  | 3.2.1.8 | 3.1.1.72 | 3.2.1.139 | 3.1.1.73 | 3.2.1.55 | 3.2.1.156 | 3.2.1.37 | 3.2.1.4 | 3.2.1.91 | 3.2.1.176 | 3.2.1.21 | 3.2.1.1 | 3.2.1.2 | 3.2.1.3 | 3.2.1.20 | 3.2.1.41 | Species |
| GH1 |  |  |  |  |  |  |  |  |  |  |  |  |  |  |  |  | *F.mortiferum;E.fergusonii;.sonnei;E.coli;P.vagans;B.adolescentis; C.eutactus;R.champanellensis* |
| GH2 |  |  |  |  |  |  |  |  |  |  |  |  |  |  |  |  | *E.fergusonii;S.sonnei;E.coli;P.vagans;Buniformis;B.ovatus;B.adolescentis;C.eutactus;*  *C.siraeum;R.champanellensis* |
| GH3 |  |  |  |  |  |  |  |  |  |  |  |  |  |  |  |  | *E.fergusonii;S.sonnei;E.coli;P.vagans;B.uniformis;B.ovatus;B.adolescentis;C.eutactus;*  *E.siraeum;R.champanellensis* |
|  |  |  |  |  |  |  |  |  |  |  |  |  |  |  |  |  | ***E.coli;B.uniformis;B.ovatus*** |
| GH4 |  |  |  |  |  |  |  |  |  |  |  |  |  |  |  |  | *F.mortiferum;E.fergusonii;E.coli* |
| GH5 |  |  |  |  |  |  |  |  |  |  |  |  |  |  |  |  | *E.coli;B.ovatus;B.adolescentis;C.eutactus;C.siraeum;R.champanellensis* |
|  |  |  |  |  |  |  |  |  |  |  |  |  |  |  |  |  | ***R.champanellensis*** |
| GH8 |  |  |  |  |  |  |  |  |  |  |  |  |  |  |  |  | *E.fergusonii;S.sonnei;E.coli;P.vagans;B.adolescentis;R.champanellensis* |
|  |  |  |  |  |  |  |  |  |  |  |  |  |  |  |  |  | ***B.adolescentis*** |
|  |  |  |  |  |  |  |  |  |  |  |  |  |  |  |  |  | ***R.champanellensis*** |
| GH9 |  |  |  |  |  |  |  |  |  |  |  |  |  |  |  |  | *F.mortiferum;E.coli;B.ovatus;C.eutactus;R.champanellensis* |
|  |  |  |  |  |  |  |  |  |  |  |  |  |  |  |  |  | ***R.champenellensis*** |
| GH10 |  |  |  |  |  |  |  |  |  |  |  |  |  |  |  |  | *B.ovatus;R.champanellensis* |
|  |  |  |  |  |  |  |  |  |  |  |  |  |  |  |  |  | ***B.ovatus*** |
| GH11 |  |  |  |  |  |  |  |  |  |  |  |  |  |  |  |  | *R.champanellensis* |
|  |  |  |  |  |  |  |  |  |  |  |  |  |  |  |  |  | ***R.champenellensis*** |
| GH13 |  |  |  |  |  |  |  |  |  |  |  |  |  |  |  |  | *F.nucleatum;E.fergusonii;S.sonnei;E.coli;P.vagans;B.ovatus;B.adolescentis;C.eutactus;E.siraeum;R.champanellensis* |
|  |  |  |  |  |  |  |  |  |  |  |  |  |  |  |  |  | ***E.coli*** |
|  |  |  |  |  |  |  |  |  |  |  |  |  |  |  |  |  | ***E.coli;B.adolescentis*** |
| GH15 |  |  |  |  |  |  |  |  |  |  |  |  |  |  |  |  | *P.vagans;B.ovatus* |
| GH30 |  |  |  |  |  |  |  |  |  |  |  |  |  |  |  |  | *B.ovatus;B.adolescentis;R.champanellensis* |
|  |  |  |  |  |  |  |  |  |  |  |  |  |  |  |  |  | ***B.adolescentis*** |
|  |  |  |  |  |  |  |  |  |  |  |  |  |  |  |  |  | ***B.ovatus, R.champanellensis*** |
| GH39 |  |  |  |  |  |  |  |  |  |  |  |  |  |  |  |  | *E.coli;B.adolescentis* |
| GH43 |  |  |  |  |  |  |  |  |  |  |  |  |  |  |  |  | *E.coli;P.vagans;B.ovatus;B.adolescentis,C.siraeum;R.champanellensis* |
|  |  |  |  |  |  |  |  |  |  |  |  |  |  |  |  |  | ***B.ovatus, B.adolescentis, R.champanellensis*** |
|  |  |  |  |  |  |  |  |  |  |  |  |  |  |  |  |  | ***B.ovatus,B.adolescentis*** |
| GH44 |  |  |  |  |  |  |  |  |  |  |  |  |  |  |  |  | *R.champanellensis* |
| GH51 |  |  |  |  |  |  |  |  |  |  |  |  |  |  |  |  | *B.ovatus;B.adolescentis;C.eutactus* |
|  |  |  |  |  |  |  |  |  |  |  |  |  |  |  |  |  | ***B.ovatus;B.adolescentis*** |
| GH57 |  |  |  |  |  |  |  |  |  |  |  |  |  |  |  |  | *B.ovatus* |
| GH67 |  |  |  |  |  |  |  |  |  |  |  |  |  |  |  |  | *B.ovatus* |
|  |  |  |  |  |  |  |  |  |  |  |  |  |  |  |  |  | ***B.ovatus*** |
| GH74 |  |  |  |  |  |  |  |  |  |  |  |  |  |  |  |  | *R.champanellensis* |
| GH98 |  |  |  |  |  |  |  |  |  |  |  |  |  |  |  |  | *B.ovatus;R.champanellensis* |
|  |  |  |  |  |  |  |  |  |  |  |  |  |  |  |  |  | ***B.ovatus*** |
| GH116 |  |  |  |  |  |  |  |  |  |  |  |  |  |  |  |  | *B.uniformis* |
| GH120 |  |  |  |  |  |  |  |  |  |  |  |  |  |  |  |  | *B.adolescentis* |
|  |  |  |  |  |  |  |  |  |  |  |  |  |  |  |  |  | ***B.adolescentis*** |
| CE1 |  |  |  |  |  |  |  |  |  |  |  |  |  |  |  |  | *E.fergusoni;S.flexneri;E.coli;B.ovatus;R.champanellensis* |
| CE2 |  |  |  |  |  |  |  |  |  |  |  |  |  |  |  |  | *C.eutactus;C.siraeum;R.champanellensis* |
| CE3 |  |  |  |  |  |  |  |  |  |  |  |  |  |  |  |  | *R.champanellensis* |
| CE4 |  |  |  |  |  |  |  |  |  |  |  |  |  |  |  |  | *F.nucleatum;E.coli;P.vagans;B.ovatus;R.champanellensis* |
| CE5 |  |  |  |  |  |  |  |  |  |  |  |  |  |  |  |  | *E.siraeum* |
| CE6 |  |  |  |  |  |  |  |  |  |  |  |  |  |  |  |  | *B.ovatus* |
| CE7 |  |  |  |  |  |  |  |  |  |  |  |  |  |  |  |  | *B.ovatus* |
| CE12 |  |  |  |  |  |  |  |  |  |  |  |  |  |  |  |  | *B.ovatus;R.champanellensis* |
| PUL |  |  |  |  |  |  |  |  |  |  |  |  |  |  |  |  | *B.ovatus*,Prevotella copri* |
|  |  |  |  |  |  |  |  |  |  |  |  |  |  |  |  |  | *B.uniformis,B.ovatus*,Prevotella copri* |

**Table S6: Distribution of Carbohydrate-Binding Modules (CBM) involved in the binding of insoluble wheat bran constituents among the species level taxa observed in the microbial succession pattern in this study.** Within each CBM family, the experimentally validated CMBs are shown and the corresponding taxa are displayed in bold face. The enzyme activity of the GH, equipped with the CBM is shown between brackets. In all other cases results should be interpreted with caution (Boraston et al 2004).

|  | Xylan | Cellulose | | Starch | | | | | Note: *Prevotella copri*, *Clostridium xylanolyticum*, *Roseburia faecis*, *Clostridium asparagiform*e, *Oscillibacter ruminantium*, *Bifidobacterium faecale* are not present in the CAZy database |
| --- | --- | --- | --- | --- | --- | --- | --- | --- | --- |
|  |  | not-specified | amporhous | granular starch | starch | amylose | amylopectin | pullulan | Species |
| CBM2 |  |  |  |  |  |  |  |  | *C.eutactus* |
| CBM3 |  |  |  |  |  |  |  |  | *R.champanellensis* |
|  |  |  |  |  |  |  |  |  | ***R.champanellensis* (xylanase)** |
| CBM4 |  |  |  |  |  |  |  |  | *E.coli,B.ovatus,C.eutactus,E.siraeum,R.champanellensis* |
| CBM6 |  |  |  |  |  |  |  |  | *B.ovatus,E.siraeum,R.champanellensis* |
|  |  |  |  |  |  |  |  |  | ***R.champanellensis* (bifunctional arabinofuranosidase, xylanase)** |
| CBM13 |  |  |  |  |  |  |  |  | *C.eutactus,R.champanellensis,B.adolescentis* |
|  |  |  |  |  |  |  |  |  | ***R.champanellensis* (arabinase)** |
| CBM20 |  |  |  |  |  |  |  |  | *B.ovatus* |
| CBM22 |  |  |  |  |  |  |  |  | *R.champanellensis* |
|  |  |  |  |  |  |  |  |  | ***R.champanellensis* (xylanase + bifunctional xylanase, arabinofuranosidase)** |
| CBM25 |  |  |  |  |  |  |  |  | *B.adolescentis* |
| CBM26 |  |  |  |  |  |  |  |  | *B.adolescentis* |
| CBM30 |  |  |  |  |  |  |  |  | *F.mortiferum* |
| CBM34 |  |  |  |  |  |  |  |  | *E.fergusonii,S.flexneri,E.coli,C.eutactus,C.bolteae* |
|  |  |  |  |  |  |  |  |  | *E.coli* (α-glucosidase and maltodextrin glucosidase) |
| CBM35 |  |  |  |  |  |  |  |  | *B.ovatus,E.siraeum* |
| CBM41 |  |  |  |  |  |  |  |  | *B.adolescentis* |
| CBM60 |  |  |  |  |  |  |  |  | *S.sonnei* |
| CBM69 |  |  |  |  |  |  |  |  | *E.coli* |
| CBM74 |  |  |  |  |  |  |  |  | *B.adolescentis* |

**Table S7: Metabolic capacity of the most important species level taxa observed in the microbial succession pattern in this study.** RDP SeqMatch and NCBI BLAST scores for the database entries showing the highest sequence similarity to the OTUs retrieved in this study are displayed. In case of tying scores, i.e. for OTU 1 and 20, all entries with the same score are incorporated. *F. nucleatum* characteristics are included as an addition to the poorly characterised species *F. mortiferum* (OTU5).

|  | **Fermentation substrates** | **SCFA production** |
| --- | --- | --- |
| ***Fusobacterium*** |  |  |
| *F. mortiferum* (OTU5, Sab=0,995, Identity=100%) | Proteolytic fermentation: grows well on medium containing yeast-extract, peptone (Robrish et al 1991) |  |
|  | Saccharolytic fermentation: ferments cellobiose, glucose (Robrish et al 1991, Thompson et al 1997) |  |
|  | P-β-glucosidase (EC 3.2.1.86) activity shown (Thompson et al 1997) |  |
| *F. nucleatum* | Proteolytic fermentation: grows well on medium containing yeast-extract, peptone (Robrish et al 1991) | Ethanol, Formate, Lactate, Acetate, Butyrate (Diaz et al 2000) |
| ***Enterobacteriaceae*** |  |  |
| *Escherichia fergusonii* (OTU1,Sab=1,Identity=100%) | Proteolytic fermentation (Marzorati et al 2017, Richardson et al 2013) |  |
|  | Saccharolytic fermentation: ferments D-xylose, L-arabinose, glucose (Farmer et al 1985, Gaastra et al 2014) |  |
| *Shigella sonnei* (OTU1,Sab=1,Identity=100%) |  |  |
| *Shigella flexneri* (OTU1,Sab=1,Identity=100%) |  |  |
| *Escherichia coli* (OTU1,Sab=0,983,Identity=100%) | Saccharolytic fermentation: ferments xylose, L-arabinose, glucose (Brady et al 2009, Salamanca-Cardona et al 2014) | Ethanol, Formate, Lactate, Succinate, Acetate, H_2_, CO_2_ (Clark 1989) |
|  | Unable to degrade cellulose (Gao et al 2015) |  |
|  | Unable to utilise xylan (Salamanca-Cardona et al 2014) |  |
|  | Periplasmic endo-glucanase activity experimentally confirmed (synthesis and membrane translocation of cellulose) (Mazur and Zimmer 2011) |  |
| *Pantoea vagans* (OTU15,Sab=1,Identity=100%) | Saccharolytic fermentation: ferments xylose, L-arabinose, cellobiose, glucose (Brady et al 2009) |  |
| ***Bacteroidetes*** |  | **Acetate, Propionate, Succinate, H_2_, Lactate (Chassard et al 2007)** |
| *Bacteroides uniformis* (OTU3,Sab=1,Identity=100%) | Saccharolytic fermentation: ferments xylose, L-arabinose, arabinoxylan oligosaccharides (AXOS), cellobiose, starch, glucose (Benítez-Páez et al 2017, Johnson and Ault 1978) | Propionate, Succinate |
| *Bacteroides stercoris* (OTU4,Sab=0,981,Identity=100%) | Saccharolytic fermentation: ferments xylose, L-arabinose, xylan, cellobiose, starch, glucose (Johnson et al 1986) | Acetate, Propionate, Succinate, Formate, isobutyrate, Isovalerate (Johnson et al 1986) |
|  | β-gucosidase (EC 3.2.1.21) activity experimentally confirmed (Johnson et al 1986) |  |
|  | Equipped with an array of enzymes (PL8,PL12,PL13,PL21) dedicated to the use of glycosaminoglycans  (Ahn et al 1998, Cantarel et al 2009, Lombard et al 2014, Tripathi et al 2012) |  |
| *Bacteroides ovatus* (OTU6,Sab=0,978,Identity=99%) | Proteolytic fermentation: Able to use peptides from a yeast-extract containing YCFA medium (Scott et al 2013) | Acetate, Propionate, Succinate, Lactate (Martin et al 1998) |
|  | Saccharolytic fermentation: ferments wheat bran, xylose, L-arabinose, glucuronic acid, arabinoxylan, xylan, cellobiose, starch, amylopectin, pullulan, dextran, glucose (Duncan et al 2016, Johnson and Ault 1978, Martens et al 2011, Martin et al 1998, Rogowski et al 2016, Salyers et al 1981, Zhang et al 2014) |  |
|  | β-glucosidase activity (EC 3.2.1.21) experimentally confirmed (McBain and Macfarlane 1998) |  |
|  | Xylanase (EC 3.2.1.8) activity experimentally confirmed (Chassard et al 2007)  Xylanase and xylosidase, arabinofuranosidase encoding genes identified (Weaver et al 1992) |  |
|  | Possesses membrane associated Xylan Utilization Systems (Xus) directed to the efficient utilisation of soluble xylan through the coordinated cellular attachment, depolymerization, transport and further intracellular degradation of oligosaccharides (Dodd et al 2011, McNulty et al 2013, Rogowski et al 2016, White et al 2014) |  |
|  | Unable to utilise cellulose (Martens et al 2011) |  |
|  |  |  |
| *Prevotella copri* (OTU8/9,Sab=0,93/0,969,Identity=99/99%) | Saccharolytic fermentation: ferments xylose, L-arabinose, glucose (Hayashi et al 2007) | Succinate, Acetate (Hayashi et al 2007) |
| ***Firmicutes*** |  |  |
| *Clostridium xylanolyticum* (OTU7,Sab=0,857,Identity=98%) | Saccharolytic fermentation: ferments xylan, xylose, glucose (Rogers and Baecker 1991) | Formate, Lactate, Acetate, Ethanol (Broda et al 2000, Chamkha et al 2001) |
|  | Metabolises ferulic acid (Chamkha et al 2001) |  |
|  | Unable to ferment L-arabinose (Chamkha et al 2001) |  |
| *Coprococcus eutactus* (OTU17,Sab=0,982,Identity=99%) | Saccharolytic fermentation: ferments cellobiose and glucose (Holdeman and Moore 1974) | Formate, Butyrate, Lactate, Acetate, Ethanol, Pyruvate, Succinate (Holdeman and Moore 1974) |
|  | Unable to ferment arabinose or xylose (Holdeman and Moore 1974) |  |
|  | β-glucosidase activity (EC 3.2.1.21) experimentally confirmed (Dabek et al 2008) |  |
|  | Cellulase activity on CMC of cloned GH9 domain from *C. eutactus* demonstrated (Szczepańska 2011) |  |
|  | No cross-feeding on acetate or lactate (Louis and Flint 2009, Reichardt et al 2014) |  |
| *Roseburia faecis* (OTU18,Sab=1,Identity=100%) | Saccharolytic fermentation: ferments wheat bran, oat spelt xylan, xylose, cellobiose, xylo-oligosaccharides, arabinoxylans, starch, glucose (Duncan et al 2006, Sheridan et al 2016) | Butyrate, Formate, Lactate (Duncan et al 2006) |
|  | Unable to ferment arabinose (Duncan et al 2006) |  |
|  | β-glucosidase activity (EC 3.2.1.21) experimentally confirmed (Dabek et al 2008) |  |
|  | GH10 detected (Sheridan et al 2016) |  |
|  | Cross-feeding on acetate (Duncan et al 2006, Louis and Flint 2009) |  |
| *Clostridium bolteae* (OTU26,Sab=0,814,Identity=96%) | Saccharolytic fermentation: ferments xylose, L-arabinose, glucose (Song et al 2003) | Acetate, Lactate (Song et al 2003) |
| *Oscillibacter ruminantium* (OTU31,Sab=0,798,Identity=96%) | Saccharolytic fermentation: ferments D-xylose, glucose (Lee et al 2013) | Butyrate, Acetate, Ethanol, Butanol (Lee et al 2013) |
|  | Unable to ferment L-arabinose, cellobiose and starch (Lee et al 2013) |  |
| *Eubacterium siraeum* (OTU53,Sab=0,954,Identity=99%) | Saccharolytic fermentation: ferments wheat bran, xylan, xylose, cellobiose, starch (Moore et al 1976) | Acetate (Moore et al 1976) |
|  | β-glucosidase activity (EC 3.2.1.21) experimentally confirmed (Dabek et al 2008, Duncan et al 2016) |  |
| *Clostridium asparigiforme* (OTU54,Sab=0,902,Identity=97%) | Proteolytic fermentation: L-tryptophan (Mohan et al 2006) | Acetate, Lactate, Ethanol, Formate,H2 (Mohan et al 2006) |
|  | Saccharolytic fermentation: ferments glucose (Mohan et al 2006) |  |
|  | α-glucuronidase activity (EC 3.2.1.139) experimentally confirmed (Mohan et al 2006) |  |
| *Ruminococcus champanellensis* (OTU153,Sab=0,966,Identity=99%) | Saccharolytic fermentation: ferments xylan, cellulose, cellobiose (Chassard et al 2012) | Acetate, Succinate, Ethanol, Formate, Lactate, H_2_ (Chassard et al 2012) |
|  | Unable to ferment xylose, arabinose, starch, glucose, pectin or other carbohydrates (Chassard et al 2012) |  |
|  | Only gut bacterium reported to degrade microcrystalline cellulose (Morais et al 2016) |  |
|  | Cellulase (EC 3.2.1.4) and xylanase (EC 3.2.1.8) activity detected experimentally (Chassard et al 2012) |  |
|  | Esterase and β-glucosidase activity (EC 3.2.1.21) experimentally confirmed (Chassard et al 2012) |  |
|  | Cellulosome experimentally characterised (Morais et al 2016) |  |
| ***Bifidobacterium*** |  |  |
| *Bifidobacterium faecale* (OTU20,Identity=100%) | Saccharolytic fermentation: ferments L-arabinose, cellobiose, starch, glucose (Choi et al 2014) | Acetate, Lactate (Choi et al 2014) |
|  | Unable to ferment D-xylose (Choi et al 2014) |  |
|  | Esterase activity experimentally confirmed (Choi et al 2014) |  |
|  | β-glucosidase activity (EC 3.2.1.21) experimentally confirmed (Choi et al 2014) |  |
| *Bifidobacterium adolescentis* (OTU20,Sab=1,Identity=100%) | Saccharolytic fermentation: ferments D-xylose, arabinose, arabinoxylans, arabinoxylan oligosaccharides (AXOS), cellobiose, starch, glucose (Killer et al 2013, Rios-Covian et al 2015, Savard and Roy 2009, van den Broek et al 2008) | Acetate, Lactate, Ethanol (Amaretti et al 2007, Rios-Covian et al 2015) |
|  | α-arabinofuranosidase (EC 3.2.1.55) and β-glucosidase activity (EC 3.2.1.21) experimentally confirmed (Killer et al 2013) |  |

**References**

Adesogan AT, Ma ZX, Romero JJ, Arriola KG (2014). Improving cell wall digestion and animal performance with fibrolytic enzymes. *J Anim Sci* **92:** 1317-1330.

Ahn MY, Shin KH, Kim DH, Jung EA, Toida T, Linhardt RJ *et al* (1998). Characterization of a Bacteroides species from human intestine that degrades glycosaminoglycans. *Can J Microbiol* **44:** 423-429.

Amaretti A, Bernardi T, Tamburini E, Zanoni S, Lomma M, Matteuzzi D *et al* (2007). Kinetics and metabolism of Bifidobacterium adolescentis MB 239 growing on glucose, galactose, lactose, and galactooligosaccharides. *Appl Environ Microb* **73:** 3637-3644.

Anderson MJ, Ellingsen KE, McArdle BH (2006). Multivariate dispersion as a measure of beta diversity. *Ecol Lett* **9:** 683-693.

Anson NM, Hemery YM, Bast A, Haenen GRMM (2012). Optimizing the bioactive potential of wheat bran by processing. *Food & function* **3:** 362-375.

Aspeborg H, Coutinho PM, Wang Y, Brumer H, Henrissat B (2012). Evolution, substrate specificity and subfamily classification of glycoside hydrolase family 5 (GH5). *Bmc Evol Biol* **12**.

Babb JL, Cummins CS (1978). Encapsulation of Bacteriodes Species. *Infect Immun* **19:** 1088-1091.

Bagamboula CF, Uyttendaele M, Debevere J (2002). Acid tolerance of Shigella sonnei and Shigella flexneri. *J Appl Microbiol* **93:** 479-486.

Banerjee A, Dolado J, Galbraith JW, Hendry D (1993). Co-integration, Error Correction, and the Econometric Analysis of Non-Stationary Data. *Advanced Texts in Econometrics***:** 342.

Bateman A, Martin MJ, O'Donovan C, Magrane M, Alpi E, Antunes R *et al* (2017). UniProt: the universal protein knowledgebase. *Nucleic Acids Res* **45:** D158-D169.

Becker RA, Chambers JM, Wilks AR (1988). *The new S language: a programming environment for data analysis and graphics*. Wadsworth and Brooks/Cole Advanced Books & Software.

Benítez-Páez A, Gómez del Pulgar EM, Sanz Y (2017). The Glycolytic Versatility of Bacteroides uniformis CECT 7771 and Its Genome Response to Oligo and Polysaccharides. *Frontiers in Cellular and Infection Microbiology* **7**.

Boraston AB, Bolam DN, Gilbert HJ, Davies GJ (2004). Carbohydrate-binding modules: fine-tuning polysaccharide recognition. *Biochem J* **382:** 769-781.

Borcard D, Gillet F, Legendre P (2011). *Numerical Ecology with R*. Springer Sciece.

Bradshaw DJ, Marsh PD, Watson GK, Allison C (1998). Role of Fusobacterium nucleatum and coaggregation in anaerobe survival in planktonic and biofilm oral microbial communities during aeration. *Infect Immun* **66:** 4729-4732.

Brady CL, Venter SN, Cleenwerck I, Engelbeen K, Vancanneyt M, Swings J *et al* (2009). Pantoea vagans sp nov., Pantoea eucalypti sp nov., Pantoea deleyi sp nov and Pantoea anthophila sp nov. *Int J Syst Evol Micr* **59:** 2339-2345.

Broda DM, Saul DJ, Bell RG, Musgrave DR (2000). Clostridium algidixylanolyticum sp nov., a psychrotolerant, xylan-degrading, spore-forming bacterium. *Int J Syst Evol Micr* **50:** 623-631.

Butt MS, Tahir-Nadeem M, Ahmad Z, Sultan MT (2008). Xylanases and their applications in baking industry. *Food Technol Biotech* **46:** 22-31.

Cailliez F (1983). The Analytical Solution of the Additive Constant Problem. *Psychometrika* **48:** 305-308.

Cantarel BL, Coutinho PM, Rancurel C, Bernard T, Lombard V, Henrissat B (2009). The Carbohydrate-Active EnZymes database (CAZy): an expert resource for Glycogenomics. *Nucleic Acids Res* **37:** D233-D238.

Cascales E, Buchanan SK, Duche D, Kleanthous C, Lloubes R, Postle K *et al* (2007). Colicin biology. *Microbiol Mol Biol Rev* **71:** 158-229.

Caspi R, Billington R, Ferrer L, Foerster H, Fulcher CA, Keseler IM *et al* (2016). The MetaCyc database of metabolic pathways and enzymes and the BioCyc collection of pathway/genome databases. *Nucleic Acids Res* **44:** D471-D480.

Chamkha M, Garcia JL, Labat M (2001). Metabolism of cinnamic acids by some Clostridiales and emendation of the descriptions of Clostridium aerotolerans, Clostridium celerecrescens and Clostridium xylanolyticum. *Int J Syst Evol Micr* **51:** 2105-2111.

Chassard C, Goumy V, Leclerc M, Del'homme C, Bernalier-Donadille A (2007). Characterization of the xylan-degrading microbial community from human faeces. *Fems Microbiology Ecology* **61:** 121-131.

Chassard C, Delmas E, Robert C, Lawson PA, Bernalier-Donadille A (2012). Ruminococcus champanellensis sp nov., a cellulose-degrading bacterium from human gut microbiota. *Int J Syst Evol Micr* **62:** 138-143.

Chen H (2016). VennDiagram: Generate High-Resolution Venn and Euler Plots. R package version 1.6.17. <https://CRAN.R-project.org/package=VennDiagram>.

Chen W, Zhang CK, Cheng Y, Zhang S, Zhao H (2013). A comparison of methods for clustering 16S rRNA sequences into OTUs. *PLoS One* **8:** e70837.

Choi JH, Lee KM, Lee MK, Cha CJ, Kim GB (2014). Bifidobacterium faecale sp nov., isolated from human faeces. *Int J Syst Evol Micr* **64:** 3134-3139.

Chung WS, Walker AW, Louis P, Parkhill J, Vermeiren J, Bosscher D *et al* (2016). Modulation of the human gut microbiota by dietary fibres occurs at the species level. *BMC Biol* **14:** 3.

Clark DP (1989). The Fermentation Pathways of Escherichia-Coli. *Fems Microbiol Lett* **63:** 223-234.

Cole JR, Wang Q, Fish JA, Chai BL, McGarrell DM, Sun YN *et al* (2014). Ribosomal Database Project: data and tools for high throughput rRNA analysis. *Nucleic Acids Res* **42:** D633-D642.

Cox TF (2001). Multidimensional scaling used in multivariate statistical process control. *J Appl Stat* **28:** 365-378.

Coyne MJ, Comstock LE (2008). Niche-specific features of the intestinal Bacteroidales. *J Bacteriol* **190:** 736-742.

Dabek M, McCrae SI, Stevens VJ, Duncan SH, Louis P (2008). Distribution of beta-glucosidase and beta-glucuronidase activity and of beta-glucuronidase gene gus in human colonic bacteria. *Fems Microbiology Ecology* **66:** 487-495.

Dehoux P, Marvaud JC, Abouelleil A, Earl AM, Lambert T, Dauga C (2016). Comparative genomics of Clostridium bolteae and Clostridium clostridioforme reveals species-specific genomic properties and numerous putative antibiotic resistance determinants. *Bmc Genomics* **17**.

Diaz PI, Zilm PS, Rogers AH (2000). The response to oxidative stress of Fusobacterium nucleatum grown in continuous culture. *Fems Microbiol Lett* **187:** 31-34.

Diaz PI, Zilm PS, Rogers AH (2002). Fusobacterium nucleatum supports the growth of Porphyromonas gingivalis in oxygenated and carbon-dioxide-depleted environments. *Microbiol-Sgm* **148:** 467-472.

Dodd D, Cann IKO (2009). Enzymatic deconstruction of xylan for biofuel production. *Gcb Bioenergy* **1:** 2-17.

Dodd D, Mackie RI, Cann IKO (2011). Xylan degradation, a metabolic property shared by rumen and human colonic Bacteroidetes. *Mol Microbiol* **79:** 292-304.

Duncan SH, Aminov RI, Scott KP, Louis P, Stanton TB, Flint HJ (2006). Proposal of Roseburia faecis sp nov., Roseburia hominis sp nov and Roseburia inulinivorans sp nov., based on isolates from human faeces. *Int J Syst Evol Micr* **56:** 2437-2441.

Duncan SH, Louis P, Thomson JM, Flint HJ (2009). The role of pH in determining the species composition of the human colonic microbiota. *Environ Microbiol* **11:** 2112-2122.

Duncan SH, Russell WR, Quartieri A, Rossi M, Parkhill J, Walker AW *et al* (2016). Wheat bran promotes enrichment within the human colonic microbiota of butyrate-producing bacteria that release ferulic acid. *Environ Microbiol* **18:** 2214-2225.

Edgar RC, Haas BJ, Clemente JC, Quince C, Knight R (2011). UCHIME improves sensitivity and speed of chimera detection. *Bioinformatics* **27:** 2194-2200.

El Kaoutari A, Armougom F, Gordon JI, Raoult D, Henrissat B (2013). The abundance and variety of carbohydrate-active enzymes in the human gut microbiota. *Nat Rev Microbiol* **11:** 497-504.

Farias LD, de Carvalho MAR, Houw H, de Oliveira AAP, Rodrigues PH, de Farias FF *et al* (1999). Atmospheric oxygen sensitivity of Fusobacterium strains. *Anaerobe* **5:** 157-159.

Farmer JJ, Fanning GR, Davis BR, Ohara CM, Riddle C, Hickmanbrenner FW *et al* (1985). Escherichia-Fergusonii and Enterobacter-Taylorae, 2 New Species of Enterobacteriaceae Isolated from Clinical Specimens. *J Clin Microbiol* **21:** 77-81.

Faust K, Sathirapongsasuti JF, Izard J, Segata N, Gevers D, Raes J *et al* (2012). Microbial Co-occurrence Relationships in the Human Microbiome. *Plos Comput Biol* **8**.

Faust K, Lahti L, Gonze D, de Vos WM, Raes J (2015). Metagenomics meets time series analysis: unraveling microbial community dynamics. *Curr Opin Microbiol* **25:** 56-66.

Faust K, Raes J (2016). CoNet app: inference of biological association networks using Cytoscape. *F1000Res* **5:** 1519.

Flint HJ, Bayer EA, Rincon MT, Lamed R, White BA (2008). Polysaccharide utilization by gut bacteria: potential for new insights from genomic analysis. *Nat Rev Microbiol* **6:** 121-131.

Flint HJ, Scott KP, Duncan SH, Louis P, Forano E (2012). Microbial degradation of complex carbohydrates in the gut. *Gut Microbes* **3:** 289-306.

Gaastra W, Kusters JG, Van Duijkeren E, Lipman LJA (2014). Escherichia fergusonii. *Vet Microbiol* **172:** 7-12.

Gao D, Luan Y, Wang Q, Liang Q, Qi Q (2015). Construction of cellulose-utilizing Escherichia coli based on a secretable cellulase. *Microb Cell Fact* **14:** 159.

Gower JC (1966). Some Distance Properties of Latent Root and Vector Methods Used in Multivariate Analysis. *Biometrika* **53:** 325-&.

Guimarães LH (2012). Carbohydrates from Biomass: Sources and Transformation by Microbial Enzymes. In: Chang CF (ed). *Carbohydrates - Comprehensive Studies on Glycobiology and Glycotechnology*.

Hale TL, Keusch GT (1996). Shigella. In: Baron S (ed). *Medical Microbiology*, 4th edn: Galveston (TX).

Han YPW (2015). Fusobacterium nucleatum: a commensal-turned pathogen. *Curr Opin Microbiol* **23:** 141-147.

Hatziioanou D, Mayer MJ, Duncan SH, Flint HJ, Narbad A (2013). A representative of the dominant human colonic Firmicutes, Roseburia faecis M72/1, forms a novel bacteriocin-like substance. *Anaerobe* **23:** 5-8.

Hayashi H, Shibata K, Sakamoto M, Tomita S, Benno Y (2007). Prevotella copri sp nov and Prevotella stercorea sp nov., isolated from human faeces. *Int J Syst Evol Micr* **57:** 941-946.

Hemdane S, Jacobs PJ, Dornez E, Verspreet J, Delcour JA, Courtin CM (2016). Wheat (Triticum aestivum L.) Bran in Bread Making: A Critical Review. *Comprehensive Reviews in Food Science and Food Safety* **15:** 28-42.

Henrissat B (1991). A Classification of Glycosyl Hydrolases Based on Amino-Acid-Sequence Similarities. *Biochem J* **280:** 309-316.

Henrissat B, Davies G (1997). Structural and sequence-based classification of glycoside hydrolases. *Curr Opin Struc Biol* **7:** 637-644.

Holdeman LV, Moore WEC (1974). New Genus, Coprococcus, 12 New Species, and Emended Descriptions of 4 Previously Described Species of Bacteria from Human Feces. *Int J Syst Bacteriol* **24:** 260-277.

Hong PY, Iakiviak M, Dodd D, Zhang ML, Mackie RI, Cann I (2014). Two New Xylanases with Different Substrate Specificities from the Human Gut Bacterium Bacteroides intestinalis DSM 17393. *Appl Environ Microb* **80:** 2084-2093.

Iuchi S, Weiner L (1996). Cellular and Molecular Physiology of Escherichia coli in the Adaptation to Aerobic Environments. *The Journal of Biochemistry* **120:** 1055-1063.

Johnson JL, Ault DA (1978). Taxonomy of Bacteroides .2. Correlation of Phenotypic Characteristics with Deoxyribonucleic-Acid Homology Groupings for Bacteroides-Fragilis and Other Saccharolytic Bacteroides Species. *Int J Syst Bacteriol* **28:** 257-268.

Johnson JL, Moore WEC, Moore LVH (1986). Bacteroides-Caccae Sp-Nov, Bacteroides-Merdae Sp-Nov, and Bacteroides-Stercoris Sp-Nov Isolated from Human Feces. *Int J Syst Bacteriol* **36:** 499-501.

Kennedy JF, Cabalda VM, White CA (1988). Enzymic Starch Utilization and Genetic-Engineering. *Trends Biotechnol* **6:** 184-189.

Killer J, Sedlacek I, Rada V, Havlik J, Kopecny J (2013). Reclassification of Bifidobacterium stercoris Kim et al. 2010 as a later heterotypic synonym of Bifidobacterium adolescentis. *Int J Syst Evol Micr* **63:** 4350-4353.

Lagaert S, Pollet A, Delcour JA, Lavigne R, Courtin CM, Volckaert G (2011). Characterization of two beta-xylosidases from Bifidobacterium adolescentis and their contribution to the hydrolysis of prebiotic xylooligosaccharides. *Appl Microbiol Biot* **92:** 1179-1185.

Lawley TD, Walker AW (2013). Intestinal colonization resistance. *Immunology* **138:** 1-11.

Le Bouguenec C (2005). Adhesins and invasins of pathogenic Escherichia coli. *Int J Med Microbiol* **295:** 471-478.

Lee GH, Rhee MS, Chang DH, Lee J, Kim S, Yoon MH *et al* (2013). Oscillibacter ruminantium sp. nov., isolated from the rumen of Korean native cattle. *Int J Syst Evol Microbiol* **63:** 1942-1946.

Lombard V, Ramulu HG, Drula E, Coutinho PM, Henrissat B (2014). The carbohydrate-active enzymes database (CAZy) in 2013. *Nucleic Acids Res* **42:** D490-D495.

Louis P, Flint HJ (2009). Diversity, metabolism and microbial ecology of butyrate-producing bacteria from the human large intestine. *Fems Microbiol Lett* **294:** 1-8.

Madej T, Lanczycki CJ, Zhang DC, Thiessen PA, Geer RC, Marchler-Bauer A *et al* (2014). MMDB and VAST+: tracking structural similarities between macromolecular complexes. *Nucleic Acids Res* **42:** D297-D303.

Maechler M, Rousseeuw P, Struyf A, Hubert M, Hornik K (2017). cluster: Cluster Analysis Basics and Extensions. R package version 2.0.6.

Magnúsdóttir S, Heinken A, Kutt L, Ravcheev DA, Bauer E, Noronha A *et al* (2017). Generation of genome-scale metabolic reconstructions for 773 members of the human gut microbiota. *Nat Biotechnol* **35:** 81-89.

Mangels JI, Lindberg LH, Vosti KL (1978). Quantitative-Evaluation of 3 Commercial Blood Culture Media for Growth of Anaerobic Organisms. *J Clin Microbiol* **7:** 59-62.

Martens EC, Lowe EC, Chiang H, Pudlo NA, Wu M, McNulty NP *et al* (2011). Recognition and Degradation of Plant Cell Wall Polysaccharides by Two Human Gut Symbionts. *Plos Biol* **9**.

Martin SA, Morrison WH, Akin DE (1998). Fermentation of maize bran, oat bran, and wheat bran by Bacteroides ovatus V975. *Curr Microbiol* **36:** 90-95.

Marzorati M, Vilchez-Vargas R, Bussche JV, Truchado P, Jauregui R, El Hage RA *et al* (2017). High-fiber and high-protein diets shape different gut microbial communities, which ecologically behave similarly under stress conditions, as shown in a gastrointestinal simulator. *Mol Nutr Food Res* **61**.

Mazur O, Zimmer J (2011). Apo- and cellopentaose-bound structures of the bacterial cellulose synthase subunit BcsZ. *J Biol Chem* **286:** 17601-17606.

McBain AJ, Macfarlane GT (1998). Ecological and physiological studies on large intestinal bacteria in relation to production of hydrolytic and reductive enzymes involved in formation of genotoxic metabolites. *J Med Microbiol* **47:** 407-416.

McGuire AM, Cochrane K, Griggs AD, Haas BJ, Abeel T, Zeng QD *et al* (2014). Evolution of Invasion in a Diverse Set of Fusobacterium Species. *Mbio* **5**.

McMurdie PJ, Holmes S (2014). Waste Not, Want Not: Why Rarefying Microbiome Data Is Inadmissible. *Plos Comput Biol* **10**.

McNulty NP, Wu M, Erickson AR, Pan CL, Erickson BK, Martens EC *et al* (2013). Effects of Diet on Resource Utilization by a Model Human Gut Microbiota Containing Bacteroides cellulosilyticus WH2, a Symbiont with an Extensive Glycobiome. *Plos Biol* **11**.

Mirande C, Kadlecikova E, Matulova M, Capek P, Bernalier-Donadille A, Forano E *et al* (2010). Dietary fibre degradation and fermentation by two xylanolytic bacteria Bacteroides xylanisolvens XB1AT and Roseburia intestinalis XB6B4 from the human intestine. *J Appl Microbiol* **109:** 451-460.

Mohan R, Namsolleck P, Lawson PA, Osterhoff M, Collins MD, Alpert CA *et al* (2006). Clostridium asparagiforme sp. nov., isolated from a human faecal sample. *Syst Appl Microbiol* **29:** 292-299.

Moore WEC, Johnson JL, Holdeman LV (1976). Emendation of Bacteroidaceae and Butyrivibrio and Descriptions of Desulfomonas Gen-Nov and 10 New Species in Genera Desulfomonas, Butyrivibrio, Eubacterium, Clostridium, and Ruminococcus. *Int J Syst Bacteriol* **26:** 238-252.

Morais S, Ben David Y, Bensoussan L, Duncan SH, Koropatkin NM, Martens EC *et al* (2016). Enzymatic profiling of cellulosomal enzymes from the human gut bacterium, Ruminococcus champanellensis, reveals a fine-tuned system for cohesin-dockerin recognition. *Environ Microbiol* **18:** 542-556.

Morris JH, Apeltsin L, Newman AM, Baumbach J, Wittkop T, Su G *et al* (2011). clusterMaker: a multi-algorithm clustering plugin for Cytoscape. *Bmc Bioinformatics* **12**.

Morris RL, Schmidt TM (2013). Shallow breathing: bacterial life at low O-2. *Nature Reviews Microbiology* **11:** 205-212.

Nakano V, Piazza RMF, Cianciarullo AM, Bueris V, Santos MF, Menezes MA *et al* (2008). Adherence and invasion of Bacteroidales isolated from the human intestinal tract. *Clin Microbiol Infec* **14:** 955-963.

Nakano V, Ignacio A, Fernandes MR, Fukugaiti MH, Avila-Campos MJ (2013). Intestinal Bacteroides and Parabacteroides species producing antagonistic substances. *Current Trends in Microbiology*.

Newman MEJ (2006). Finding community structure in networks using the eigenvectors of matrices. *Phys Rev E* **74**.

Oksanen J, Blanchet G, Friendly M, Kindt R, Legendre P, McGlinn D *et al* (2016). vegan: Community Ecology Package. R package version 2.4-0. <https://CRAN.R-project.org/package=vegan>.

Park J, Shokeen B, Haake SK, Lux R (2016). Characterization of Fusobacterium nucleatum ATCC 23726 adhesins involved in strain-specific attachment to Porphyromonas gingivalis. *Int J Oral Sci* **8:** 138-144.

Portrait V, Cottenceau G, Pons AM (2000). A Fusobacterium mortiferum strain produces a bacteriocin-like substance(s) inhibiting Salmonella enteritidis. *Lett Appl Microbiol* **31:** 115-117.

Presser KA, Ratkowsky DA, Ross T (1997). Modelling the growth rate of Escherichia coli as a function of pH and lactic acid concentration. *Appl Environ Microb* **63:** 2355-2360.

Quast C, Pruesse E, Yilmaz P, Gerken J, Schweer T, Yarza P *et al* (2013). The SILVA ribosomal RNA gene database project: improved data processing and web-based tools. *Nucleic Acids Res* **41:** D590-D596.

Quiroz-Castañeda RE, Folch-Mallol JL (2013). Hydrolysis of Biomass Mediated by Cellulases for the Production of Sugars. In: Chandel AK, Silva SSd (eds). *Sustainable Degradation of Lignocellulosic Biomass - Techniques, Applications and Commercialization*. InTech: Rijeka. p Ch. 06.

R Core Team (2016). R: A language and environment for statistical computing. R Foundation for Statistical Computing, Vienna, Austria. <https://www.R-project.org/>.

Ramette A (2007). Multivariate analyses in microbial ecology. *Fems Microbiology Ecology* **62:** 142-160.

Reichardt N, Duncan SH, Young P, Belenguer A, Leitch CM, Scott KP *et al* (2014). Phylogenetic distribution of three pathways for propionate production within the human gut microbiota. *Isme J* **8:** 1323-1335.

Richardson AJ, McKain N, Wallace RJ (2013). Ammonia production by human faecal bacteria, and the enumeration, isolation and characterization of bacteria capable of growth on peptides and amino acids. *Bmc Microbiol* **13**.

Rios-Covian D, Gueimonde M, Duncan SH, Flint HJ, de los Reyes-Gavilan CG (2015). Enhanced butyrate formation by cross-feeding between Faecalibacterium prausnitzii and Bifidobacterium adolescentis. *Fems Microbiol Lett* **362**.

Robrish SA, Oliver C, Thompson J (1991). Sugar Metabolism by Fusobacteria - Regulation of Transport, Phosphorylation, and Polymer Formation by Fusobacterium-Mortiferum Atcc-25557. *Infect Immun* **59:** 4547-4554.

Rogers GM, Baecker AAW (1991). Clostridium-Xylanolyticum Sp-Nov, an Anaerobic Xylanolytic Bacterium from Decayed Pinus-Patula Wood Chips. *Int J Syst Bacteriol* **41:** 140-143.

Rogowski A, Briggs JA, Mortimer JC, Tryfona T, Terrapon N, Lowe EC *et al* (2016). Glycan complexity dictates microbial resource allocation in the large intestine (vol 6, 7481, 2015). *Nat Commun* **7**.

Rolfe MD, Rice CJ, Lucchini S, Pin C, Thompson A, Cameron AD *et al* (2012). Lag phase is a distinct growth phase that prepares bacteria for exponential growth and involves transient metal accumulation. *J Bacteriol* **194:** 686-701.

Rousseeuw PJ (1987). Silhouettes: A graphical aid to the interpretation and validation of cluster analysis. *Journal of Computational and Applied Mathematics* **20:** 53-65.

Rubin LG (1986). Comparison of *in vivo* and *in vitro* multiplication rates of Haemophilus influenzae type b. *Infect Immun* **52:** 911-913.

Said SE, Dickey DA (1984). Testing for Unit Roots in Autoregressive-Moving Average Models of Unknown Order. *Biometrika* **71:** 599-607.

Salamanca-Cardona L, Ashe CS, Stipanovic AJ, Nomura CT (2014). Enhanced production of polyhydroxyalkanoates (PHAs) from beechwood xylan by recombinant Escherichia coli. *Appl Microbiol Biotechnol* **98:** 831-842.

Salyers AA, Gherardini F, Obrien M (1981). Utilization of Xylan by 2 Species of Human Colonic Bacteroides. *Appl Environ Microb* **41:** 1065-1068.

Savard P, Roy D (2009). Determination of Differentially Expressed Genes Involved in Arabinoxylan Degradation by Bifidobacterium longum NCC2705 Using Real-Time RT-PCR. *Probiotics Antimicro* **1:** 121-129.

Schloss PD, Westcott SL, Ryabin T, Hall JR, Hartmann M, Hollister EB *et al* (2009). Introducing mothur: Open-Source, Platform-Independent, Community-Supported Software for Describing and Comparing Microbial Communities. *Appl Environ Microb* **75:** 7537-7541.

Schloss PD, Westcott SL (2011). Assessing and Improving Methods Used in Operational Taxonomic Unit-Based Approaches for 16S rRNA Gene Sequence Analysis. *Appl Environ Microb* **77:** 3219-3226.

Scott KP, Gratz SW, Sheridan PO, Flint HJ, Duncan SH (2013). The influence of diet on the gut microbiota. *Pharmacol Res* **69:** 52-60.

Shannon P, Markiel A, Ozier O, Baliga NS, Wang JT, Ramage D *et al* (2003). Cytoscape: A software environment for integrated models of biomolecular interaction networks. *Genome Res* **13:** 2498-2504.

Sheridan PO, Martin JC, Lawley TD, Browne HP, Harris HM, Bernalier-Donadille A *et al* (2016). Polysaccharide utilization loci and nutritional specialization in a dominant group of butyrate-producing human colonic Firmicutes. *Microb Genom* **2:** e000043.

Simpson PJ, Stanton C, Fitzgerald GF, Ross RP (2005). Intrinsic tolerance of Bifidobacterium species to heat and oxygen and survival following spray drying and storage. *J Appl Microbiol* **99:** 493-501.

Song YL, Liu CX, Molitoris DR, Tomzynski TJ, Lawson PA, Collins MD *et al* (2003). Clostridium bolteae sp nov., isolated from human sources. *Syst Appl Microbiol* **26:** 84-89.

Sonnenburg ED, Zheng HJ, Joglekar P, Higginbottom SK, Firbank SJ, Bolam DN *et al* (2010). Specificity of Polysaccharide Use in Intestinal Bacteroides Species Determines Diet-Induced Microbiota Alterations. *Cell* **141:** 1241-U1256.

Sousa MA, Mendes EN, Apolonio AC, Farias Lde M, Magalhaes PP (2010). Bacteriocin production by Shigella sonnei isolated from faeces of children with acute diarrhoea. *APMIS* **118:** 125-135.

Su G, Kuchinsky A, Morris JH, States DJ, Meng F (2010). GLay: community structure analysis of biological networks. *Bioinformatics* **26:** 3135-3137.

Szczepańska AM (2011). Functional metagenomic analysis of carbohydrate degrading enzymes from the human gut microbiota, University of East Anglia.

The HC, Thanh DP, Holt KE, Thomson NR, Baker S (2016). The genomic signatures of Shigella evolution, adaptation and geographical spread. *Nature Reviews Microbiology* **14:** 235-250.

Thompson J, Robrish SA, Bouma CL, Freedberg DI, Folk JE (1997). Phospho-beta-glucosidase from Fusobacterium mortiferum: purification, cloning, and inactivation by 6-phosphoglucono-delta-lactone. *J Bacteriol* **179:** 1636-1645.

Trapletti A, Hornik K (2017). tseries: Time Series Analysis and Computational Finance. R package version 0.10-42.<https://CRAN.R-project.org/package=tseries>.

Tripathi CK, Banga J, Mishra V (2012). Microbial heparin/heparan sulphate lyases: potential and applications. *Appl Microbiol Biotechnol* **94:** 307-321.

United States Department of Agriculture (USDA) (2016). National Nutrient Database for Standard Reference Release 28.

van den Broek LAM, Hinz SWA, Beldman G, Vincken JP, Voragen AGJ (2008). Bifidobacterium carbohydrases-their role in breakdown and synthesis of (potential) prebiotics. *Mol Nutr Food Res* **52:** 146-163.

van der Maarel MJ, van der Veen B, Uitdehaag JC, Leemhuis H, Dijkhuizen L (2002). Properties and applications of starch-converting enzymes of the alpha-amylase family. *J Biotechnol* **94:** 137-155.

van Heel AJ, de Jong A, Montalban-Lopez M, Kok J, Kuipers OP (2013). BAGEL3: Automated identification of genes encoding bacteriocins and (non-)bactericidal posttranslationally modified peptides. *Nucleic Acids Res* **41:** W448-453.

Walter J, Ley R (2011). The Human Gut Microbiome: Ecology and Recent Evolutionary Changes. *Annual Review of Microbiology, Vol 65* **65:** 411-429.

Wang Q, Garrity GM, Tiedje JM, Cole JR (2007). Naive Bayesian classifier for rapid assignment of rRNA sequences into the new bacterial taxonomy. *Appl Environ Microb* **73:** 5261-5267.

Wang XY, Cai YP, Sun YJ, Knight R, Mai V (2012). Secondary structure information does not improve OTU assignment for partial 16s rRNA sequences. *Isme J* **6:** 1277-1280.

Warnes GR, Bolker B, Bonebakker L, Gentleman R, Liaw WHA, Lumley T *et al* (2016). gplots: Various R Programming Tools for Plotting Data. gplots: Various R Programming Tools for Plotting Data. <https://CRAN.R-project.org/package=gplots>.

Weaver J, Whitehead TR, Cotta MA, Valentine PC, Salyers AA (1992). Genetic analysis of a locus on the Bacteroides ovatus chromosome which contains xylan utilization genes. *Appl Environ Microbiol* **58:** 2764-2770.

Westcott SL, Schloss PD (2017). OptiClust, an Improved Method for Assigning Amplicon-Based Sequence Data to Operational Taxonomic Units. *mSphere* **2:** e00073-00017.

Wexler HM (2007). Bacteroides: the good, the bad, and the nitty-gritty. *Clin Microbiol Rev* **20:** 593-+.

White BA, Lamed R, Bayer EA, Flint HJ (2014). Biomass Utilization by Gut Microbiomes. *Annu Rev Microbiol* **68:** 279-296.

Wickham H (2009). ggplot2: Elegant Graphics for Data Analysis.

Zaika LL, Engel LS, Kim AH, Palumbo SA (1989). Effect of Sodium-Chloride, Ph and Temperature on Growth of Shigella-Flexneri. *J Food Protect* **52:** 356-359.

Zhang M, Chekan JR, Dodd D, Hong PY, Radlinski L, Revindran V *et al* (2014). Xylan utilization in human gut commensal bacteria is orchestrated by unique modular organization of polysaccharide-degrading enzymes. *Proc Natl Acad Sci U S A* **111:** E3708-3717.

Zheng J, Ganzle MG, Lin XB, Ruan L, Sun M (2015). Diversity and dynamics of bacteriocins from human microbiome. *Environ Microbiol* **17:** 2133-2143.

Zilm PS, Bagley CJ, Rogers AH, Milne IR, Gully NJ (2007). The proteomic profile of Fusobacterium nucleatum is regulated by growth pH. *Microbiol-Sgm* **153:** 148-159.
